# Emerging Electrochromic Materials and Devices for Future Displays

**DOI:** 10.1021/acs.chemrev.1c01055

**Published:** 2022-08-18

**Authors:** Chang Gu, Ai-Bo Jia, Yu-Mo Zhang, Sean Xiao-An Zhang

**Affiliations:** State Key Lab of Supramolecular Structure and Materials, College of Chemistry, Jilin University, Changchun 130012, People’s Republic of China

## Abstract

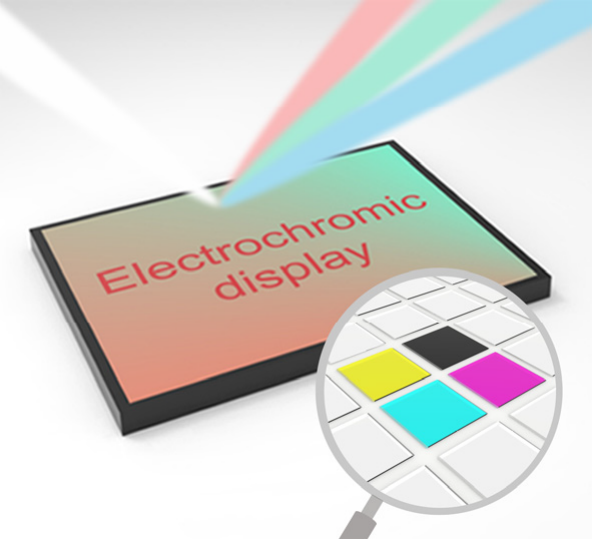

With the rapid development of optoelectronic fields, electrochromic (EC) materials and devices have received remarkable attention and have shown attractive potential for use in emerging wearable and portable electronics, electronic papers/billboards, see-through displays, and other new-generation displays, due to the advantages of low power consumption, easy viewing, flexibility, stretchability, etc. Despite continuous progress in related fields, determining how to make electrochromics truly meet the requirements of mature displays (e.g., ideal overall performance) has been a long-term problem. Therefore, the commercialization of relevant high-quality products is still in its infancy. In this review, we will focus on the progress in emerging EC materials and devices for potential displays, including two mainstream EC display prototypes (segmented displays and pixel displays) and their commercial applications. Among these topics, the related materials/devices, EC performance, construction approaches, and processing techniques are comprehensively disscussed and reviewed. We also outline the current barriers with possible solutions and discuss the future of this field.

## 1 Introduction

**Figure 1:**
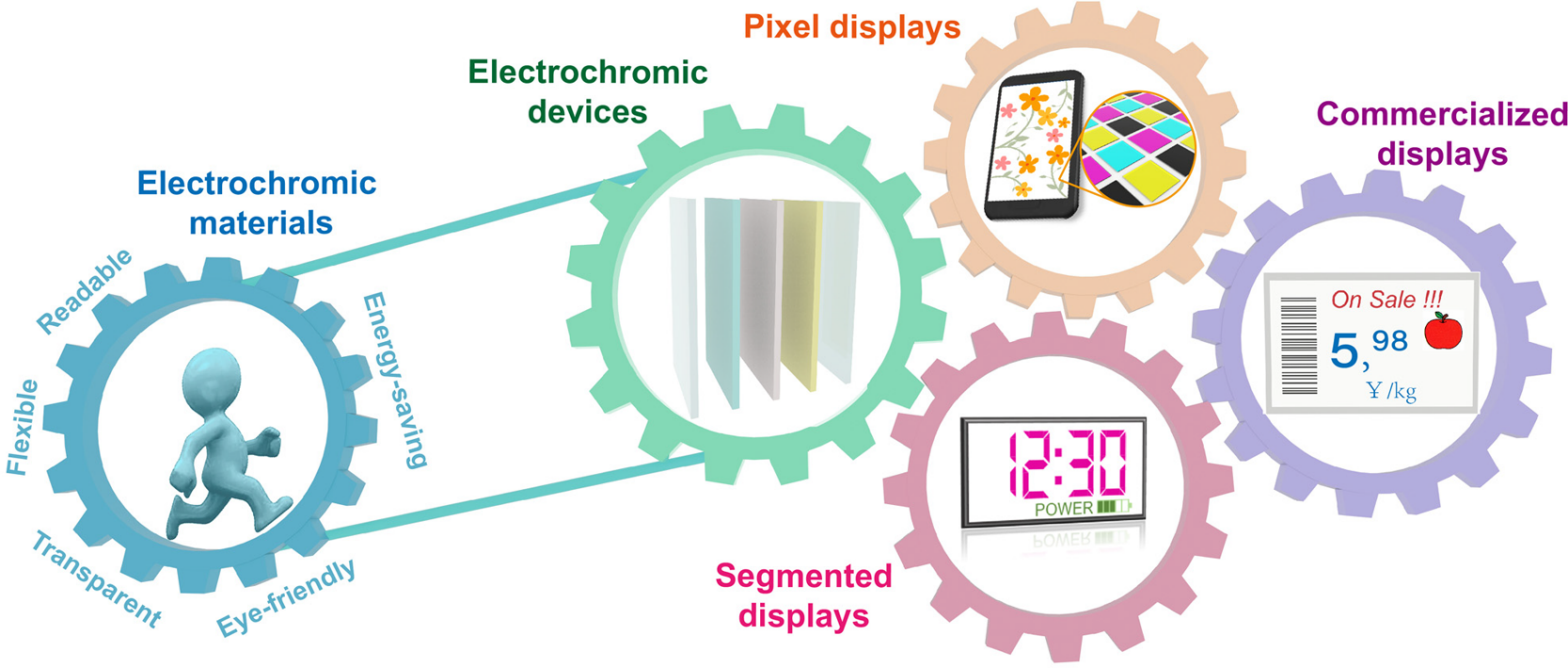
Advantages and values of electrochromic materials and corresponding developmental trajectory from electrochromic devices to mainstream display prototypes (segmented displays and pixel displays) and commercialized displays.

With the continuous advancement of modern display technologies,^1−9^ the electrochromic (EC) display, a typical nonemissive (passive) display, has received extensive attention.^10−39^ The working principle of EC displays is based on the electrochemically driven redox process of EC materials to produce color/transmittance/reflectivity changes and present the displayed content and information. Different from commercialized active light-emitting displays such as cathode ray tube (CRT) displays,^40^ liquid crystal displays (LCDs),^41^ light-emitting diode (LED) displays,^42,43^ organic light-emitting diode (OLED) displays,^44,45^ and quantum-dot light-emitting diode (QLED) displays,^46,47^ and known nonemissive displays such as electrophoretic and dielectrophoretic displays,^48^ electrowetting displays,^49,50^ interferometric modulator (IMOD) displays^51^ and photonic crystal displays,^52^ the current research and development (R&D) process of EC displays is still in the early stage. And the commercialization of relevant high-quality products still has a long way to go. Fortunately, due to many unique advantages and potential values demonstrated by pioneering studies (shown below and in Figure 1),^53−55^ EC displays are expected to be one of next-generation displays that people have long-awaited.

First, EC displays have ideal outdoor readability and eye-friendly features due to the subtractive color mode (light-absorbing mode). Therefore, the observed content and information do not come from the luminescence but from the color change in related materials under electrochemically driven redox processes. This efficiently avoids the radiation damage of strong blue light to human eyes and enables a comfortable and pleasant reading experience under strong ambient light. Second, EC displays feature flexibility, scalability, foldability, and transparency. Most existing EC materials maintain good compatibility with multiple substrates including glass, metal, plastic, and even fibers and textiles,^56−59^ which meets the needs of anticipated comfortable and portable electronics, for instance, wearable sensors.^60−62^ Third, low energy consumption compared with existing displays (e.g., LCDs and OLEDs) has been demonstrated owing to their optical memory effect. The optical memory effect refers to the phenomenon that EC materials and devices can maintain their optical states for a period of time without continuous input of electrical power. Therefore, EC materials/devices show outstanding potential for utilization in super energy-saving displays with low-frequency information refresh (for instance, billboards/labels, electronic paper, etc.), and even information storage. Note that electrofluorochromism, which is defined as the phenomenon in which the fluorescence of a material produces significant changes in intensity or color under an electrical stimulus, is an important extension of electrochromism. The first example of an electrofluorochromic (EFC) window was reported by Pierre Audebert and Eunkyoung Kim in 2006.^63^ Since then, EFC materials and devices have attracted extensive research interest.^64−67^ Electrofluorochromism enriches the application scenarios of EC displays under dark conditions due to the emission mode. At the same time, the dual modes of electrical stimulation and light stimulation (of the excitation beam) required for the information display enhance its potential for anticounterfeiting, encryption, and analytical applications (e.g., biosensors).

There are currently two main display prototypes: segmented displays and pixel displays, as shown in Figure 1. These prototypes are divided according to their different working principles and information capacities, which will be discussed in detail later. Compared to other EC applications (smart windows, antiglare rearview mirrors, etc.), ideal EC displays have higher performance requirements, for example, faster response speed for the fast information displaying/switching capability, better stability and durability to support their lifetime, and higher optical contrast to ensure an enjoyable reading experience. Admittedly, the practical application of EC displays still has a long way to reach due to the immature and imperfect performance at this stage.

On the other hand, unfortunately, there is little systematic and overall design guidance. The only review focused on this topic was written 16 years ago by John R. Reynolds et al.^68^ Therefore, there is an urgent need to address this deficiency. In this review, we focus on advances in emerging EC materials and devices that have been presented over the last decade (from 2011 to 2021), as well as their prototypes (segmented displays and pixel displays) for future commercialized applications. The corresponding references are not strictly arranged by year of publication but are listed according to the content of discussion. Meanwhile, the developments, tendencies, and existing issues are discussed in detail based on the reported studies. To better understand this field, the discussion is divided into the following sections: EC materials and devices (including classic EC materials and devices, performance index, and emerging materials and devices), EC segmented displays (including electrode patterning and active material patterning), EC pixel displays (including pixelated EC materials and the driving mode), and corresponding progress and challenges of EC displays in future commercial development.

## 2 Electrochromic Materials and Devices

### 2.1 Classic Electrochromic Materials and Devices

**Figure 2:**
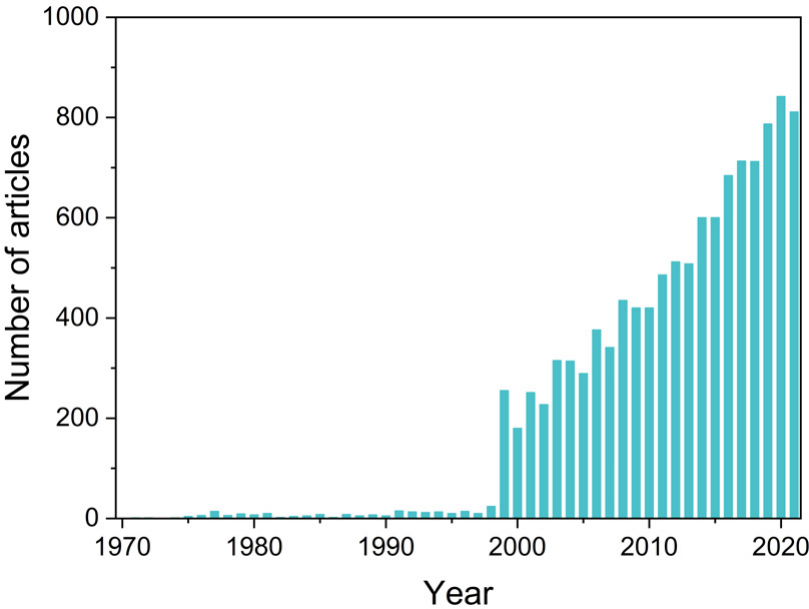
Number of published articles on EC fields from 1970 to 2021. Data was obtained from Web of Science. Topic = (“electrochromic” OR “electrochromism”) was used to obtain related articles.

EC materials, as typical stimuli-responsive smart materials,^69^ have a research history of approximately 60 years. In 1961, J. R. Platt defined the phenomenon in which the absorption spectra of some dyes might be shifted under a strong electric field as “electrochromism”.^70^ This is considered the birth of related fields. Subsequently, S. K. Deb prepared WO_3_-based EC films and conducted related research.^71,72^ In 1984, the concept of how to use WO_3_-based EC materials for energy-efficient windows (smart windows) was proposed and demonstrated by Claes G. Granqvist and Carl M. Lampert.^73,74^ As one of the most important applications of electrochromism, smart windows are expected to save energy and improve living comfort. To date, they are still attracting research attention around the world, and the corresponding commercialization is progressing continuously. At the same time, organic EC materials have also gradually emerged and been introduced by some representative reviews.^53,68,75,76^ Due to the unremitting efforts of outstanding pioneers,^77−83^ the field of electrochromism has achieved considerable development in recent years, and related reports have also been increasing rapidly, as shown in Figure 2.

Over the years, electrochromism has been broadened to inorganic materials (WO_3_,^84−86^ NiO,^87^ VO_*x*_,^88,89^ TiO_2_,^90^ Prussian blue (PB),^91^ etc.^92^), small organic molecules (viologens,^93^ organic redox dyes,^94−96^ etc.), conjugated polymers (CPs) (e.g., polyaniline and polythiophene),^97−101^ and metal–organic complexes.^102−106^ Related materials and their advantages/disadvantages have been systematically discussed/summarized many times.^107−112^ For example, inorganic EC materials have excellent photostability, but their response speed and color tunability are relatively limited. Small organic molecules have bright colors and good color tunability, but the small sizes and weights usually lead to undesired thermal diffusion and poor stability in devices. CPs have attractive electrical conductivities and are also convenient for preparing devices by solution-processing methods, but their imperfect spectral purity is still a vexing problem that limits the applications in colorful (or even full-color) displays. Metal–organic complexes or inorganic–organic complexes that combine the advantages of both material types to some degree. However, there are still urgent problems that need to be solved, for example, insufficient film-forming ability.

Existing EC materials can also be classified into cathode materials and anode materials according to the redox mode. For example, the dication form of viologens (initial state) shows an obvious color change under electrochemical reduction; this characteristic can be used to define cathode EC materials (electrochemical reduction mode). In contrast, 1,4-phenylenediamine derivatives undergo electrochemical oxidation to change from colorless states to colored states;^113^ this characteristic can be used to define anode EC materials (electrochemical oxidation mode). In addition, Pierre M. Beaujuge and John R. Reynolds introduced another classification system according to the color change: (i) at least one colored and one bleached state, (ii) two distinct colored states, and (iii) multicolored electrochromics.^53^ A classification system based on solubility has also been proposed.^54^ This classification system includes three categorical types. (i) Both the reduced state and oxidized state are soluble. For instance, most small organic molecules fall into this category. (ii) Only one redox state is soluble. For instance, EC electrodeposited materials/devices based on Ag^+^, Cu^2+^, Bi^3+^, etc., fall into this category. (iii) Both or all redox states are insoluble. For instance, most metal oxides (e.g., WO_3_), CPs, etc. fall into this category. In recent years, Sean X.-A. Zhang et al. summarized electrochromism into (i) the direct redox mode and (ii) the indirect redox mode.^114^ This classification system is based on the relationship between redox-active units and chromophores. For most EC materials, their redox-active units are the same as chromophores. In other words, the color change is caused by the electrochemically driven redox process of chromophores (the direct redox mode). For the other EC materials with the indirect redox mode, their redox-active units and chromophores are separated. When an electrochemically driven redox process occurs, proton transfer (or energy transfer^115^) and coordination interactions are formed to further induce related color changes of chromophores. Generally, these classification systems focus on different issues, and thus play different key roles in their respective occasions.

**Figure 3:**
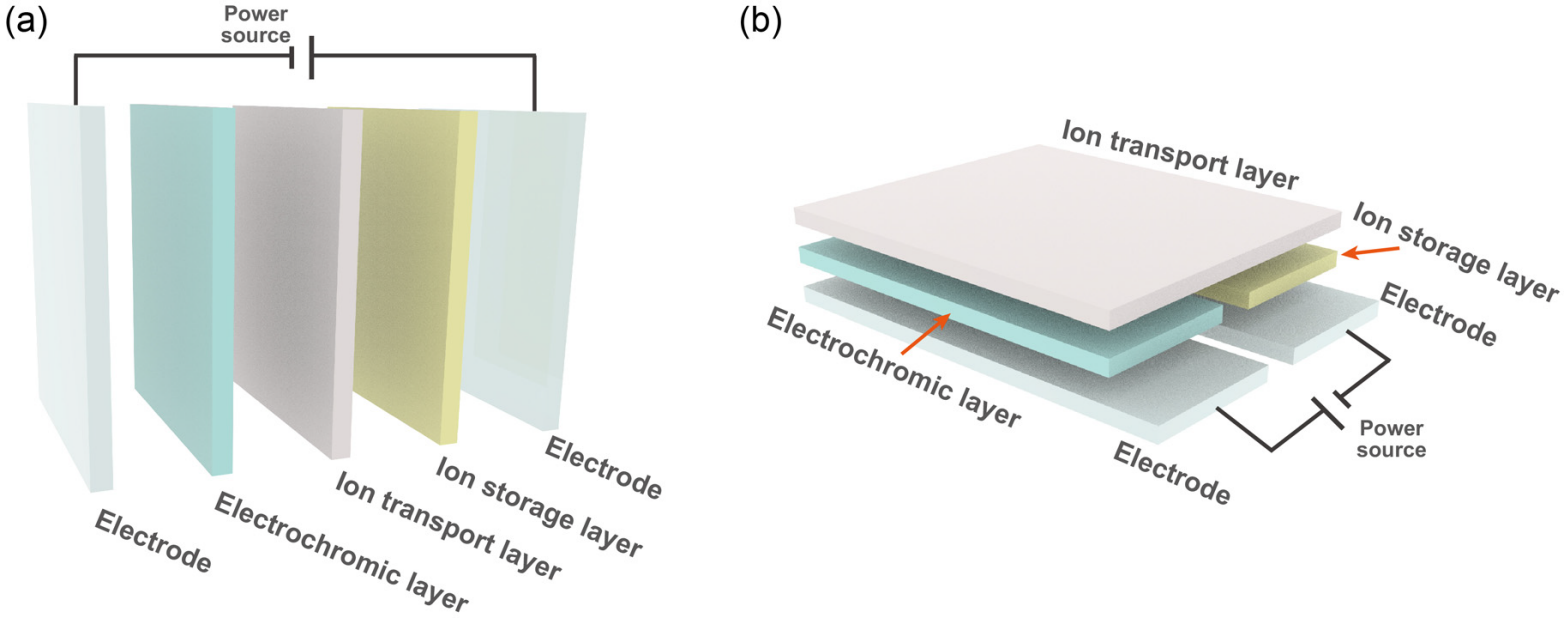
Structures of the classic (a) layered and (b) lateral ECD with five functional layers, including two electrodes, an ECL, an ITL, and an ISL.

In the practical applications of EC materials, especially the future/potential displays discussed in this review, electrochromic devices (ECDs) as the basic working module, need to be considered and manufactured. Mainstream ECDs contain five layers (working electrode/EC layer/ion transport layer/ion storage layer/counter electrode), as shown in Figure 3. The electrodes (working electrode and counter electrode) are located on either side of the device for electron and charge transfer. Reported candidates include indium tin oxide (ITO),^116,117^ fluorine-doped tin dioxide (FTO),^118^ ITO-coated polyethylene glycol terephthalate (PET-ITO),^119^ Ag nanowires (Ag NWs),^120−122^ conductive metal grids,^123,124^ graphene,^125−129^ carbon nanotubes,^130−132^ and their composite materials (e.g., Ag–Au core–shell nanowire networks^133^ and copper-nanowire-reduced-graphene-oxides^134^), etc.^135^ The ion storage layer (ISL) is used to balance charges via doped active materials; it undergoes reversible electrochemical oxidation (reduction) to match with the reduction (oxidation) of EC materials in the electrochromic layer (ECL). Thus, related materials for ISL must maintain good electrochemical reversibility, stability, capacity, and compatibility with EC materials.^136−140^ The ion transport layer (ITL) has the function of transferring ions inside the device. The candidates for related ion-transport materials include electrolytes (e.g., lithium salt, ammonium salt, and ionic liquid)-doped gels/solutions/films,^141−146^ polymers with ionic conductivity^147−150^ such as poly(ionic liquid) and Nafion, (ionic) liquid crystals,^151,152^ etc. The ECL, which is mainly composed of EC materials, is the core of an ECD; it undertakes the task of color change and optical modulation. In fact, the electrode, ISL, and ITL all have critical impacts on device performance. In this regard, we will discuss these components systematically in subsequent Section 4.2.3 and Section 5.2.1.

Currently, with the joint efforts of many outstanding scientists, research on EC materials and devices is not limited to the field of color changes observed by the naked eye. For example, modulation in near-infrared and infrared regions has also been extensively studied.^153−157^ Meanwhile, based on an identical working principle with the typical ECD with five functional layers, multiple device structures can be fabricated (such as all-solid devices,^158−162^ semisolid or gel devices,^163−165^ and liquid devices^166,167^).

### 2.2 Performance Index

**Figure 4:**
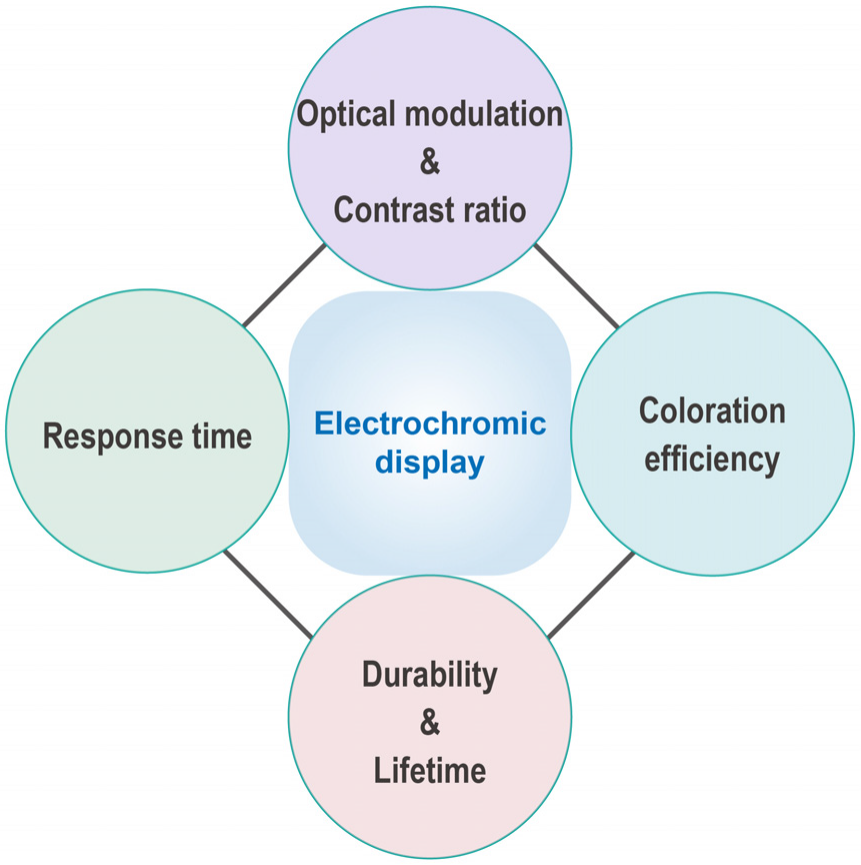
Performance indexes for EC display including optical modulation and contrast ratio, response time, coloration efficiency, and durability and lifetime.

To help quickly and accurately evaluate the performance of related EC materials and devices, some commonly used and important performance indexes including optical modulation and contrast ratio, response time, coloration efficiency, durability, and lifetime, are introduced here, as shown in Figure 4. In recent decades, these indexes have been mentioned and discussed many times. Here, we focus on the display field. Note that some other performance indexes should be considered also in specific situations. For example, the optical memory effect and bistability should be considered when preparing EC static displays or energy storage devices. And color purity and color tunability should be considered for full-color displays.

#### 2.2.1 Optical Modulation and Contrast Ratio

Optical modulation is the primary parameter for demonstrating the color-switching ability of an EC material/device. It is defined as the difference in absorbance or transmittance at the characteristic absorption wavelength before and after color switching, as shown in Equation 1.
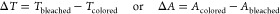
1

Here, *T*_colored_, *T*_bleached_, *A*_colored_, and *A*_bleached_ represent the transmittance in the colored state, transmittance in the bleached state, absorbance in the colored state, and absorbance in the bleached state, respectively. Δ*T* and Δ*A* represent the optical (transmittance and absorbance) modulations. For a reflection-type device, the difference in reflectivity can also be used to define its optical modulation.

The contrast ratio (CR) is another widely accepted performance index for evaluating the color-switching ability, as shown in Equation 2.
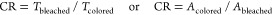
2

For an EC display, the optical modulation and CR need to be large enough to ensure an enjoyable reading experience. A higher optical modulation also increases the color-grading capacity to further enhance the grayscale display potential. Moreover, thanks to its light-absorbing (subtractive) mode, an EC display can maintain a high CR under strong ambient light (e.g., outdoors), which is an attractive feature with respect to light-emitting displays.

#### 2.2.2 Response Time

The response time is the required time for an ECD to reach 90% of its full optical modulation from the bleached state (colored state) to the colored state (bleached state). This is also called the coloring time (bleaching/fading time). Generally, EC materials/devices with shorter response times are more desirable.

Different from the EC materials/devices utilized in smart windows or energy storage devices, whose acceptable switching times are on the order of minutes, EC displays usually need to complete color switching within seconds (or even milliseconds) to meet the requirement of information refresh frequency. For example, in simple terms, a response time of no more than 16.7 ms should be reached for a device with a 60-Hz refresh rate. At present, such a fast response time is still a very large challenge for existing EC materials/devices. One of the main reasons for this is that electrochromics must undergo an electrochemically driven redox process, shown as the Faraday charging/discharging process. This process is usually accompanied by changes in the molecular structure (or crystal form) and ion transfer of electrolytes. Therefore, compared to existing light-emitting displays such as OLEDs, which only involve the migration and recombination of carriers, the response speeds of electrochromics are inevitably slower. Under this condition, in addition to developing high-performance EC materials/devices, various electrodes with extremely large active surface areas or novel materials with excellent ion conductivities have been studied.

However, the optical modulation (contrast ratio) of different devices varies greatly. Meanwhile, the reported response time of some ECDs is not based on achieving maximum (or appropriate) optical modulation. Therefore, it is difficult to compare the response time data horizontally. Based on this, the response speed is proposed as a new performance index taking the optical modulation into consideration, as shown in Equation 3.
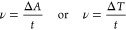
3Δ*T* and Δ*A* represent 90% of the optical modulation, and *t* represents the time spent.

#### 2.2.3 Coloration Efficiency

The coloration efficiency (CE) is defined in Equation 4 and represents the optical modulation at the characteristic absorption wavelength per injected charge in per unit area.
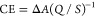
4

Where Δ*A*, *Q*, and *S* represent the optical modulation, injected charge, and active area, respectively. CE is a classic efficiency index; that is, higher optical modulations can be achieved by ECDs with higher CEs under the same amount of injected charge. In other words, an ECD with a higher CE requires less charge to achieve the same optical modulation. Obviously, EC materials/devices with higher CEs are more popular due to higher energy efficiencies.

#### 2.2.4 Durability and Lifetime

Durability refers to the ability of a material/device to withstand an unfavorable external environment. For example, the requirements of the international equipment durability standard, American Society for Testing and Materials (ASTM) E2141-14 for ECDs should be satisfied. An eligible EC display should also be able to work under certain extreme conditions including temperature (from −40 to 80 °C), humidity (reaching 80%), and even some degree of external force. At the same time, organic materials generally have imperfect photostabilities (as indicated by the corresponding photodegradation), especially to ultraviolet (UV) rays and oxygen, which is an inherent disadvantage that needs to be addressed urgently.^168,169^

Moreover, the lifetimes are also critical parameters. On one hand, the electro-optical switching capacity of an ECD should be maintained after coloring-bleaching cycles. To meet the demand of high-intensity information switching in future practical applications, the ideal reversibility (cyclic life) of EC displays should reach at least 10^4^–10^6^ cycles without significant optical degradation. On the other hand, the calendar life represents the working time of the material/device. Although this important index has been widely accepted in energy storage fields,^170−175^ it has been rarely discussed in EC displays and related research.

There is no doubt that the durability and lifetimes of EC devices (displays) are closely related to the properties of the relevant materials and the EC reaction mechanism used. Besides, they also tend to be closely related to the manufacturing process and quality of the device. In Section 5.2, we will discuss them from this aspect in conjunction with examples.

### 2.3 Emerging Materials and Devices

To date, the performance of traditional EC materials and devices still has difficulty meeting the practical requirements of displays at this stage. To address this problem and achieve a leapfrog development, some fascinating explorations on emerging EC materials and devices have been carried out with the following approaches. These approaches are state-of-the-art in related fields and have remarkable guiding significance for the follow-up research.

#### 2.3.1 Directional Optimization Based on the Reaction Process

**Figure 5:**
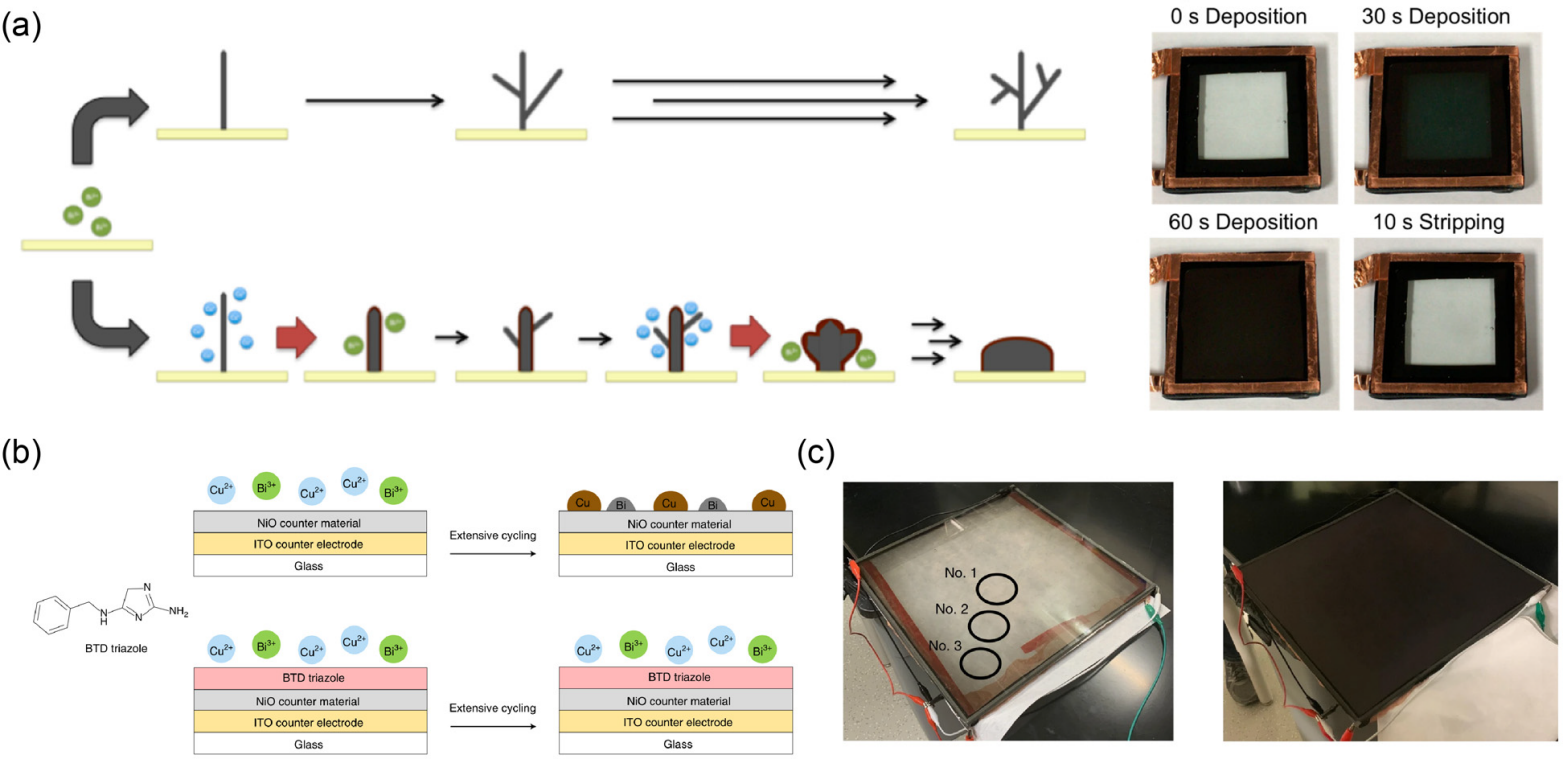
(a) Left: schematic of deposition of Bi without (top) and with (bottom) Cu. Green and blue balls represent Bi^3+^ and Cu^+^, respectively. Right: photos of prepared dynamic smart windows (25 cm^2^) under different states. Reproduced from ref (184). Copyright 2018 American Chemical Society. (b) Schematic of NiO-ITO counter electrode without (top) and with (bottom) BTD triazole after extensive cycling. Reproduced from ref (185). Copyright 2019 Springer Nature. (c) Dynamic window of 929 cm^2^ based on EC electrodeposition in clear state (left) and dark state (right). Reproduced from ref (186). Copyright 2021 Springer Nature.

The most applicable approach for solving current problems for the performance improvement is the directional optimization based on the reaction process. This helps to directly attack the pain points of existing materials/devices and achieve targeted optimization. Here, a classic example of this approach involves the methods for preventing harmful metal dendrite growth of electrodeposited ECDs.^176−178^ The harmful dendrite growth also widely exists in the field of metal batteries, especially Li-ion batteries.^179−183^ To efficiently solve this problem and further improve the performance of electrodeposition devices, it is urgent to perform an in-depth study of the dendritic growth mechanism and make targeted optimizations. When studying the Bi-based EC electrodeposition smart window, Michael D. McGehee et al. cleverly introduced Cu into the EC system and solved the above problems.^184^ In related electrochemical processes, the dendritic Bi atoms were easily oxidized by electrochemically generated Cu ions (Cu^+^) to Bi^3+^, which could be quickly dissolved and peeled off. Therefore, a Bi atom was replaced by three Cu atoms, and the voids in the dendritic Bi were quickly filled with newly generated Cu (Figure 5a). Based on this, the unwanted dendritic growth pathway was subtly transformed into a spherical particle growth pathway. And the as-prepared device exhibited more stable performance. The authors summarized this mechanism as that “the galvanic displacement of Bi by Cu induces spherical particle growth”. In 2019, they further discussed the influence of inhibitor on the electrodeposition of Bi/Cu (Figure 5b).^185^ In this work, BTD triazole was introduced as a small-molecule inhibitor to prevent unwanted metal deposition on the NiO-ITO counter electrode. The as-prepared device showed improved EC performance such as a high optical CR (75% in the clear state and 10% in the color-neutral black state) and a significant cyclic life (>4000 times). Recently, another inhibitor (poly(vinyl alcohol), PVA) was used to control the morphology of the electrodeposited metal film.^186^ Based on this, the device could switch to extremely low visible transmittance (below 0.001%) within 3 min, and could maintain fast response and excellent uniformity over a large area (>900 cm^2^) (Figure 5c).

**Figure 6:**
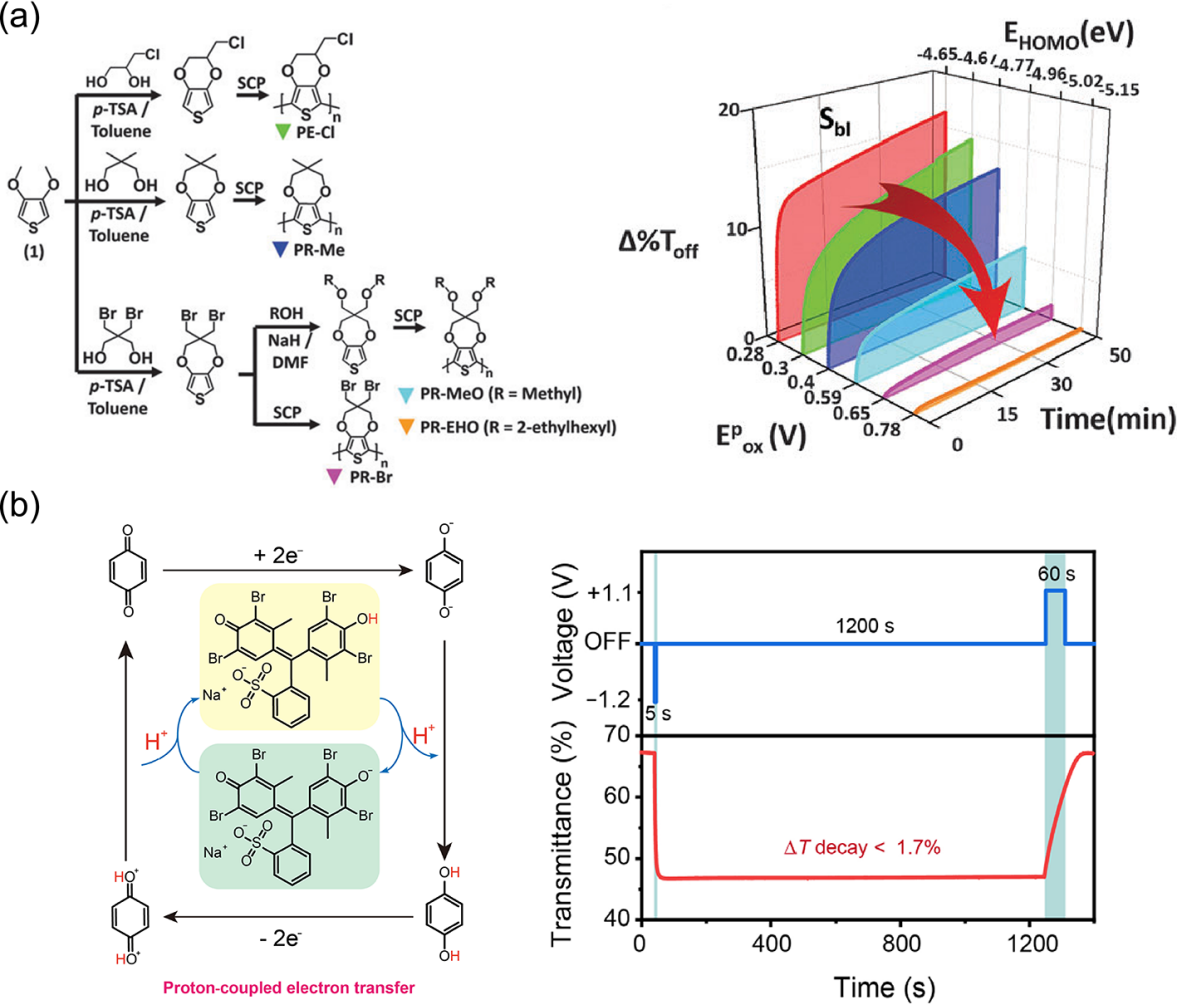
(a) Left: structures of EC CPs. Right: the Δ*T* of ECDs in voltage-off state based on CPs with different *E*^p^_ox_ and *E*_HOMO_ values. Reproduced from ref (187). Copyright 2016 Royal Society of Chemistry. (b) Left: EC mechanism involving intermolecular proton transfer. Right: transmittance changes in bistable ECD (bottom) under relative electrical stimulation (top). Reproduced from ref (188). Copyright 2021 Chinese Chemical Society.

Another classic example for the directional optimization is that determining how to further improve the optical memory effect (bistability). In fact, traditional EC systems (devices) are generally not ideally constructed and tend to leak charge. Thus, when the external potential is turned off, the residual charge and EC materials in high-energy states are usually prone to spontaneous reverse transfer of electrons. This phenomenon (also known as self-erasing) is sometimes beneficial, but must be avoided in bistable EC materials/devices. One of potential reasons for the unwanted charge leakage is the spontaneous electron transfer between EC materials and electrodes. For this reason, bistable EC materials should have appropriate energy levels compared to the Fermi energy level (*E*_F_) of electrodes, to limit this unwanted process. Such an energy level matching could be achieved by ingenious structural design and the introduction of suitable substituents, as reported by Eunkyoung Kim’s group in 2016.^187^ In that work, a series of CPs modified with different substituent groups (as EC materials) were designed and prepared (Figure 6a). The results revealed that ECDs based on these CPs exhibited different optical memory times. By analyzing the highest occupied molecular orbital (HOMO) energy levels of related EC materials, the authors found that the prepared device showed better bistability when the HOMO energy levels were lower than the *E*_F_ of the electrode. This work provides a very meaningful idea for a deeper understanding and optimization of EC bistability.

Furthermore, Sean X.-A. Zhang, Yu-Mo Zhang, and coauthors introduced an “active energy-exchange” strategy to limit unwanted electron reversal transfer.^188^ Through the process of intermolecular proton transfer (Figure 6b), the chemical energy of the molecules in a high-energy (unstable) state after an electrochemically driven redox process was actively released. This intermolecular energy conversion process effectively achieved energy level matching between EC materials and electrodes, and prevented unwanted interfacial electron transfer. Based on this result, remarkable bistability (Δ*T* decay <1.7% after 1200 s) was achieved.

In addition to the above-mentioned approach for bistable ECDs that achieve the energy level matching, two other approaches should also be considered. (i) Improving the chemical stability of related materials. That is, spontaneous molecular damage and deterioration should be avoided as much as possible. (ii) Reducing unwanted thermal diffusion (shuttle effect) of redox active molecules. After the electrochemically driven redox process, active small molecules tend to meet and generate electron transfer inside the device due to molecular diffusion, and return to their initial state even if the device is in an open-circuit state.

**Figure 7:**
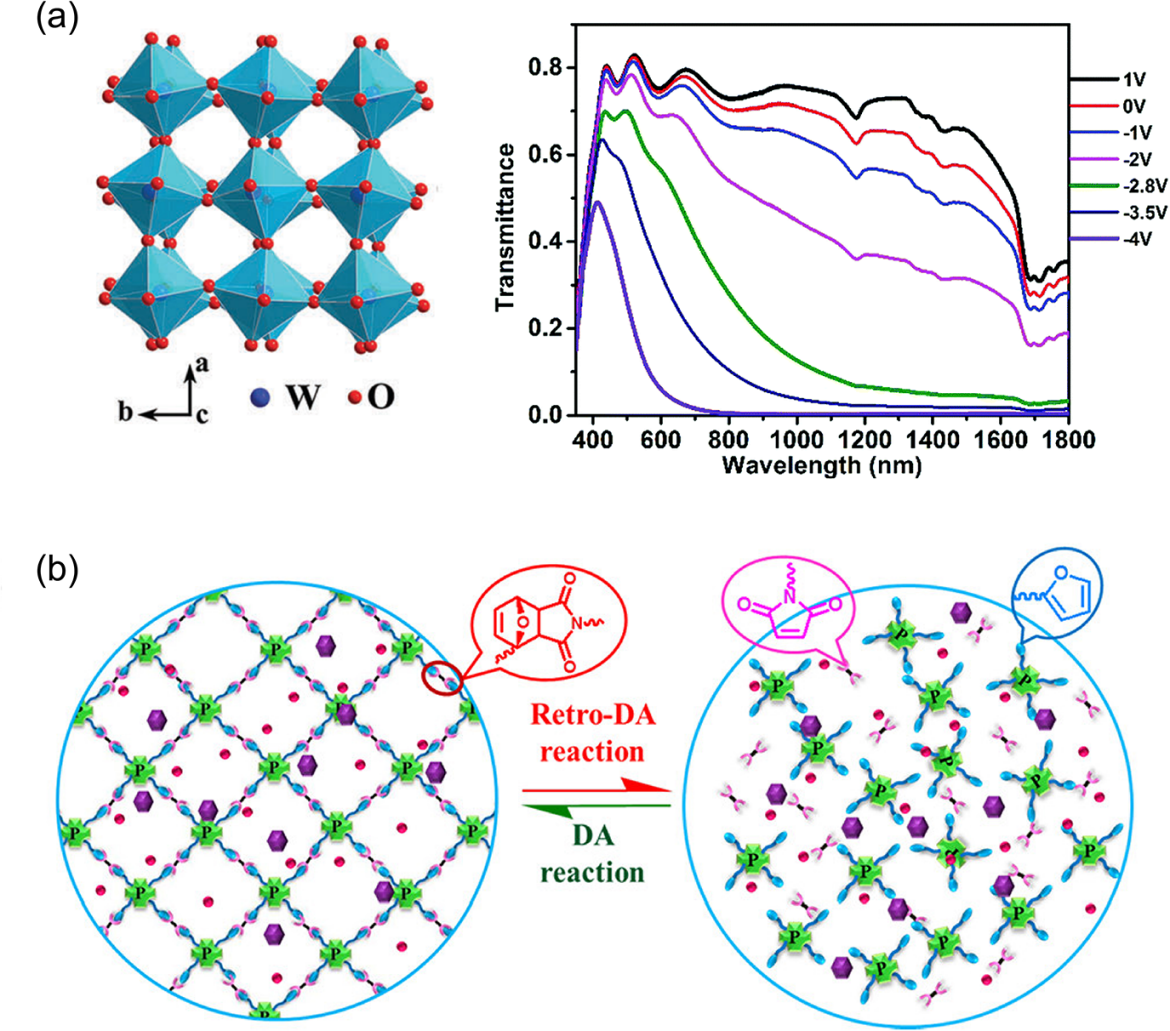
(a) Left: crystal structure of m-WO_3–*x*_ NWs. Right: transmittance spectra of a dual-band ECD with Al^3+^ electrolyte (1 M Al(ClO_4_)_3_ in PC). Reproduced from ref (190). Copyright 2018 Royal Society of Chemistry. (b) Schematic of self-healing performance based on reversible Diels–Alder cross-linking network. Reproduced from ref (192). Copyright 2020, American Chemical Society.

For WO_*x*_ EC materials, monovalent cations (H^+^, Li^+^, and Na^+^) are commonly used as the insertion ions. In 2015, Zhigang Zhao’s group cleverly replaced the auxiliary monovalent ions required for W_18_O_49_ nanowire EC films with trivalent Al^3+^ cations for the first time.^189^ The trivalent state of Al^3+^ could quickly share excess oxygen atoms with the surrounding low-valent transition metal oxides and showed stronger electrostatic forces than related monovalent ions. Such interactions significantly improved the performance of related ECDs. Three years later, Jim Y. Lee and coauthors further reported an Al^3+^-based ECD with even better EC performance,^190^ for example, a high optical modulation in the full solar spectrum (633 nm: 93.2%, 800 nm: 91.7%, 1200 nm: 88.5%, and 1600 nm: 86.8%) (Figure 7a) and remarkable CE, response speed, and bistability. The results further demonstrated that the multivalent state and small ion radius are possible reasons for the rapid and effective diffusion and the promotion of EC performance. Finally, the authors also developed a potential application as a dual-band EC smart window. Different from WO_*x*_ EC materials, the EC mechanism of Ni oxide is still unclear. In 2015, Rui-Tao Wen, Claes G. Granqvist, and Gunnar A. Niklasson proved that related EC process was mainly controlled by surface processes with both cation and anion exchanges.^191^ This research is of great significance to the development of Ni oxide-based EC materials.

The repeated insertion and extraction of auxiliary ions in the EC layer will damage its structure, which leads to a loss of EC performance. In this case, the introduction of self-healing properties is helpful. For example, Chunyang Jia’s group fabricated an all-in-one cross-linked self-healing device.^192^ In this article, methyl viologen (MV(PF_6_)_2_) was used as the cathodic EC material. The existence of poly-1,3,6,8-tetramino(furfuryl glycidyl ether)-pyrene-bismaleimide (TAFPy-MA) could repair the cracks in the EC layer (self-healing within 110 s) due to the corresponding reversible Diels–Alder cross-linking network (Figure 7b). Based on this, the as-prepared ECD showed reliable stability, for example, cyclic life (>1000). In addition, a large-area smart window prototype (30 × 35 cm^2^) was developed.

#### 2.3.2 Introducing Composite Materials

**Figure 8:**
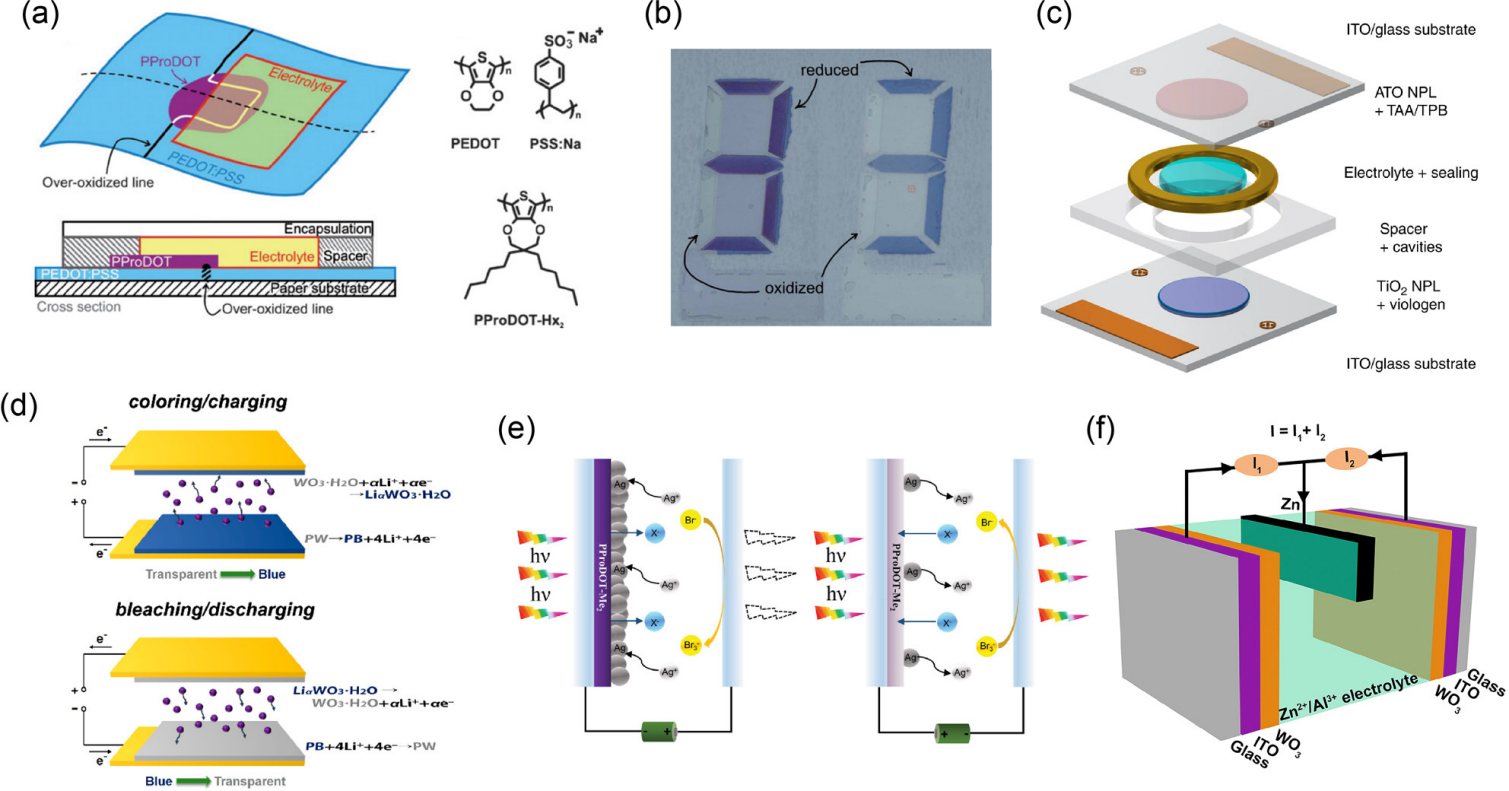
(a) Schematic of PEDOT:PSS-based EC paper with PProDOT-Hx_2_. (b) Photos of PEDOT:PSS-based EC paper with (left) and without (right) PProDOT-Hx_2_. Reproduced from ref (193). Copyright 2009 Royal Society of Chemistry. (c) Schematic of the “transparent-to-black” ECD. Reproduced from ref (195). Copyright 2019 Springer Nature. (d) Schematic of the working mechanisms of the EESD in its coloring/charging (top) and bleaching/discharging (bottom) processes. Reproduced from ref (202). Copyright 2017 American Chemical Society. (e) Schematic of the ECD under negative and positive voltages. Reproduced from ref (207). Copyright 2021 Wiley-VCH GmbH. (f) Schematic of the prototype hybrid Zn^2+^/Al^3+^ EC battery with two WO_3_. Reproduced from ref (214). Copyright 2019 Elsevier Inc.

In practical applications, learning from the cooperative win-win strategy cleverly used in nature is often very helpful for us to solve insurmountable technical bottlenecks, especially when the performance of a single material cannot meet the requirements of higher quality products. Composite materials enable unexpected complementary advantages, and sometimes achieve the effect that “1 + 1 > 2”, similar to the mixing of different metals to form alloys. As a simple and classic example, Magnus Berggren and coauthors used dihexyl-substituted poly(3,4-propylenedioxythiophene) (PProDOT-Hx_2_) as an extra layer to improve the optical contrast of EC paper displays based on PEDOT:PSS (poly(3,4-ethylenedioxythiophene) doped with poly(styrenesulfonate)).^193^ Due to the synchronized electrochromism of PProDOT-Hx_2_ (the high transparency in oxidized state and the intense magenta color in reduced state), the absorption spectrum of PEDOT: PSS in the visible region was effectively broadened (Figure 8a). Based on this result, the authors succeeded in increasing the optical CR by nearly two times without affecting other performances after appropriate thickness optimization (Figure 8b).

This strategy of using composite materials to improve the overall performance has gained wide popularity in research on emerging EC materials and devices.^194^ For instance, the fabrication strategy of complementary color was beneficial to achieve ideal “transparent-to-black” EC switching for future e-papers, according to Egbert Oesterschulze et al.^195^ In related work, the authors introduced two organic complementary EC materials: (i) viologen molecules anchored on the TiO_2_ nanoparticle layer (as a cathodic coloring electrode with color changing from transparent to blue to red) and (ii) tetra-*N*-phenyl-benzidine based on the oxidative dimerization of triarylamine (TAA/TPB) anchored on an Sb-doped SnO_2_ (ATO) nanoparticle layer (as an anodic coloring electrode with color changing from transparent to bronze) (Figure 8c). Due to the synergistic color change of two EC electrodes, the prepared ECD achieved transparent-to-black switching (reaching a minimum transmittance at 605 nm <0.5%). The device also showed a fast switching time (0.5 s) and high CE (440 cm^2^ C^–1^). In addition, related results successfully confirmed the capability of improving EC performance by immobilizing EC molecules on the sintered nanoparticle layer electrode, which has been reported continually in other studies.^196−201^

Based on a similar color complementary strategy, Xiaomin Li and coauthors reported an electrochromic-energy storage device (EESD) combining two inorganic EC materials, Prussian white (PW) film, and WO_3_ nanosheets.^202^ In this work, WO_3_ and PW were coated on two electrodes (Figure 8d). Due to their same color changes (between transparent and blue), the as-prepared device showed improved optical modulation (61.7%), CE (139.4 cm^2^ C^–1^), response speed (1.84 s for coloring and 1.95 s for bleaching), and remarkable cyclic life (Δ*T* decay ratio = 17.5% after 2500 cycles and an almost unchanged capacitance after 1000 cycles). Similar approaches were also reported by Xungang Diao et al.^203,204^ and Chunyang Jia et al.^205^ For example, a NiO/PB composite nanosheet electrode was reported by Chunyang Jia’s group in 2020.^206^ Compared to NiO or PB, the composite electrode exhibited better optical modulation and cyclic life, and a higher charge density.

Another approach that is worthy of attention involves combining metal electrodeposition and organic EC polymers, as reported by Chunye Xu and coauthors.^207^ In this paper, the authors chose poly(3,4-(2,2-dimethyl-propylenedioxy)thiophene) (PProDOT-Me_2_) as EC materials featuring a color change from transparent to blue. The electrodeposition of Ag with a color change from transparent to opaque was introduced into the EC working electrode. By alternating the direction and/or strength of the applied voltages, the ECD they prepared could achieve a pleasant dynamic switch between three states (the transparent, blue, and opaque states) due to tunable reaction processes (Figure 8e). The test results showed that the device achieved an impressive optical modulation (75.7%), cyclic life (>10,000 cycles) and opaque state (near-zero transmittance). This kind of opaque state (an absolute “private” state) is integral to privacy requirements. And there are some other strategies to achieve this state, for example, thermochromically engineered electrolytes based on poly(*N*-isopropylacrylamide) (PNIPAm) hydrogels as reported by Hong Meng’s group.^208^ A similar approach has also been reported by Feng Yan et al. for dual thermo and electro smart windows.^209^ In this work, a copolymer containing *N*-isopropylacrylamide, diallyl-viologen, and 3-butyl-1-vinyl-imidazolium bromide were synthesized and used that served as the thermally responsive, electrically responsive and electrolyte modules. In addition, VO_2_ as another classic thermochromic material can also be introduced for dual thermo and electro smart windows. For example, Ping Jin, Xun Cao, and coauthors combined WO_3_ with VO_2_ as EC materials to achieve this goal.^210^

As far as we know, the combination of metal (Zn) electrodeposition and tranditional EC materials was reported many times by Abdulhakem Y. Elezzabi, Haizeng Li, and coauthors.^211−213^ For example, they reported a novel Zn^2+^/Al^3+^ EC battery prototype in 2019.^214^ In that work, a Zn foil (anode) was sandwiched between two WO_3_ (cathode) materials (Figure 8f). The original state of the prototype (colorless state) had an open-circuit voltage (OCV) of ∼1.15 V and could turn on an LED. After the stored electric energy was depleted, the device changed into a colored state (based on the electrochromism of WO_3_). Moreover, the color depth of the colored state was expected to be enhanced due to the existence of two WO_3_ layers in the optical path.

**Figure 9:**
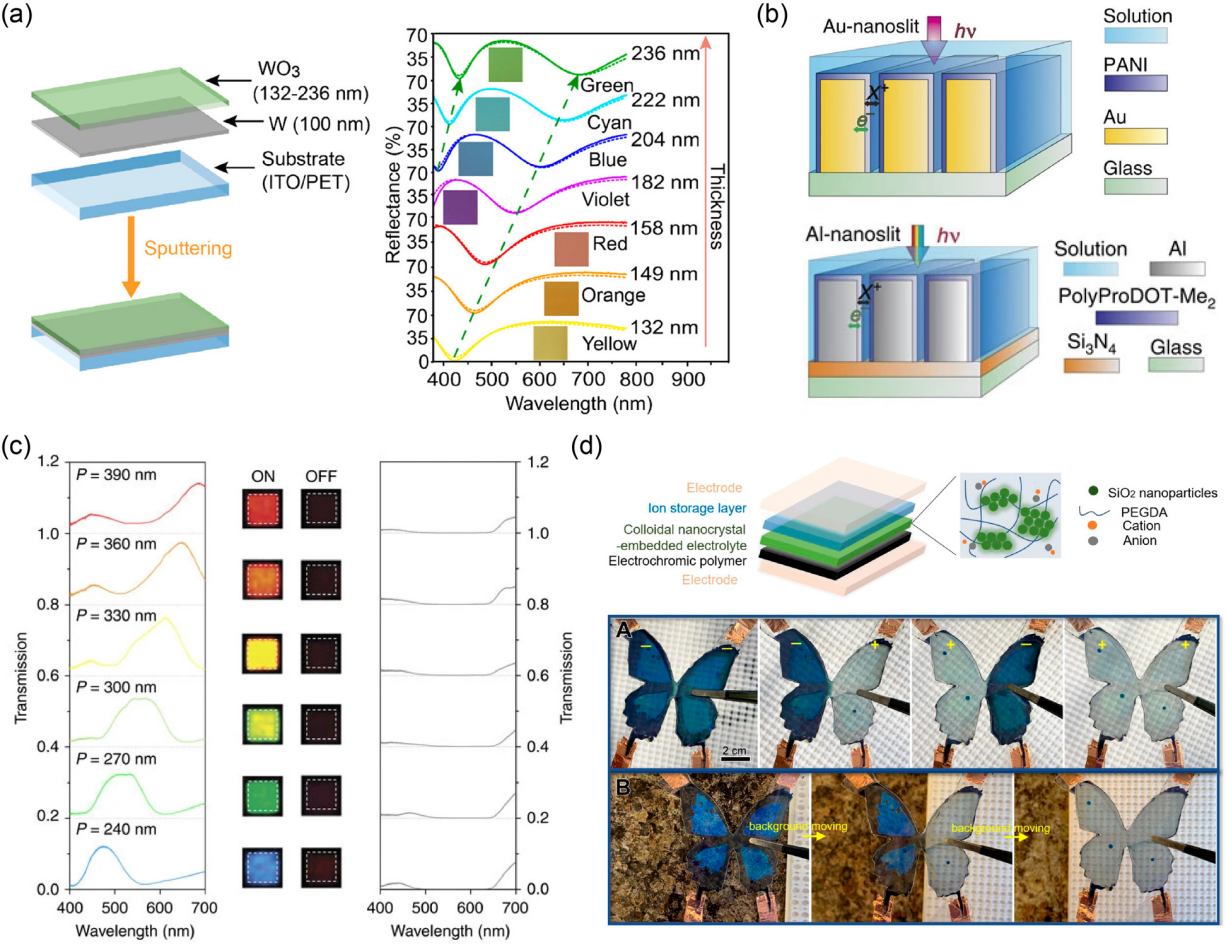
(a) Left: schematic of F–P cavity-type EC electrodes. Right: simulated (dashed line) and measured (solid line) reflection spectra of EC electrodes with different thicknesses of WO_3_ layer. Reproduced from ref (223). Copyright 2020 American Chemical Society. (b) Schematic of a plasmonic EC electrode with Au (top) or Al (bottom) nanoslit arrays. (c) Transmission spectra and photos of EC electrodes based on Al-nanoslit structures with different slit periods (P) in their “ON” and “OFF” states. Reproduced from ref (227). Copyright 2016 Springer Nature. (d) Top: schematic of an ECD with colloidal nanocrystal-embedded electrolytes. Bottom: the color appearance/disappearance of the butterfly device for both active and passive camouflage. Reproduced from ref (228). Copyright 2021 American Chemical Society.

Combining electrically driven chemical-color changes and physical-color (also called structural-color) changes is another strategy worth exploring. Generally speaking, the structural-color change originates from the interaction (refraction, diffuse reflection, diffraction or interference) between light at the appropriate wavelength and the controllable surface microstructure including localized surface plasmon resonances (LSPRs), Mie resonances, and thin-film Fabry–Pérot (F–P) interferences.^215^ Combined with these structural colors, the tunable coverage of chemical-color-change (electrochromism) in the spectrum can be significantly improved. Therefore, different colors can be achieved without changing or modifying the molecular structure of EC materials. In recent years, many attractive studies and results have been reported in this innovative field.^216−222^ For instance, Zhigang Zhao’s group reported an interesting ECD based on F–P cavity-type WO_3_.^223^ In their work, the F–P cavity used was manufactured with a thin layer of metallic tungsten (W) (thickness: 100 nm) between the ITO electrode and WO_3_ EC layer. The introduction of this physical structure induced distinct interference resonances and showed multiple interference peaks and valleys in the 380–780 nm range. That is, light with a relative wavelength was absorbed by the F–P cavity, and the rest was reflected, so the device showed a given color. In addition, the color was tunable by adjusting the thickness of WO_3_ (Figure 9a). Similarly, in the study of organic EC materials, structural colors were introduced to broaden the EC palette.^224−226^ For example, Au and Al metallic nanoslit arrays were fabricated to trigger structural colors, as reported by Ting Xu, Henri J. Lezec, A. Alec Talin, and coauthors (Figure 9b).^227^ Related polyaniline (PANI) and poly(2,2-dimethyl-3,4 propylenedioxythiophene) were selected as EC CPs. These CPs could achieve electrochemical conversion and produced optical modulation under a stimulated voltage. Furthermore, due to the strong structural dependence of structural color changes, the as-prepared devices showed different colors (even full color) by controlling the slit period (*P*) of the nanoslit arrays (Figure 9c).

The aforementioned structural colors are mainly triggered by the special structure in EC layers or electrodes. Introducing related physical structures to electrolytes can also achieve this goal. For example, Jianguo Mei’s research group reported colloidal nanocrystal (containing SiO_2_ nanoparticles) embedded electrolytes in electrochromics.^228^ In their work, the as-prepared electrolytes achieved passive structural-color change according to the background colors (e.g., marble or white backgrounds). The EC polymer was used for the active control of light (color) under electrochemical potential tuning. Therefore, the assembled butterfly device could achieve both active and passive camouflage (Figure 9d).

#### 2.3.3 Introducing Nanostructures

**Figure 10:**
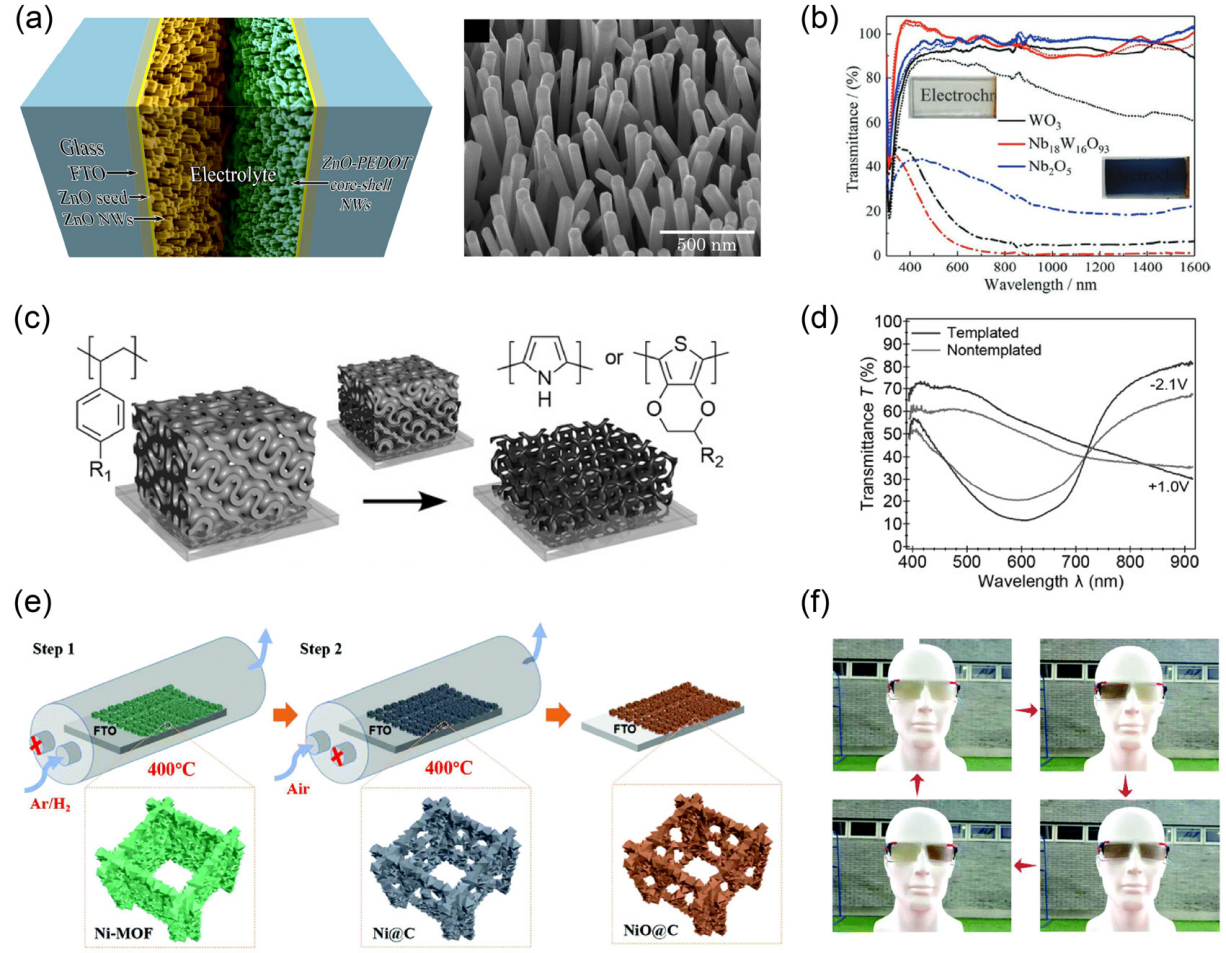
(a) Left: schematic of the nanostructured ECD. Right: field emission scanning electron microscopy image of as-prepared ZnO nanowires. Reproduced from ref (236). Copyright 2015 Elsevier B.V. (b) Transmittance spectra of WO_3_, Nb_18_W_16_O_93_, and Nb_2_O_5_ films in their bleached states and colored states. Inset: photographs of the Nb_18_W_16_O_93_ film in these two states. Reproduced from ref (237). Copyright 2021 Wiley-VCH GmbH. (c) Schematic of the gyroidal styrenic template method for free-standing 3D nanostructured CPs. (d) Optical transmittance spectra of templated and nontemplated EC materials in their colored and bleached states. Reproduced from ref (243). Copyright 2015 Wiley-VCH GmbH. (e) Schematic of the preparation process of the hierarchical porous NiO@C electrode. (f) Photographs of the ECD containing NiO@C electrode for eyewear applications. Reproduced from ref (258). Copyright 2019 Royal Society of Chemistry.

It is well-known that the macroscopic EC reaction rate and the observable color-changing rate depend on the interfacial electron and ion transfer rates, which are proportional to the active surface area. In general, the redox rate of materials on the surface is much faster. Therefore, the introduction of nanostructures is expected to provide a remarkable number of active sites for electron/ion transfer due to its higher specific area, and then further improves EC performance. In recent years, many related studies have been continuously carried out.^229−235^ The commonly used strategies for nano-EC materials involve (i) combining EC materials with nanomaterials and (ii) directly manufacturing EC nanomaterials. For example, a high-performance EC nanotube was reported by Movaffaq Kateb et al.^236^ To fabricate ideal nanotubes, ZnO nanofilms (thickness: 50 nm) were deposited on FTO electrodes through DC magnetron sputtering. Then, the required ZnO nanowire array (diameter: ∼70 nm, length: 600–800 nm) was prepared by the “seed growth” method and hydrothermal synthesis (Figure 10a). Finally, PEDOT (as the EC material) was fabricated on the ZnO nanowire array via in situ electrochemical polymerization. This well-designed EC nanostructure significantly increased the active sites for electron/ion transfer. And then, the calculated diffusion coefficient of Li^+^ to PEDOT was increased to 2.01 × 10^–4^ cm^2^/s, which resulted in a corresponding switching rate of less than 2.2 ms, although the related microscopic electrochemical reaction process and reaction rate were essentially unchanged.

Another classic example of EC nanomaterials is the preparation of nanostructured tungsten-bronze-like Nb_18_W_16_O_93_, as reported by Guofa Cai, Pooi S. Lee and coauthors.^237^ As we know, WO_3_ and Nb_2_O_5_ are two typical inorganic EC materials, and their response rates are limited by ion diffusion within the crystal structures. However, in 2018, Clare P. Grey et al. found that even if the active particles reached micron sizes, niobium tungsten oxides (Nb_16_W_5_O_55_ and Nb_18_W_16_O_93_) still exhibited fast ion-diffusion kinetics due to their appropriate crystal structures.^238^ In fact, this is favorable evidence for using composite materials to improve performance, as discussed above. As reported in this paper,^237^ the authors synthesized Nb_18_W_16_O_93_ nanomaterials through a sol-hydrothermal method. The as-prepared nanomaterials (film) further overcame the limitation of EC performance. Through an electrospray deposition process, a self-supported EC film was efficiently developed. The film demonstrated outstanding EC and energy storage performance, such as a large optical modulation (up to 93% at 633 nm, better than WO_3_ and Nb_2_O_5_) (Figure 10b) and high capacity (151.4 mAh g^–1^ at 2 A g^–1^). This is expected to be used for energy-efficient smart windows in the future.

In addition to the above-mentioned related studies, Ullrich Steiner’s group performed many impressive studies on EC nanomaterials.^239−242^ For instance, they developed some representative free-standing 3D nanostructured CPs (e.g., PEDOT, poly(3,4-ethylenedioxythiophene methanol) (PEDOT-MeOH) and polypyrrole).^243^ These 3D CPs were fabricated with the help of polymeric templates of polystyrene or poly(4-fluorostyrene) (Figure 10c). It is commendable that the related EC performance was indeed significantly improved. For example, the response times of the PEDOT-MeOH-based EC film were reduced by approximately half compared to previous related studies (23 and 14 ms for the coloring and bleaching processes, respectively). The optical modulation also increased (Figure 10d). Meanwhile, because the related manufacturing route did not use traditional corrosive acid etching steps, it was likely suitable for the preparation of various CPs and even inorganic EC materials.^244^ In addition, other nanomaterials and nanostructures related to EC capabilities, such as nanowires,^245,246^ nanofibers,^247^ nanorods,^248^ and quantum dots,^249^ have been extensively studied, and great progress has been made.

Designing EC materials as frameworks with intrinsic porous nanostructures, such as metal–organic frameworks (MOFs),^250,251^ covalent organic frameworks (COFs),^252−255^ and hydrogen-bonded organic frameworks (HOFs),^256^ is also a promising development trend. The frameworks mentioned here are organic or organic–inorganic hybrid crystalline/quasi-crystalline porous materials with a periodic network structure. Due to the structural characteristics (e.g., ultrahigh specific surface areas, adjustable pore structures, high permeabilities of electrolytes for ionic conduction, etc.), they have been widely studied and explored in recent decades. In EC fields, related frameworks usually exhibit remarkable EC performance, especially with regard to response speeds. Recently, Florian Auras, Thomas Bein, and coauthors reported a series of new thienoisoindigo EC COFs with well-designed and synthesized bridge segments.^257^ Among these presented COFs, the best one achieved a high CE of approximately 858 cm^2^ C^–1^ at 880 nm and fast response times (∼0.38 s for the oxidation and ∼0.2 s for the reduction). Although it is still insufficient compared with mainstream light-emitting displays, this result represents one of the fastest switching rates among the known mesoporous EC frameworks to date.

In addition, using these frameworks as nanotemplates to further fabricate EC nanostructures is an interesting idea. A classic example was the novel hierarchical porous NiO@C thin-film electrodes constructed by a MOF template.^258^ Traditional NiO EC materials usually face the problem of slow EC response speed and low CE because of their low electrical conductivity and ionic migration rate due to small lattice spacing. In their work, a Ni-MOF on FTO glass was prepared at first. Then, a NiO@C thin-film electrode was fabricated through two-step pyrolysis of the Ni-MOF (Figure 10e). The results showed that the NiO@C electrode had good ionic migration and electrical conductivity due to the hierarchical porous structure and carbon doping. Furthermore, significantly improved switching speeds (0.46 s for coloring and 0.25 s for bleaching) and good reversibility (90.1% of Δ*T* after 20,000 cycles) were achieved. Finally, a potential application as smart eyewear was demonstrated (Figure 10f).

#### 2.3.4 Designing New Materials

**Figure 11:**
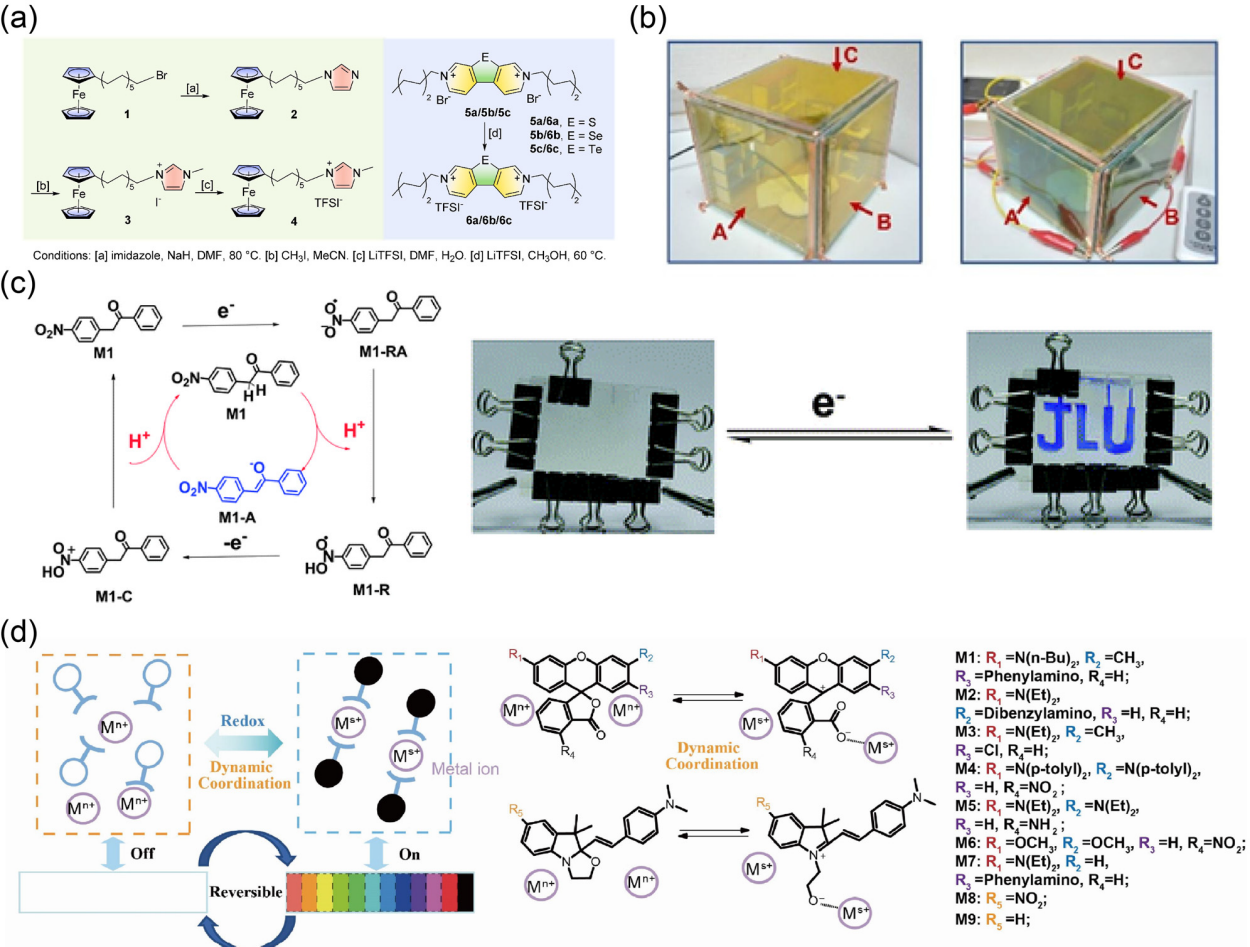
(a) Synthesis of chalcogenoviologen-based ionic liquids and ferrocene-based ionic liquids. (b) A module house with smart windows based on chalcogenoviologen-based ionic liquids in the bleached state (left) and colored state (right). Reproduced from ref (266). Copyright 2021 Elsevier B.V. (c) The EC mechanism of methyl ketone molecule (M1) with the indirect redox mode and corresponding photos before and after electrical stimulation. Reproduced from ref (274). Copyright 2013 Royal Society of Chemistry. (d) Schematic of the design principle of EC materials based on dynamic coordination/dissociation of metal ions and switchable dyes. Reproduced from ref (280). Copyright 2021 Elsevier Inc.

At present, optimizing or designing materials/devices is the most popular approach for improving the overall performance of EC displays. Due to the advantage of the modifiability of organic EC molecules, various new materials have been designed and synthesized. Taking viologen as an example, the introduction of conjugated structures or heteroatoms for special electro-optical properties has been reported many times.^259−263^ For example, a series of chalcogen (S, Se, Te)-bridged viologens (chalcogenoviologens) were synthesized by Gang He’s group.^264,265^ Recently, they reported several EC composites consisting of chalcogenoviologen-based ionic liquids and ferrocene-based ionic liquids (Figure 11a).^266^ These EC composites were used to realize different color switches under an applied voltage bias, and other interesting EC performance including optical modulation (∼70%) and cyclic life (3000 cycles). Furthermore, the authors designed a module house with smart windows prepared by the aforementioned ECDs (Figure 11b), and efficient control of the indoor temperature was achieved. Some other interesting approaches for new materials have also been reported. For example, self-supported semi-interpenetrating polymer networks were studied,^267,268^ and then used in ECDs.^269^ A side-chain modification with polar amide functional groups on EC polymers was introduced to achieve redox switching in aqueous electrolytes.^270^ A layer-by-layer assembly of polyaniline with nanocages was achieved.^271,272^ In addition, EC thin films of perovskite nickelate NdNiO_3_ (NNO) were also prepared, which further increased the selection range of EC materials.^273^

In 2013, Sean X.-A. Zhang’s group reported an indirect EC strategy based on reversible proton transfer induced by electrochemical processes.^274^ In this work, the proton-capture/release ability of “-NO_2_” was dynamically adjusted via its electrochemically driven redox process, defined as “reversible electrobase (or electroacid)”. Thus, the proton of methyl ketone molecules could be captured reversibly according to the redox state of “-NO_2_”, and then relevant color changes were produced (Figure 11c). Subsequently, the authors successively conducted in-depth research and application exploration related to intermolecular and/or intramolecular proton-coupled electron transfer (PCET) (and the reversible electroacid and electrobase method),^275−278^ bond/cation-coupled electron transfer (BCET),^279^ and so on. In 2021, a novel dynamic metal–ligand interaction was reported (Figure 11d).^280^ As shown in that work, by controlling the redox state of metal ions, the coordination and dissociation between metal ions and switchable dyes can be dynamically adjusted to indirectly control the existence or absence of molecular colors. These EC materials with the indirect redox mode effectively avoid the instability problem of the high energy state of dye molecules caused by electrochemically driven redox process. As a result, related EC materials and devices showed significantly better performance and richer material (color) compatibility. Meanwhile, the application potential of some low-end electronic display products was demonstrated also. However, there are still many unresolved problems and technical bottlenecks, for example, the imperfect response speed and photostability. To truly solve these problems and technical challenges, we might need to conduct further in-depth studies to understand the root causes and mechanisms of supramolecular interactions at the molecular, submolecular, molecular aggregate, and atomic group scales.

#### 2.3.5 The Optimization and Innovation of Manufacturing Technologies

Traditional manufacturing technologies for EC materials and devices include vacuum evaporation,^281^ magnetron sputtering,^282^ solution processing,^283,284^ electrochemical deposition,^285^ the sol–gel method,^286^ and so on.^287^ Different processes will affect the performance and manufacturing cost to varying degrees. Therefore, the optimization and innovation in manufacturing technologies are also important for enhancing the commercial potential of future EC displays. In subsequent sections, we will combine with the fabrication of EC displays to discuss related processes and technologies in detail.

## 3 Electrochromic Segmented Displays

To date, there are two mainstream prototypes (segmented displays and pixel displays) for future EC displays, which serve as carriers of the above-mentioned emerging EC materials and devices. In the following sections, we will focus on these prototypes and discuss their underlying scientific issues.

EC segmented display (the first prototype) is a relatively simple display mode; it is used to display some fixed graphics, numbers or letters. This display can also achieve dynamic switching of some contents through simple control of inputted electrical signals. A classic example is the segmented alphanumeric display.^288^ In this kind of display, a point-to-point driving mode based on the one-to-one connection between the electrode lead and display unit is usually adopted. Currently, EC segmented displays have attracted widespread attention due to their remarkable application potential in the aesthetic design of intelligent displays with vivid color and fantastic patterns, although the tunability of displayed information (information capacity) is not as good as that of pixelated displays.

**Figure 12:**
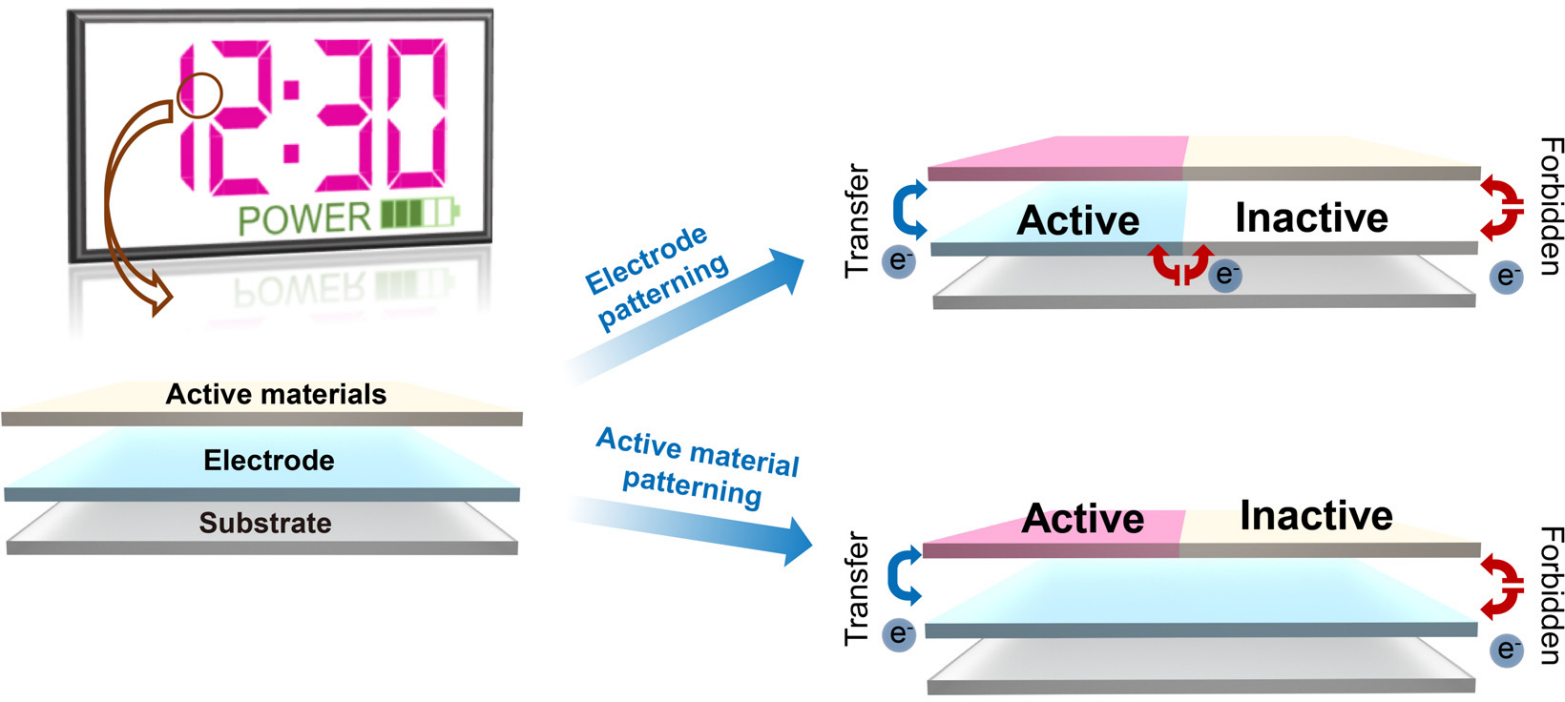
Schematic of EC segmented displays and related fabrication strategies including electrode patterning and active material patterning.

The key point in fabricating EC segmented displays is to prepare EC patterns. According to the working principle of ECDs, several necessary processes must be met: (i) electron transfer on electrodes and at electrode/active material interfaces, (ii) electrochemically driven redox processes of active materials, and (iii) ion transfer inside the device. Under such conditions, the EC activity of a device can be controlled by limiting one or several of these processes. Furthermore, an efficient pattern can be realized by means of local spatial control. As shown in Figure 12, EC patterns can be effectively fabricated through electrode patterning strategy (by limiting the electron transfer) or active material patterning strategy (by limiting the electron transfer and redox process).

### 3.1 Electrode Patterning

Most existing EC materials, except CPs, can be considered as electronic insulators. With this feature, the electron transfer inside the EC layer is naturally limited. Therefore, segmented displays can be realized with the electrode patterning strategy by limiting the interfacial electron transfer between EC materials and electrodes, as shown in Figure 12.

**Figure 13:**
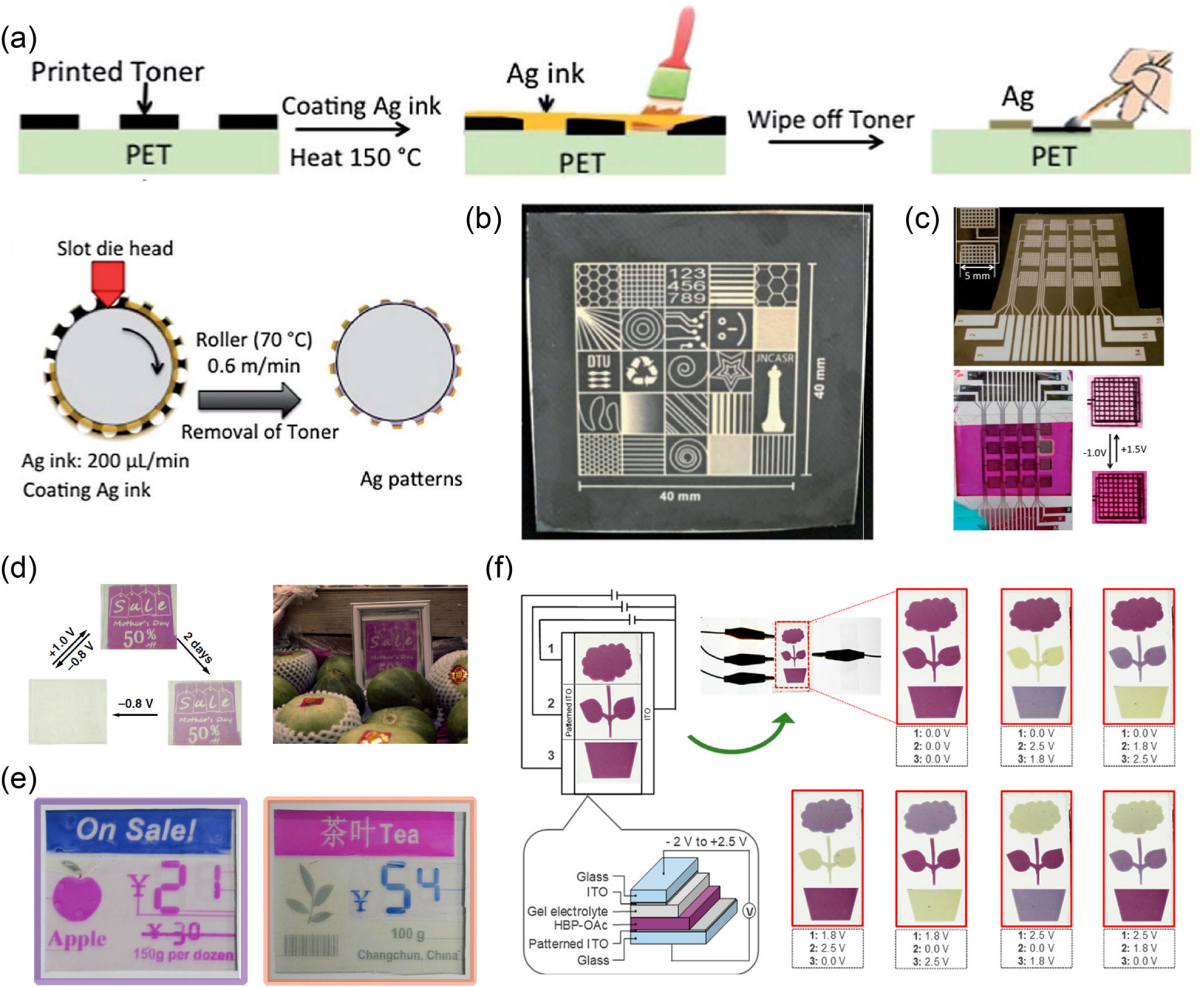
(a) Schematic of the fabrication procedure of patterned Ag electrode. (b) Different Ag patterns on PET. (c) EC display with 4 × 4 pixels based on the pixelated Ag electrode. Reproduced from ref (291). Copyright 2014 Royal Society of Chemistry. (d) Bistable electronic billboards based on EC materials with PCET mechanism. Reproduced from ref (276). Copyright 2019 Springer Nature. (e) Multicolor bistable electronic shelf labels based on EC materials with PCET mechanism. Reproduced from ref (277). Copyright 2019 Springer Nature. (f) The structure and images of EC segmented display with different color information at different voltages. Reproduced from ref (292). Copyright 2020 American Chemical Society.

There are many approaches for fabricating patterned electrodes, such as laser etching,^289^ inkjet printing, and template-assisted coating/printing methods.^290^ As a good verification of the above approaches, Pooi S. Lee’s group fabricated patterned Ag NW electrodes and further demonstrated the first stretchable and wearable patterned WO_3_ ECDs.^120^ The patterned Ag NW electrodes were fabricated based on a polydimethylsiloxane (PDMS) substrate and a prepatterned photomask, which could be used to realize certain required functions even in a 50% stretched state. Another classic example was the sacrificial template method reported by from Frederik C. Krebs and Giridhar U. Kulkarni et al.^291^ In their method, standard toner was digitally printed on a PET substrate with a laser printer. Then, the authors used spin coating, bar coating, or slot-die coating to deposit the Ag precursor ink on the substrate and heated it to remove the solvent of Ag ink. After that, a cotton swab wetted with toluene was used to remove the toner. Based on these, Ag electrode with designed patterns was successfully manufactured (Figure 13a,b). In addition, the authors developed an EC display with 4 × 4 pixels (Figure 13c).

Sean X.-A. Zhang, Yu-Mo Zhang, and coauthors demonstrated various bistable EC segmented displays (e.g., electronic billboards, eye glasses, e-readers, and shelf labels) based on patterned ITO electrodes prepared by laser/chemical etching (Figure 13d,e).^276,277^ In addition, the super energy-saving potential of related display prototypes was discussed in depth. In 2020, Masayoshi Higuchi and coauthors reported an interesting EC “flower” display based on [Fe(II)/Os(II)] supramolecular polymers as EC materials and patterned ITO electrodes.^292^ Three patterns were realized through point-to-point control of different circuits. It is commendable that this display could completely implement the reversible change in different colors under different applied voltages (Figure 13f).

Electrode patterning is a simple and convenient process to quickly obtain the anticipated EC patterned (segmented) displays. This strategy avoids the complicated synthesis or modification in patterning active materials and does not depend on the structures and properties of related materials. Therefore, it exhibits attractive application potential in simple EC devices/displays. On the other hand, its disadvantage is that the resistance of the patterned electrode after etching will increases, especially when the size of the etched patterns is very small.

### 3.2 Active Material Patterning

In the fabrication of EC segmented displays, even pixel displays, the active material patterning strategy is widely used. To achieve this strategy, many advanced processing technologies (e.g., printing and coating, photolithography, etc.) have been reported and applied. Because the size of the required single patterns/pixels varies depending on the application scenario, there is no uniform size standard that can be used as the judging criterion in this field. For instance, the required size of an EC pattern/pixel is on the order of micrometers when the display is used as an electronic screen for mobile phones or monitors, but is on the order of millimeters (even centimeters) when used as a large-area electronic billboard. Therefore, in this review, we will focus on the common technologies for achieving the pattern/pixelation of active materials, but the ultimate resolution of these technologies is not overemphasized.

#### 3.2.1 Printing and Coating

**Figure 14:**
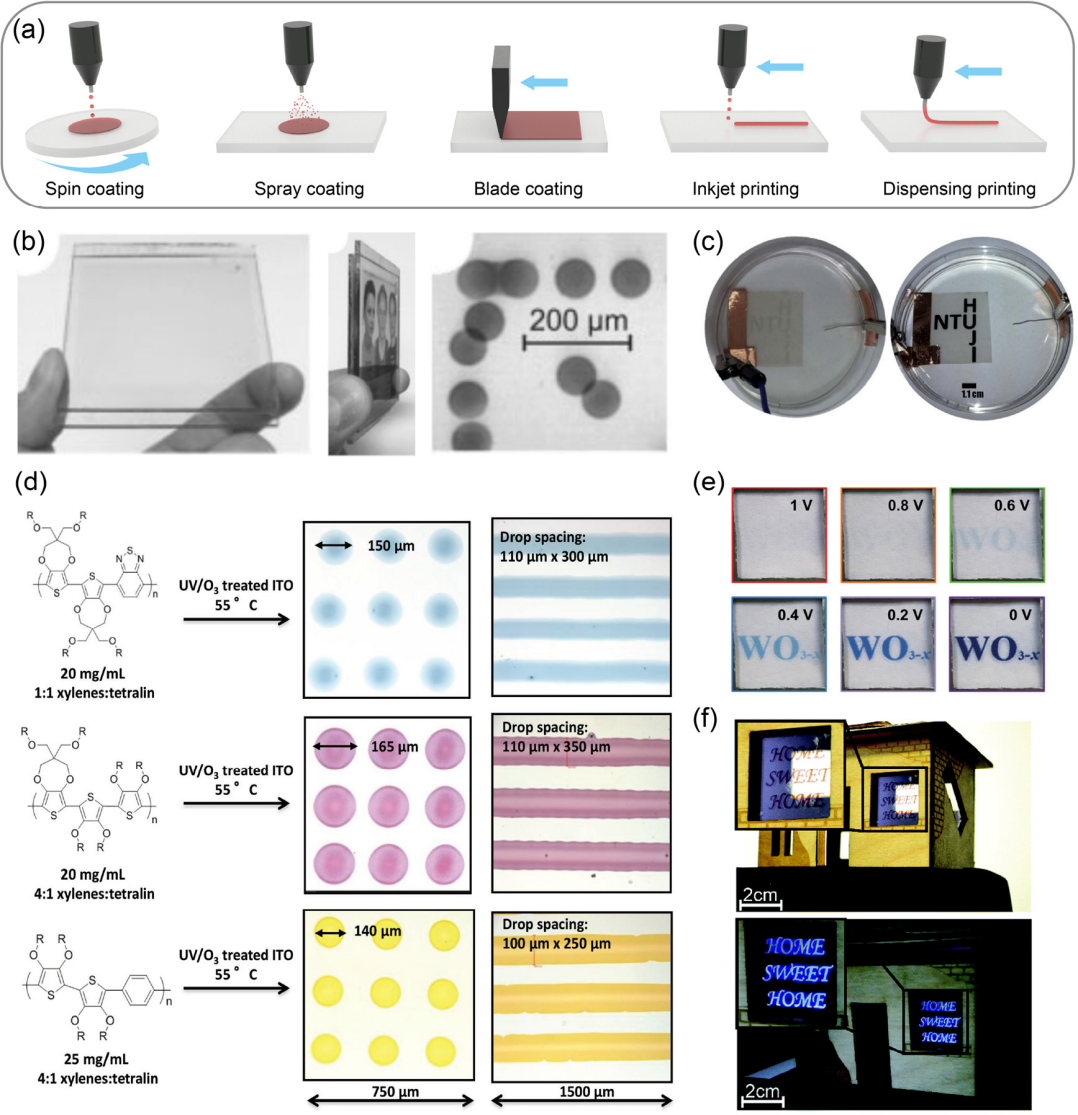
(a) Schematic of printing and coating techniques including spin coating, spray coating, blade coating, inkjet printing, and dispensing printing. (b) Transmissive EC pictures with resolutions up to 360 dpi based on inkjet printing technique and its EC dots. Reproduced from ref (308). Copyright 2004 Wiley-VCH GmbH. (c) EC pixel film manufactured through inkjet printing of WO_3_ nanoparticles. Reproduced from ref (315). Copyright 2014 Royal Society of Chemistry. (d) EC cyan, magenta, and yellow inks for multicolor EC pixels and lines. Reproduced from ref (316). Copyright 2016 Wiley-VCH GmbH. (e) Images of the pseudocapacitive ECD under different electric biases. Reproduced from ref (319). Copyright 2020 Wiley-VCH GmbH. (f) Possible application of EC/EFC dual-mode devices for window advertisements. Reproduced from ref (320) (CC BY 3.0). Copyright 2019 Royal Society of Chemistry.

As typical solution-processing strategies, conventional printing and coating techniques (as shown in Figure 14a) have wide applications in the fabrication of ECDs and in other fields.^293−296^ With these techniques, the strict production environment and expensive equipment required for traditional processing (for example, vacuum evaporation and magnetron sputtering) can be efficiently avoided. Based on the differences in manufacturing processes, printing and coating techniques can be divided into two types: (i) coating techniques and (ii) digital printing techniques. The first type mainly includes blade coating, slot-die coating, spin coating, spray coating, etc. Coating techniques play an important role in roll-to-roll or simple solution-processing for ECDs. Furthermore, some representative EC patterned/segmented display prototypes were fabricated with these techniques.^297−300^ Note that the realization of fine EC patterns is inseparable from additional patterning processes (e.g., printing templates).

Unlike the aforementioned coating techniques, digital printing techniques (such as inkjet printing, dispensing printing, and 3D printing) have the advantage of simple and efficient direct-patterning. These techniques have shown incredible applications in the solution-processing of active materials for EC segmented displays.

Inkjet printing has achieved remarkable progress in the fabrication of printed circuit boards (PCBs), electronic components, solar cells, and other functional materials.^301−304^ With this technology, quantitative droplets of functional inks are ejected out from digital-circuit-controlled nozzle(s) and dropped on substrates to form the desired patterns. In this process, the compatibility of EC inks, printing equipment, and substrates is the key factor. In addition, the relevant material (ink) should maintain the appropriate physical and chemical properties before/during/after printing. The properties described herein include solubility, viscosity, corrosion resistance, electrical resistivity, and compatibility of different components. To date, the inkjet printing technique has shown outstanding application potential in the processing of commonly used EC materials (including small organic molecules, CPs, and even inorganic materials) and related auxiliary materials (such as electrolytes, ion storage materials, elasticizers/flow promoters, and suspending/dispersing agents).^305−307^

In 2004, L. Walder et al. prepared reflective/transmissive high-resolution (360 dpi) EC pictures with an inkjet printing technique (Figure 14b).^308^ As reported in their work, water-dispersible TiO_2_ nanoparticles modified and activated with 1-(2′-phosphonoethyl)-pyridinium bromide derivatives were used as EC inks. After inkjet printing, through the subsequent chemical reactions, a covalently bonded network of viologen polymer/oligomer was formed on the TiO_2_-based electrode. The unwanted thermal diffusion and self-crystallization problems of traditional viologen molecules were cleverly avoided by stable molecular cross-linking and anchoring effect. As a result, an excellent resolution (360 dpi) was achieved.

In recent years, with the help of inkjet printing technology, various related materials and devices have been successfully processed to EC patterned/segmented displays and even pixelated displays.^309−314^ For instance, Pooi S. Lee, Schlomo Magdassi, and coauthors reported that a concentrated dispersion of WO_3_ nanoparticles was printed onto a transparent and flexible silver grid.^315^ The silver electrode formed by self-assembled silver nanoparticles effectively prevented the brittleness of ITO electrodes. Therefore, this electrode was more conducive to the preparation of high-performance flexible electronic devices. After the optimization of the inkjet printing parameters (e.g., the thickness and concentration of WO_3_ nanoparticles), the EC contrast increased up to 72%. Furthermore, the as-prepared EC film showed favorable pixelated potential (Figure 14c). In the research of John R. Reynolds’s group, some impressive multicolor EC pixels and patterns were successfully prepared by the inkjet printing of EC polymer inks (Figure 14d).^316^ These inks could achieve a reversible color change from cyan/magenta/yellow to colorless. After optimizing the solution formulation and compatibility with the substrate, the printed patterns showed a high resolution (size <200 μm). They also cleverly used color mixing theory to further realize multiple secondary colors through multistep printing.

Inkjet printing has also been used to achieve the multifunctional ECDs.^317,318^ For instance, Peihua Yang, Tobias Kraus, Hong J. Fan, and coauthors fabricated flexible pseudocapacitive ECDs.^319^ Additive-free WO_3–*x*_ nanocrystals, carved zinc, and PET-ITO were used as the EC ink, counter electrode material, and flexible electrode, respectively. Through digital inkjet printing, an EC patterned display (with the pattern of “WO_3–*x*_”) was successfully achieved (Figure 14e). The display showed significant color changes between 0 and 1 V and could also be used in pseudocapacitive zinc-ion devices. A relatively high capacity of approximately 60 C g^–1^ at 1 A g^–1^ was obtained. In another report, Carlos Romero-Nieto and Gerardo Hernandez-Sosa et al. prepared an interesting EC/EFC dual-mode display with an inkjet-printed EC polymer.^320^ This dual mode of color and fluorescence ensured that the device could be read comfortably in both daylight and dark conditions for window advertisements (Figure 14f).

**Figure 15:**
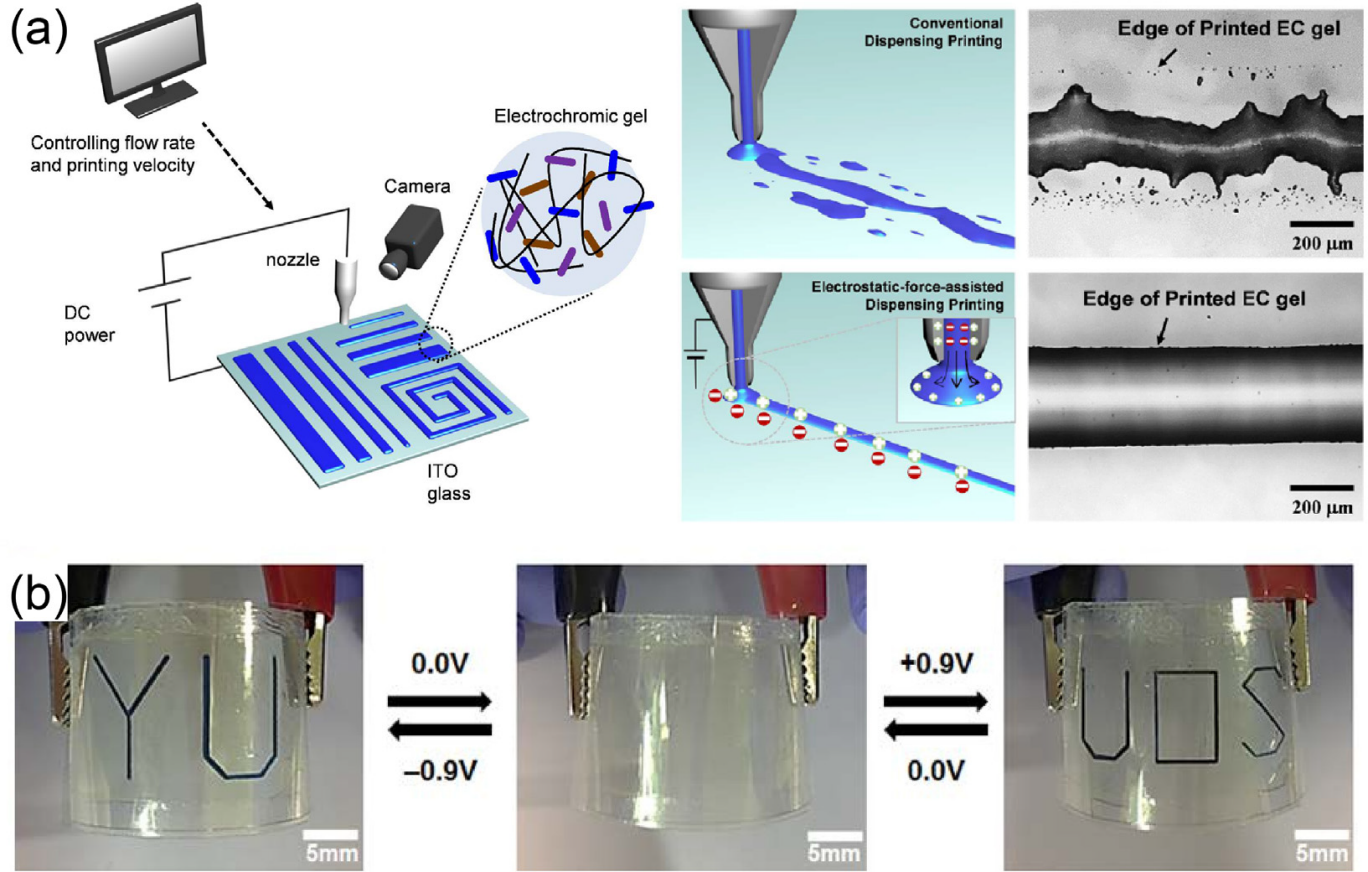
(a) Schematic of EFAD technique and related printing photographs without/with EFAD. Reproduced from ref (322). Copyright 2017 American Chemical Society. (b) Dual images of a WO_3_-based EC display fabricated by EFAD technique. Reproduced from ref (86). Copyright 2020 American Chemical Society.

In fact, in all printing and coating processes (solution-processing) of ECDs, in addition to the compatibility of materials,^321^ the preservation of desired EC characteristics is an important issue. This issue is related to unwanted self-aggregation/crystallization. After the solvent evaporates, the structure and morphology of EC materials may change. Moreover, the interaction between EC materials and the substrate also affects the resolution of the printed pattern. To solve these problems, the printing technology and equipment can be improved or some helpful auxiliaries can be added. A typical example of this is the electrostatic-force-assisted dispensing printing (EFAD) technique demonstrated by Haekyoung Kim, Hong C. Moon and Se H. Kim et al.^322^ This is different from the traditional dispensing printing technique, which uses only compressed air as the power source to drive the material out of the nozzle and adhere to the substrate. In this attractive EFAD technique, an external voltage is applied between the printer nozzle and the substrate (transparent electrode). Thereby, positive charges are distributed on the surface of the printed EC gel, and negative charges are distributed on the substrate. This induced electrostatic interactions between printed EC gel and substrate. Therefore, the adhesion and dimensional stability were efficiently improved. Finally, a clearer printed pattern was obtained, as shown in Figure 15a. The as-prepared EC gel display had an attractive low coloration voltage (∼0.6 V) and large transmittance contrast (∼82% when −0.7 V was applied). Not only organic EC materials but also inorganic EC materials can be processed by the EFAD technique for achieving the desired display patterns. As a typical example, Se H. Kim and Hong C. Moon et al. printed WO_3_ on an ITO-coated electrode in 2020.^86^ As reported in this work, dimethyl ferrocene dissolved in the ion gel was used as the anode material. By printing different WO_3_ patterns on two ITO electrodes, the authors successfully developed an interesting dual-image display prototype (Figure 15b).

**Figure 16:**
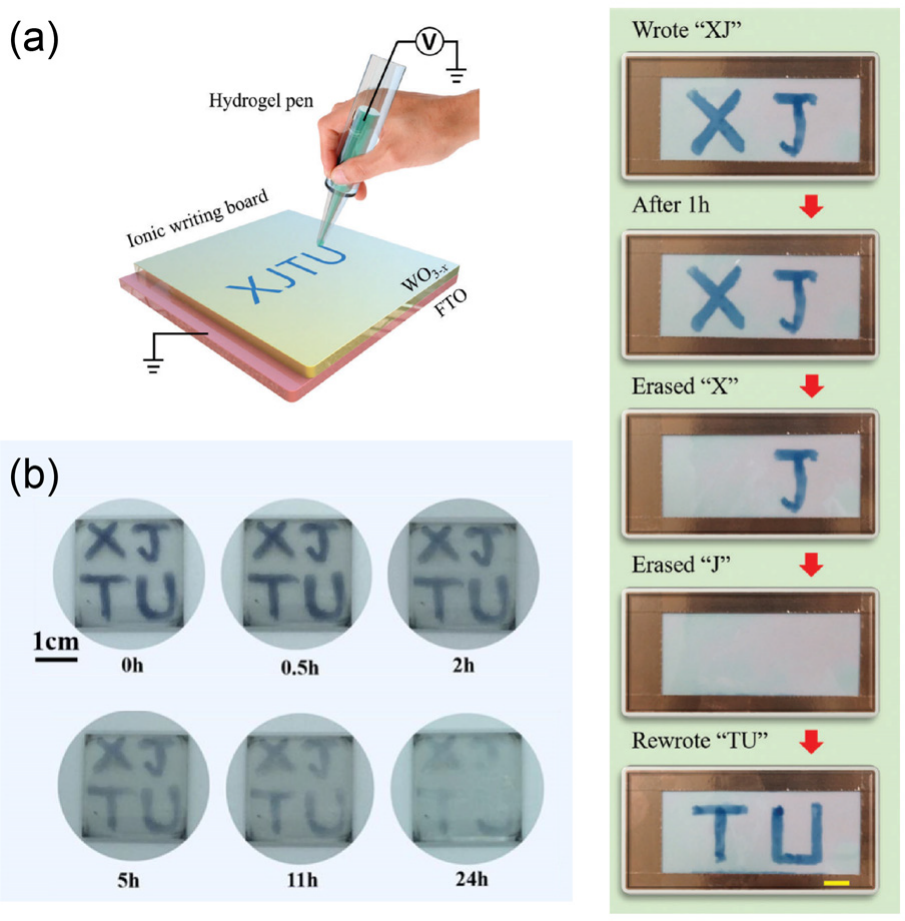
(a) Schematic of the erasable and rewritable ionic writing board and its displaying and erasing photos. Scale bar: 1 cm. Reproduced from ref (323). Copyright 2018 Royal Society of Chemistry. (b) Photos of an EC pattern (colored state) after aging for different times. Reproduced from ref (38). Copyright 2021 American Chemical Society.

EC patterns achieved by handwriting can also be considered derivatives of printing techniques. As a classic example, Hong Wang, Huajing Fang, and coauthors reported a novel WO_3–*x*_ EC hydrogel writing board (Figure 16a).^323^ In their demonstrated prototype device, only the area where the hydrogel pen was in contact with the EC board underwent ion transfer between WO_3–*x*_ and the hydrogel, thereby making color changing possible. The as-prepared device had a large transmittance modulation up to 70%. This construction idea of ionic writing boards cleverly takes advantage of the good stability of inorganic EC materials and the fast ion transfer speed of hydrogels. Then, the authors further prepared MoO_3–*y*_/WO_3–*x*_ films as EC materials based on MoO_3–*y*_ nanosheets.^38^ Through an integrated Al anode, an interesting self-powered ionic writing board was finally achieved (Figure 16b). In 2020, Gang He and coauthors used a similar fabrication method to realize another gel-based writing board with viologen-derivative-based EC materials.^324^

#### 3.2.2 Template-Assisted Method

Apart from the aforementioned printing and coating techniques, the template-assisted method is also a widely used approach for fabricating patterned active materials. With this method, related active materials can form zero/one/two/three-dimensional patterns transferred from templates. For example, in traditional preparation processes, such as vapor deposition, electrodeposition, magnetron sputtering and solution processing, patterned EC materials can be prepared with patterned templates.^325,326^ Currently, various promising templates such as photoresist templates, PDMS templates, oxidant templates, and fine metal masks,^327−330^ have been developed. Moreover, nanostructured templates have enabled the transfer of nanopatterns and nanostructures to EC materials as nanoimprint technologies.^331^

**Figure 17:**
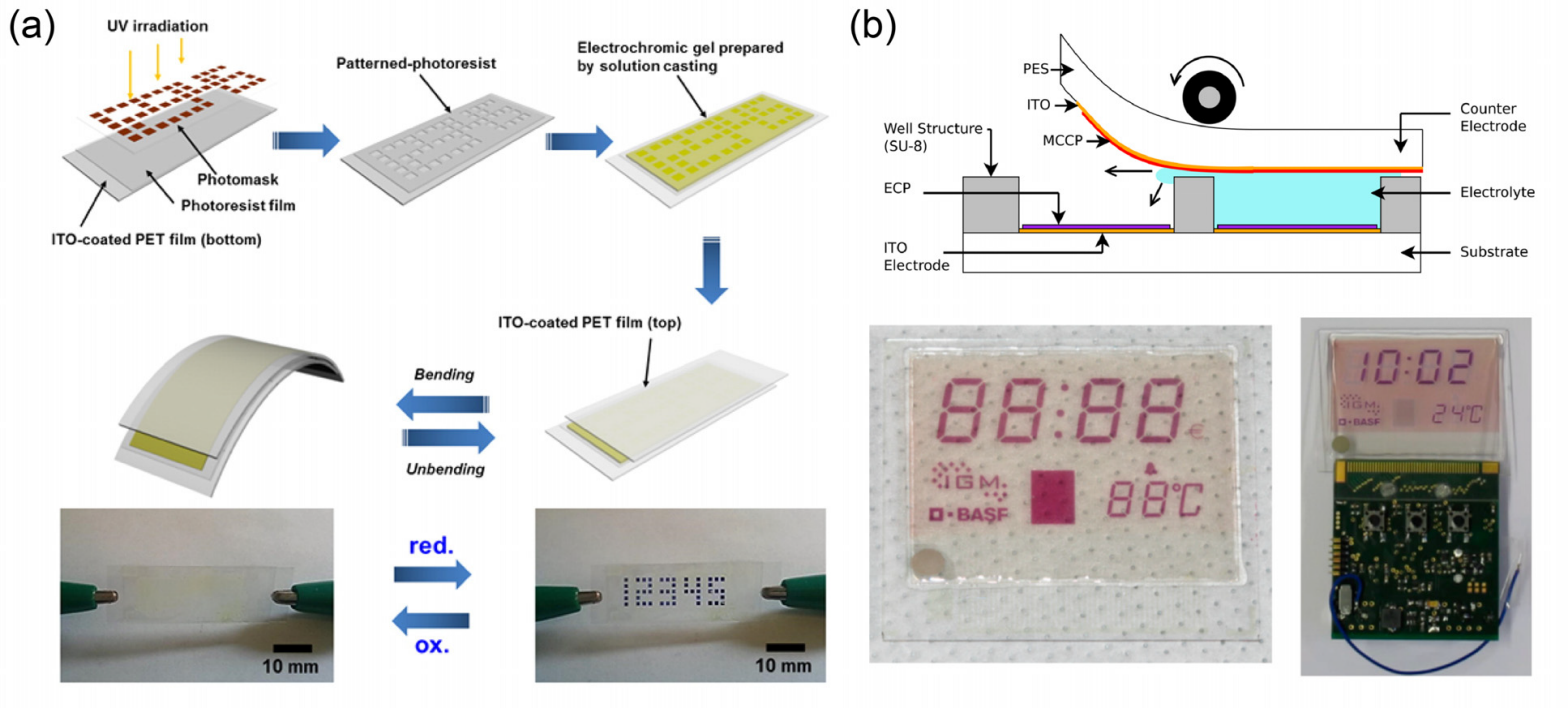
(a) Fabrication of organic ion-gel EC displays based on viologens and corresponding photos. Reproduced from ref (336). Copyright 2015 American Chemical Society. (b) Photoresist-assisted fabrication of patterned EC polymer and their application as an EC clock prototype. Reproduced from ref (338). Copyright 2015 American Chemical Society.

Herein, we briefly introduce the template-assisted method taking photoresist as an example. Photoresists are a class of organic materials with photopolymerizable/degradable functions; they can form representative templates and have been widely used for the fabrication of electronic products, for example, PCBs and microelectronics.^332−335^ Photoresists are also very compatible with most existing EC materials for manufacturing related patterns. For instance, Timothy P. Lodge and C. Daniel Frisbie et al. fabricated interesting organic ion-gel EC displays with commercial negative photoresist (SU-8) templates (Figure 17a).^336^ The ion gels were composed of methyl viologen (as EC molecules), ferrocene (Fc) (as anodic species), copolymers, and ionic liquid. With the help of SU-8 templates, the size of as-prepared pixel could reach 1 × 1 mm^2^. In a follow-up study, flexible three-color (red, green, and blue) EC ion-gel displays were also fabricated via the “cut and paste” strategy.^337^

Another example of a typical EC segmented display was reported by Julian Remmele et al.^338^ The display was successfully prepared through solution-based roll-to-roll process, spray coating, and photocuring with the help of commercial SU-8 and epoxy-based negative photoresist in an ambient atmosphere (Figure 17b). By combining the display with integrated PCBs, the authors successfully demonstrated an interesting prototype of EC clock with switching cycles exceeding 100,000. This prototype has good application prospects in the field of energy-saving displays due to its bistability, where no additional energy is required for maintaining the displayed information.

#### 3.2.3 Photolithography

**Figure 18:**
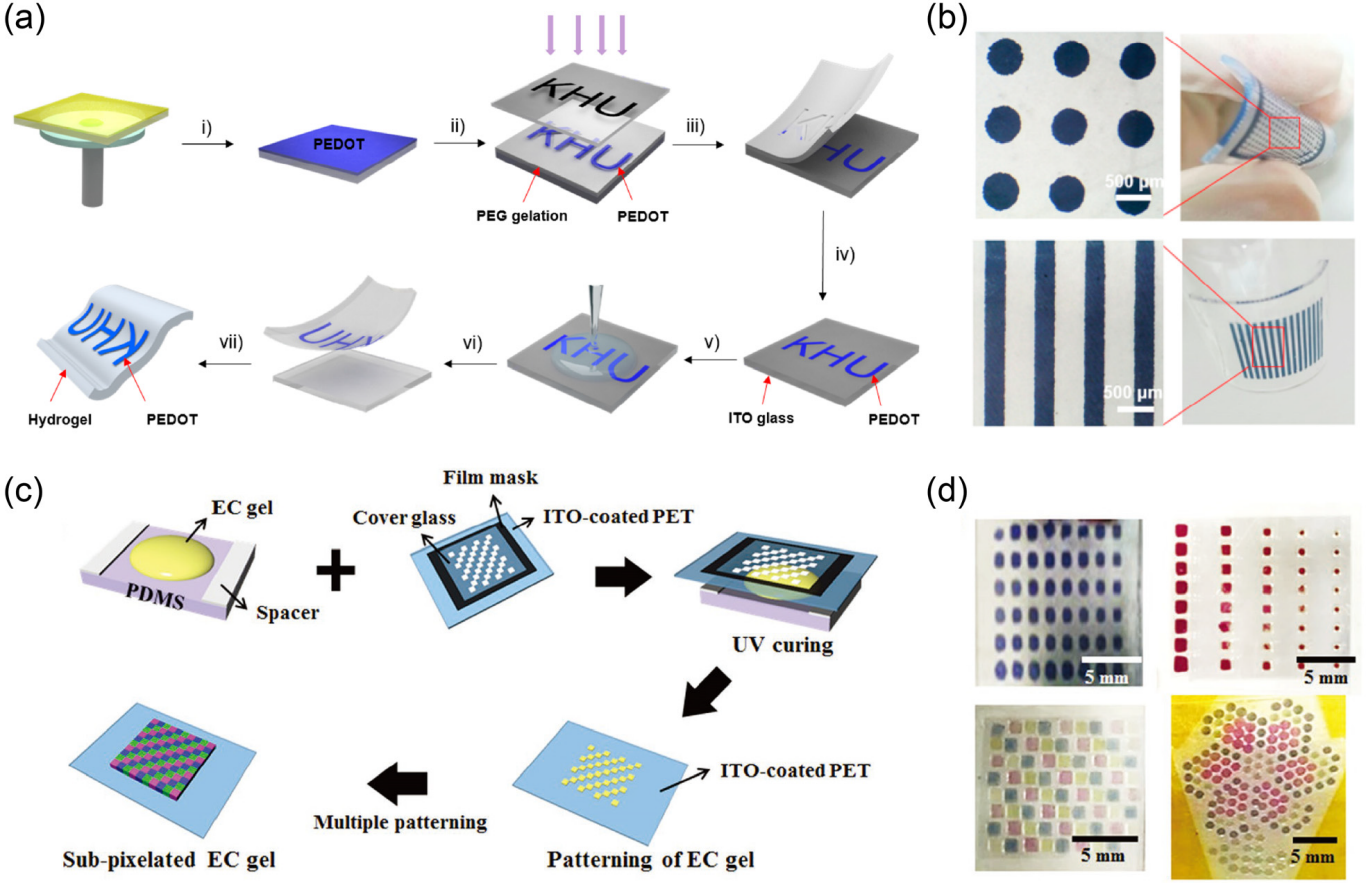
(a) Fabrication of PEDOT-based EC displays through UV-induced PEG photolithography and (b) related photos. Reproduced from ref (340). Copyright 2016 American Chemical Society. (c) Manufacturing process of multicolor EC subpixelated ion gels and (d) related photos. Reproduced from ref (341). Copyright 2019 Wiley-VCH GmbH.

The traditional template-assisted method is relatively complicated because it usually requires a multistep postprocessing stage. Inspired by the excellent photoresponsive ability of commercial photoresists in the semiconductor industry, direct photolithography strategy was proposed and developed by outstanding researchers in related fields.^339^ For instance, Jung H. Kim and Jungmok You et al. reported this novel strategy for preparing patterned PEDOT through UV-induced poly(ethylene glycol) (PEG) photolithography (Figure 18a).^340^ In this fabrication process, the PEG precursor solution was coated on a PEDOT-based EC film prepared by oxidative polymerization. Through the UV photolithography of PEG with a photomask for a patterned hydrogel, the PEDOT film could be peeled away with the PEG hydrogel in the UV-exposed region due to the strong adhesion, while the unexposed area remained. Based on this, high-resolution PEDOT patterns/pixels can be achieved (Figure 18b). Furthermore, patterned/pixelated PEDOT could be easily transferred to flexible substrates for flexible devices.

Jae-Min Myoung et al. introduced a photopolymerized EC gel (Figure 18c).^341^ The EC gel contained poly(ethylene glycol) diacrylate, 2-hydroxy-2-methylpropiophenone, EC molecules (monoheptyl-viologen for magenta, diheptyl-viologen for blue, and the composite of diheptylviologen and diphenyl-viologen for green), and ionic liquid. PET-ITO was used as the flexible electrode. Through UV photolithography with a photomask, subpixelated EC gels with high-resolution (a minimum size of 200 μm) and high-durability (mechanical bending for 1,000 cycles at a bending radius of 10 mm) were successfully prepared (Figure 18d). Furthermore, the authors introduced single-walled carbon nanotube/Pd-coated Ag NW bilayer electrodes (SWCNT/PCSN) to replace the aforementioned PET-ITO electrodes and further improved the corresponding mechanical properties.^342^ The results showed that the as-prepared display could maintain desired stability after 500 cycles of repeated rollable tests (radius of curvature = 2.5 mm). In 2019, a similar method was fully demonstrated in “the photosensitive sol–gel method” for microscopic fine-pattern reported by Yang Ren, Zongfan Duan, and coauthors.^343^

**Figure 19:**
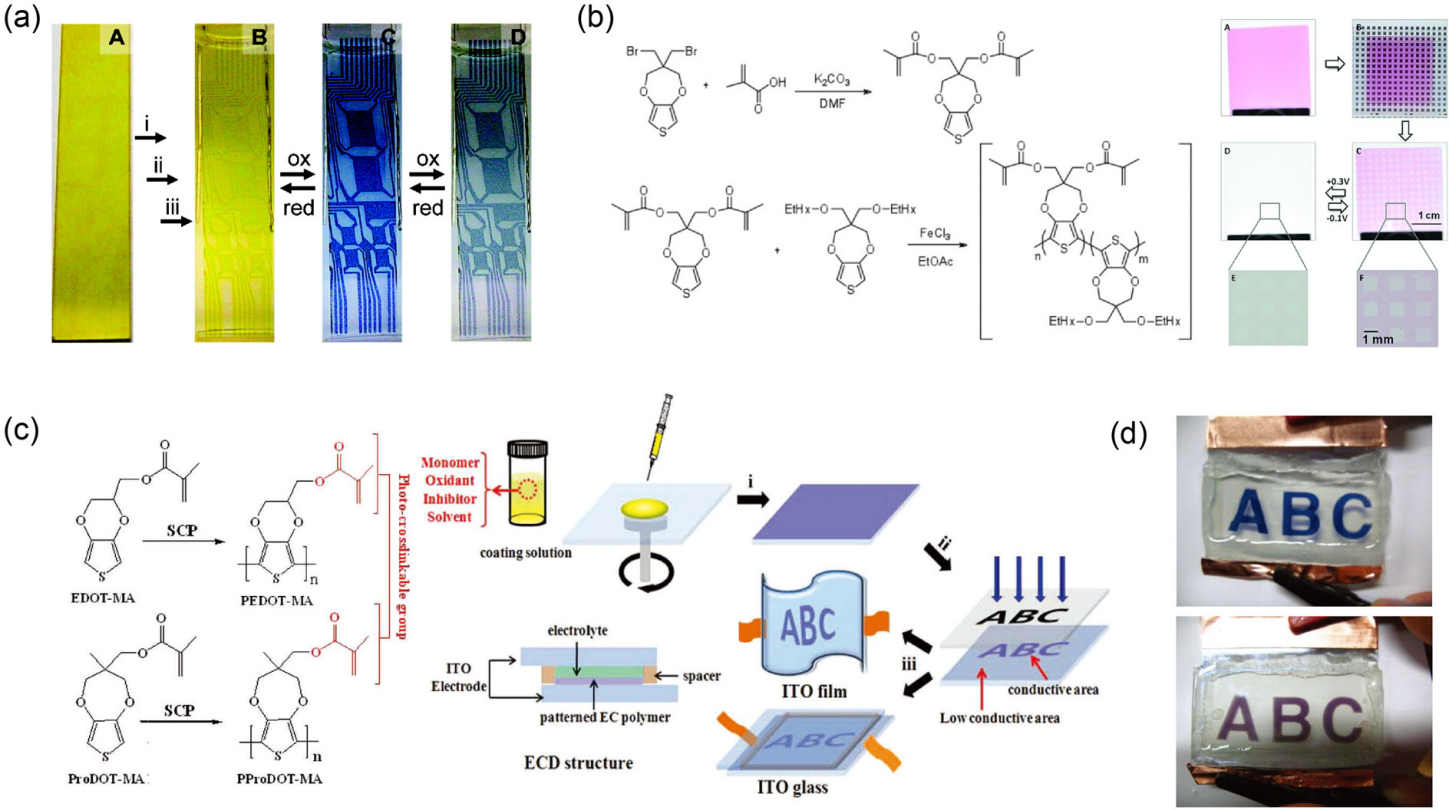
(a) The first oligomer-based photopatterned ECD. Reproduced from ref (344). Copyright 2008 American Chemical Society. (b) Synthetic route of photoresponsive polythiophene derivative molecules and millimeter-scale EC pixels prepared through direct photolithography. Reproduced from ref (345). Copyright 2013 Wiley-VCH GmbH. (c) Manufacturing process of photoresponsive polythiophene derivative EC displays. (d) The as-prepared ECDs composed of PEDOT-MA (top) and PProDOT-MA (bottom). Reproduced from ref (346). Copyright 2011 Wiley-VCH GmbH.

Different from the aforementioned approach of doping EC materials into photolithographic materials (the doping mode), modifying EC materials with photoresponsive functional units for direct photolithography (the modifying mode) is another classic strategy. In 2008, the team of John R. Reynolds, a pioneer in the field of EC polymers, reported the first photopatterned ECD based on oligomers.^344^ As reported in their work, acrylate functional groups were cleverly attached to the oligomers, enabling them to be further cross-linked. Due to the good solubility of the oligomers in common organic solvents, they easily form thin homogeneous films. Furthermore, a photopatterned ECD was successfully constructed after cross-linking EC materials in the exposed area and then dissolving/removing EC materials in the unexposed area (Figure 19a). The cross-linked films exhibited a noticeable color change (from bright yellow to clear blue, then to a transparent state with a small residual gray color) and relatively good reversibility (at least 400 cycles). Furthermore, in 2013, Frederik C. Krebs and John R. Reynolds et al. reported the direct photolithography exploration of EC conjugated copolymers (Figure 19b).^345^ In their work, the copolymers were composed of an alkoxy-substituted propylenedioxythiophene (ProDOT) and an acrylate-substituted ProDOT. The copolymers could be cross-linked after exposure to UV light (350 nm), and millimeter-scale EC pixels were successfully achieved by in situ direct photolithography.

Another approach for direct photolithography is to reduce the activity of EC materials by over cross-linking. Based on this idea, Eunkyoung Kim et al. synthesized two polythiophene derivatives with methacrylate functional groups (PEDOT-MA and PProDOT-MA) (Figure 19c).^346^ The colors of these two polymers could change from blue/purple to colorless under electrical stimulation. They were spin-coated onto ITO glass and subjected to UV exposure with a photomask. Due to the highly cross-linked structure of the side chains, the conductivity of EC materials in the exposed area dropped drastically (above 99.99%), and the original colors was photobleached. On the other hand, EC materials in the unexposed area maintained good EC properties. Thus, the expected patterned (flexible) display was finally realized (Figure 19d). This approach effectively avoids postprocessing operations and reduces the difficulty/cost of related industrial processing. However, a potential problem is that there are a large amount of methacrylate residues in the EC region, which might also negatively affect EC performance and photostability. Determining how to solve this negative impact is an issue that should be extensively considered in the future.

The modifying strategy is usually based on in situ polymerization and cross-linking of EC molecules/materials. Some factors including the degree of aggregation, the approach of structure cross-linking and the adhesion to substrates, are closely related to performance. For example, excessively dense cross-linking often leads to poor adjustability of structures and poor electrolyte permeability, thereby weakening the electrical conductivity and EC efficiency.

Compared to the doping mode, the modifying mode can more efficiently avoid the harmful thermal diffusion of EC molecules and the potential loss of material involved during postprocessing (for example, the development process after photolithography). Therefore, a higher visual resolution can often be achieved. However, the modifying mode has high requirements for the rational design of molecular structures. Therefore, complex chemical synthesis may also be involved sometimes; this undoubtedly increases the preproduction cost.

#### 3.2.4 Other Advanced Patterning Methods

Active material patterning can also be prepared through other advanced methods. For instance, Hongzhi Wang, Gang Wang, and coauthors used laser etching to fabricate a desired EC segmented display when studying sodium ion (Na^+^)-based electrochemical systems with MOFs as ion-transport channels.^347^ A pulsed CO_2_ laser was used for maskless laser writing. This processing technique showed a great patterned/pixelated effect and application value in consumer electronics and Internet-of-Things interactions. In addition, other methods such as maskless electron beam etching/curing,^348,349^ horizontal redistribution of ions,^350^ hydrophilic/hydrophobic differences,^351^ and direct thermal patterning,^352,353^ were successfully introduced for the fabrication of EC patterned/segmented displays. All of the technologies (methods) mentioned here have their own advantages and disadvantages. Although they are worthy of further in-depth exploration and application expansion, since the relevant researches are not extensive enough, we will not introduce them in detail here.

## 4 Electrochromic Pixel Displays

**Figure 20:**
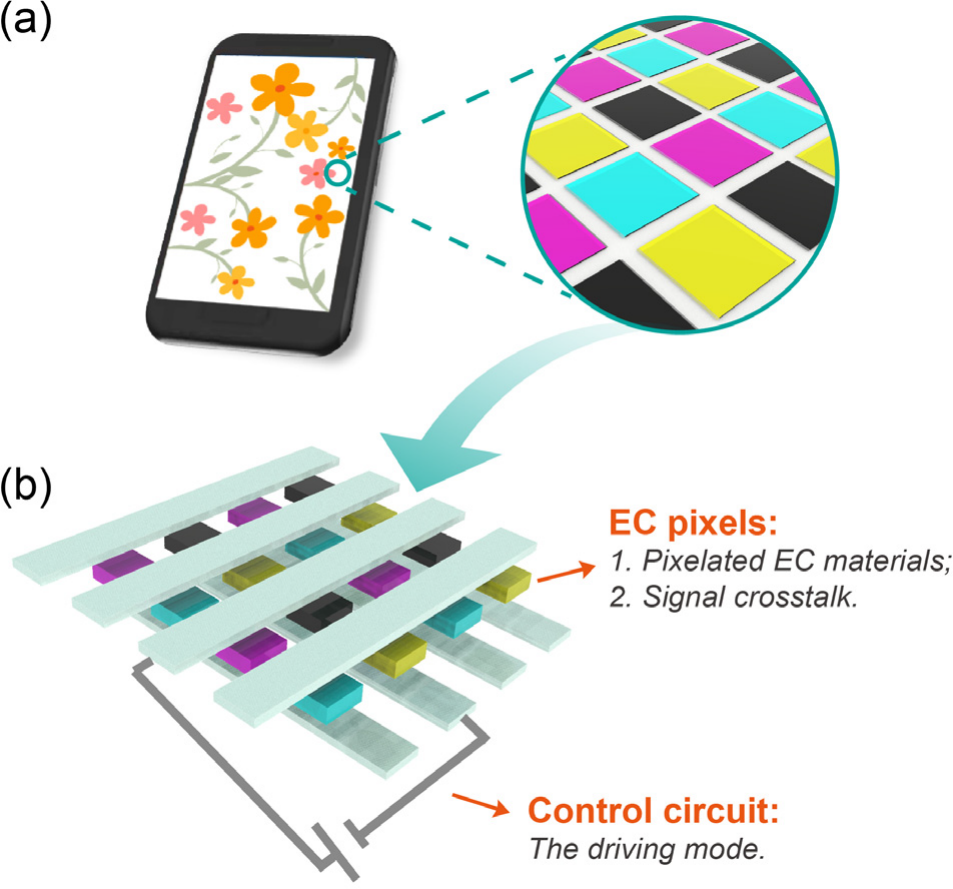
(a) An EC pixel display applied to future smartphone screens based on EC subpixels with CMYK printing colors. (b) The structure of EC pixel displays (composed of electrodes, EC pixels, and external circuits) and related key issues in the fabrication process.

As another useful and important application prototype for future EC displays, EC pixel displays composed of EC smart pixels have powerful information display capabilities. Such displays are expected to be applied to complex human–computer interactions, such as in smartphone screens (Figure 20a). By controlling the “on/off” state and ratio of smart pixels, the displays can realize an arbitrary switching and dynamic adjustment of the displayed content and information. Thus far, building an ideal EC display based on individually addressable EC smart pixels is still a formidable challenge. Moreover, the corresponding applications have much higher requirements for the coordination of whole working systems including EC pixels, electrodes and external circuits, as shown in Figure 20b. That is, pixelated EC materials need to be successfully fabricated and rationally arranged; the electrodes need to match EC materials. In addition, the device requires precise control circuits and suitable driving modes to ensure accurate electrical signal input. Unfortunately, the image diffusion and signal crosstalk problems occur frequently, which affect the displayed image resolution. Determining how to prevent these annoying problems is an urgent topic. To facilitate the understanding, we will systemically discuss these key issues one by one in the following sections.

### 4.1 Pixelated Electrochromic Materials

To realize the pixelation of EC materials, two main factors (the pixelated approach and the arrangement of EC pixels/subpixels) need to be considered. Fortunately, there are many encouraging approaches that can be used to achieve high-resolution EC pixels/subpixels. Related processes and methods are almost the same as the aforementioned approaches for patterned EC materials and devices. Since they have been discussed, we will focus on the arrangement of EC pixels (subpixels) here.

Generally, an EC pixel consists of one or several EC subpixels. Thus, by controlling the “on/off” ratio of different subpixels, multiple secondary colors (and even full colors) can be realized. Due to the typical absorption mode (subtractive color mode) of EC materials, developing corresponding subpixels with cyan, magenta, yellow, and black (CMYK)-based primary colors is considered a suitable approach. However, achieving high-performance CMYK-based EC subpixels does not appear to be easy. In this process, any available color with suitable usage scenarios is welcomed. Currently, with the in-depth research of EC materials, multiple vivid and plentiful colors have been realized, which provides the color foundation for future multicolor EC subpixels. For organic EC materials, their color tuning mainly depends on structural modification.^354,355^ For example, the colors of viologens in reduced states are dramatically affected by the interrelated substituent group, conjugated degree, and even counteranions.^356−360^ Similarly, the side chain functionalization approach is also widely used for realizing the color tuning of CPs.^361,362^ Furthermore, structural color is usually introduced to broaden the EC palette.^363,364^ This approach has been discussed systematically in Section 2.3.2.

**Figure 21:**
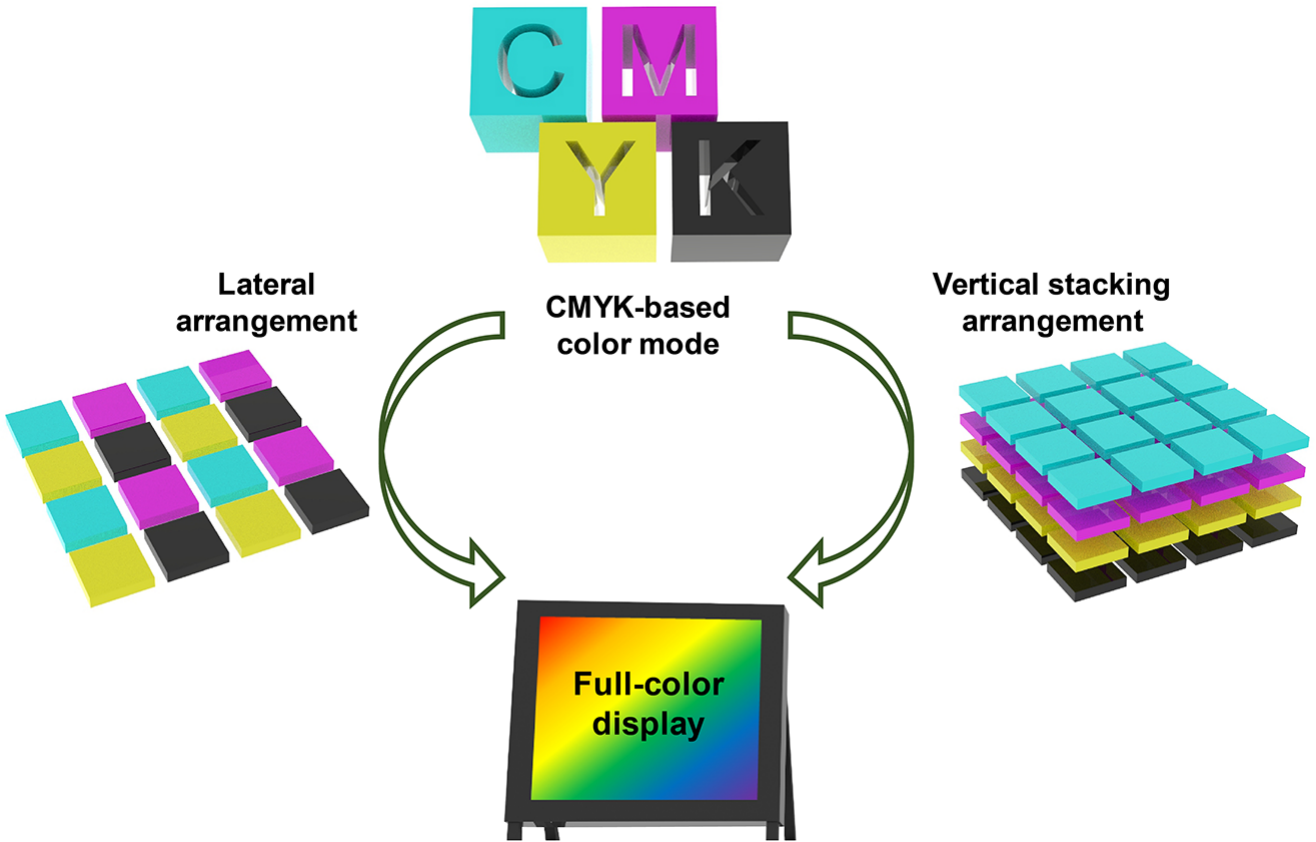
Potential EC full-color displays fabricated by the lateral (horizontal) arrangement or vertical stacking arrangement of EC subpixels with a CMYK-based primary color mode.

Determining how to design the device structure and make colorful EC subpixels have no interference with each other,^365,366^ is another key issue in the study of pixelated EC materials. There are usually two modes for achieving an orderly stacking of subpixels: lateral (horizontal) arrangement and vertical stacking arrangement (Figure 21).

#### 4.1.1 Lateral Arrangement

**Figure 22:**
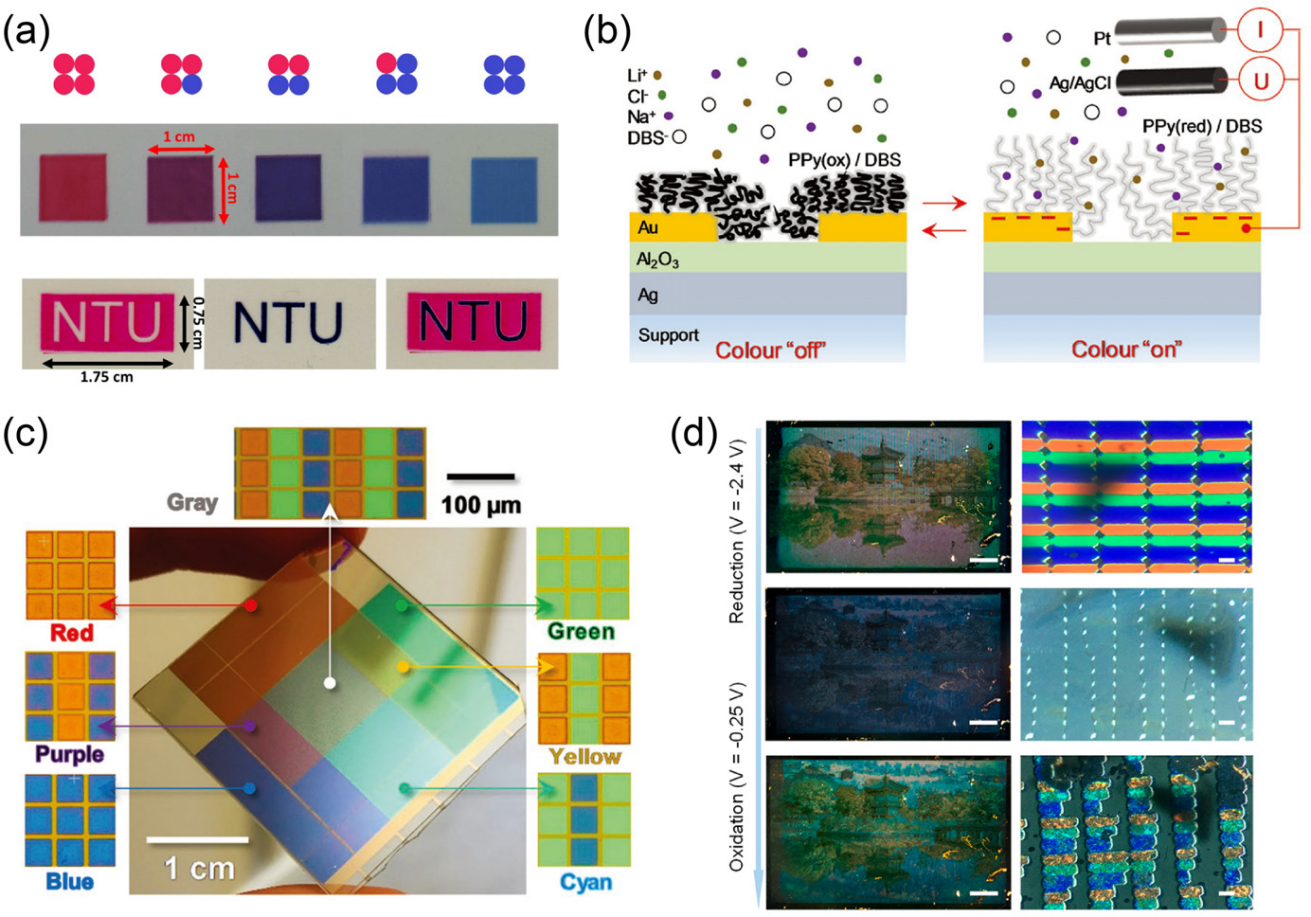
(a) Multicolor EC films with EC subpixels based on metallo-supramolecular polymers digitally controlled by the print doses of each species. Reproduced from ref (368). Copyright 2015 American Chemical Society. (b) The EC hybrid system combined with CPs and PMSs. (c) The multicolor ECD based on RGB subpixels. Reproduced from ref (371). Copyright 2016 Wiley-VCH GmbH. (d) Photos of transmission-type EC displays during electrochemically driven redox processes and corresponding microscopic images. Reproduced from ref (372). Copyright 2020 American Chemical Society.

To date, the lateral (horizontal) arrangement of subpixels is the most common arrangement mode for full-color displays. All existing light-emitting displays (e.g., LCDs, LEDs, and OLEDs) rely on the lateral arrangement of their red, green, and blue (RGB) subpixels.^367^ EC materials can also be processed into multicolor (even full-color) displays with this mode. For instance, Masayoshi Higuchi, Kuo-Chuan Ho, and Ying-Chih Liao et al. reported a multicolor ECD based on the lateral arrangement of EC subpixels.^368^ In this report, the diameter of each EC subpixel was 100 μm. To fabricate the subpixels, two types of metallo-supramolecular polymer solutions with different primary colors were coated through an inkjet printing technique (Figure 22a). The color of the as-prepared device could be adjusted by changing the ratio of EC subpixels, which was digitally controlled by the print doses of each species. The result of realizing a tunable multicolor by combining different subpixels verified the feasibility of preparing EC full-color displays with the lateral arrangement mode.

In fact, multicolor EC subpixels can be prepared not only by EC materials with different colors, but also by tunable structural colors, as discussed in Section 2.3.2 above. Based on this approach, colorful pixels (subpixels) can be easily achieved, which dramatically simplifies the difficulty of color/material design.^369,370^ For example, Andreas B. Dahlin’s group reported an interesting hybrid material combining CPs and plasmonic metasurfaces (PMSs), and further realized a multicolor electronic paper.^371^ The PMSs were composed of a 150 nm silver film (for providing a high reflection basis), an alumina spacer layer (for tuning the reflective color by F–P interference), and a gold film with short-range ordered nanoholes (Figure 22b). The gold film used could provide strong resonant scattering. Furthermore, polypyrrole was coated on PMSs to achieve a dynamic color change. Finally, the authors used the photoresist (Microposit S1813) to successfully fabricate 50 μm pixels with a resolution approximately equal to that of the human eye. Through the lateral arrangement of RGB subpixels, multiple secondary colors (such as yellow, purple, and cyan) were achieved (Figure 22c). Due to the different colors of polypyrrole in its oxidation state and neutral state, the as-prepared electronic paper realized multicolor switching before and after the voltage bias. As another classic example, Taek D. Chung, Byoungho Lee, and coauthors reported that the three primary colors (red, green and blue) were prepared by adjusting the thickness of a WO_3_ thin film.^372^ In the relevant electrochemical process, the color of corresponding materials was reversibly changed by regulating the redox state of WO_3_ and the intercalation/deintercalation of Li^+^. By using photolithography technology, they successfully produced a colorful pattern of 789 × 463 pixels (where each colorful pixel was 75 μm × 75 μm, including three RGB stripes with the width of 20 μm). The fabricated EC pixel display could be turned “on” and “off” under electrical stimulation, as shown in Figure 22d.

There are some other novel device construction strategies that can be used for multicolor EC pixels. For example, it is well-known that LCD monitors use color filters to achieve colorful pixels, and this strategy has been fully commercialized. Can colorful EC pixel displays be realized with color filters? Based on this question and innovative concept, Jang H. Kwon et al. reported full-color reflective displays based on the RGB color mode, which included both a color filter (CF) and a transmittance controllable EC color filter (TCECF).^373^ The utilized CF and TCECF could selectively transmit visible light, and a black ECD was used to turn the pixels on/off to show the corresponding colors. In addition, EC leuco dye derivatives with multiple colors (red, green, and blue) were induced in the display to replace the CFs for a higher diffuse reflectance.

Although these demonstrated results and application prospects are attractive, there are still urgent problems that need to be solved. For example, in the lateral/horizontal arrangement of EC subpixels, because all subpixels are distributed on the same layer, not all subpixels are in the “on” state at any time when displaying a colorful image. These subpixels in the “off” state will produce a sense of blankness on the display. Thus, the fill factor of each color, which is defined as the ratio of effective displayed pixel area and the total pixel area, is relatively low. Determining how to solve this problem economically and conveniently is a major challenge in this field.

#### 4.1.2 Vertical Stacking Arrangement

**Figure 23:**
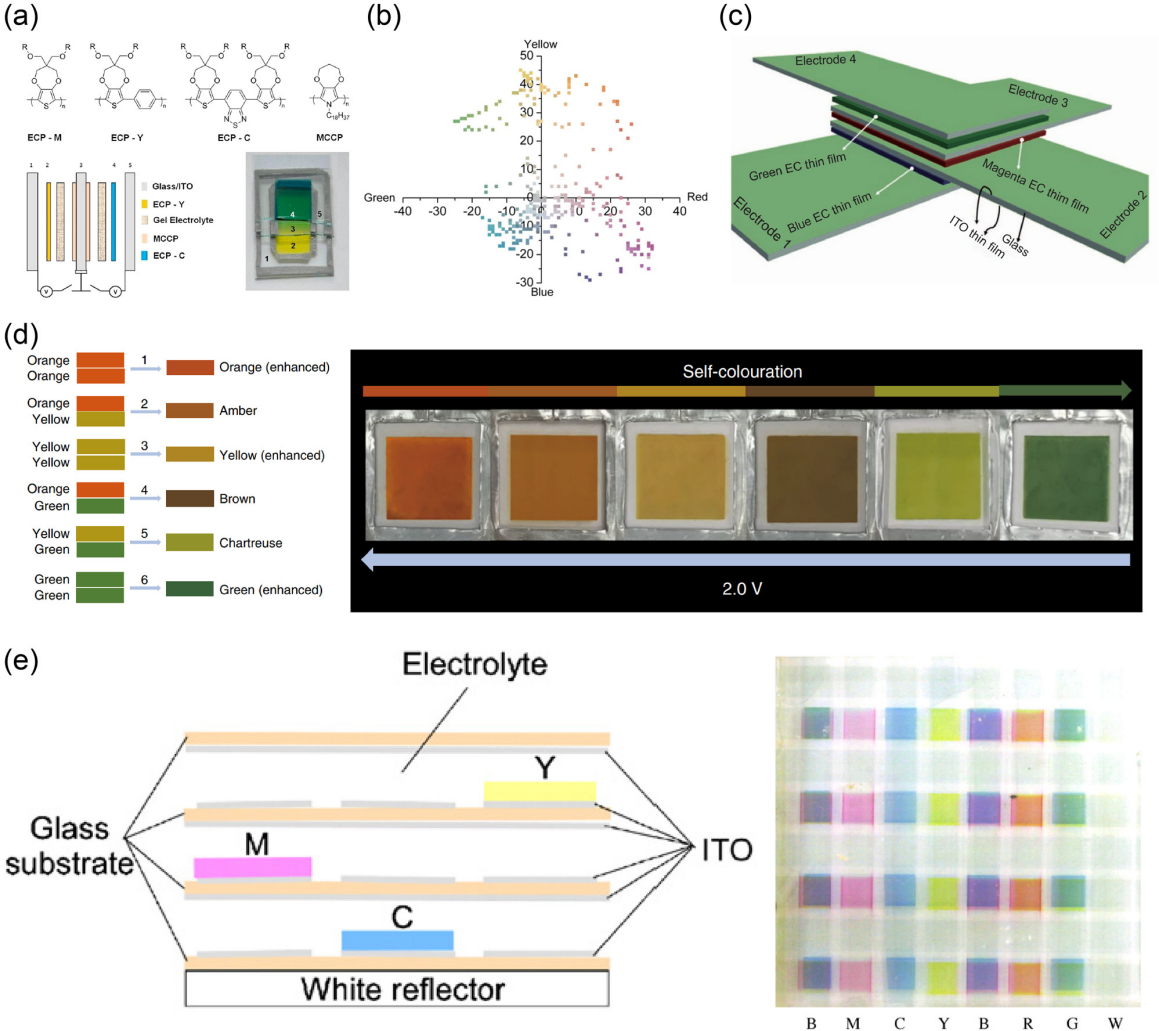
(a) The structure of EC CPs and dual-active devices based on a vertical arrangement. (b) The a* b* values of the colors generated by dual-active ECDs. Reproduced from ref (376). Copyright 2014 American Chemical Society. (c) The structure of an ECD with three EC layers and four orthogonal thin-film electrodes. Reproduced from ref (377). Copyright 2015 Springer Nature. (d) Schematic and photos of vertical arrangement for combining orange, yellow, and green. Reproduced from ref (378) (CC BY 4.0). Copyright 2020 Springer Nature. (e) Schematic and image of a three-layered ECD with 8 × 8 pixels and passive matrix driving mode. Reproduced from ref (379). Copyright 2008 Elsevier B.V.

The vertical stacking arrangement of EC subpixels can largely solve the fill factor deficiency of the aforementioned horizontal/lateral arrangement.^374,375^ In 2014, John R. Reynolds’s group reported an impressive multicolor tunable EC display based on this vertical arrangement and dual-active ITO,^376^ as shown in Figure 23a. In their work, three PProDOT-derived materials with cyan, magenta and yellow primary colors were used as EC materials. A wide color range was achieved via the secondary mixing of primary colors (Figure 23b). Another type of interesting vertically arranged durable multicolor prototype ECD with three EC layers and four orthogonal thin-film electrodes was also reported by Sean X.-A. Zhang and Yu-Mo Zhang et al.^377^ In this work, the “on/off” states of multiple colors (green: 617 nm, blue: 583 nm, and magenta: 551 nm) were controlled individually or simultaneously (Figure 23c). In the study of transparent inorganic multicolor EC displays with Zn-sodium vanadium oxide (Zn-SVO), Abdulhakem Y. Elezzabi, Haizeng Li, and coauthors reported a similar vertical arrangement for secondary colors.^378^ The as-prepared Zn-SVO film exhibited reversible switching between three colors (orange, yellow, and green) originating from the electrochemically driven redox process of vanadium oxide and the insertion/extraction of Zn^2+^ under different voltages (0.2 to 2.0 V). Interestingly, by designing the color overlay based on vertical multilayer construction, the color palettes were efficiently broadened into six colors (orange, amber, yellow, brown, chartreuse, and green) (Figure 23d).

The above-mentioned studies proved the feasibility of using EC materials/devices with different colors in the vertical stacking arrangement for secondary colors. Norihisa Kobayashi and coauthors further extended this concept to the field of pixel displays.^379^ In this report, terephthalate derivatives and hexylviologen were used as EC materials for three primary colors (magenta, cyan, and yellow). The authors demonstrated a color electronic paper based on a three-layered ECD with 8 × 8 pixels and passive matrix driving mode (Figure 23e).

Although the vertical arrangement mode is expected to solve the poor fill factor deficiency of the lateral/horizontal arrangement, some other problems need to be considered and solved urgently. First, the complex device structure significantly increases the manufacturing cost. Second, due to the limited transmittance of each utilized transparent electrode, the vertical superposition of multilayer electrodes clearly reduces the overall transmittance and optical modulation (and CR) of the device. Third, EC materials in different layers require different visual focusing distances during observation. Therefore, if the device is too thick and/or the pixel is too small, the phenomenon of pixel drift will appear, which affects the resolution.

#### 4.1.3 Single-Pixel Device with Adjustable Colors

As a widespread phenomenon, many EC devices/materials can achieve multiple color changes. Therefore, in many cases, it is not necessary to contain all types of subpixels within one EC pixel. The color-tunable characteristics of a single-pixel device can be used to achieve the desired multicolor, which further reduces the number of subpixels and significantly improves the fill factor. Due to the excellent modifiability and multivalence of many EC materials, many studies have proven that the strategy of achieving multicolor or different visible spectra through multistep redox reactions is feasible.^380−383^ In addition, modulating the parameters of metal deposition is also an efficient way to broaden color palette in the study of EC plasmon materials.^384−386^ For example, Sheng Chu, Guoping Wang, and co-workers achieved the dynamic adjustment of colors in real time with this interesting plasmonic tuning strategy.^387^ In this work, through actively controlling the deposition time of Ag^+^ on the Au core, remarkable plasmonic structural colors (e.g., red, green, and blue) were produced. And then, a multicolor EC pattern was demonstrated successfully.

**Figure 24:**
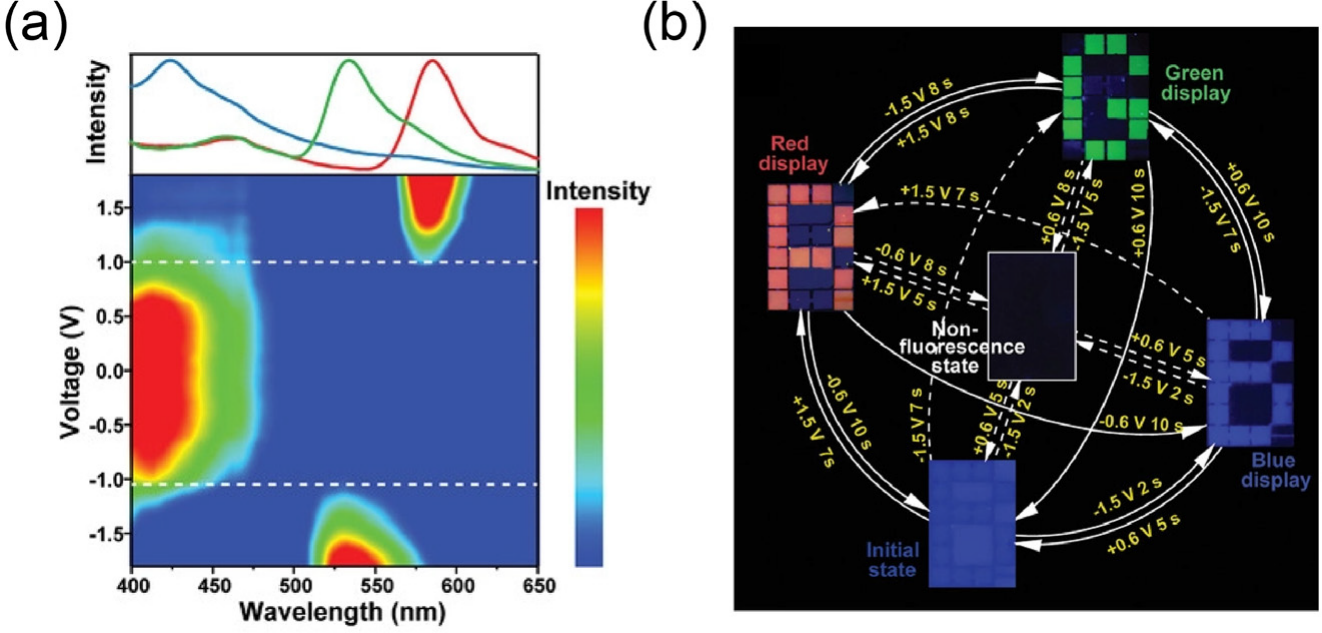
(a) Fluorescence spectra and (b) photos of the RGB-based EFC alphanumeric device under different voltages. Reproduced from ref (388). Copyright 2020 Wiley-VCH GmbH.

Another potential solution for achieving tunable multicolors is to reasonably integrate multimaterials within a single-pixel device. For example, Sean X.-A. Zhang, Yu-Mo Zhang, and co-workers reported a single-pixel EFC device with tunable RGB primary colors.^388^ In this work, a urea derivative and a rhodamine derivative were chosen for the red fluorescence under a positive voltage stimulus. p-Benzoquinone and fluorescein were chosen for the green fluorescence under a negative voltage stimulus. According to the classic work reported by Valérie Alain-Rizzo, Pierre Audebert, and coauthors,^389^ the fluorescence intensity and color of triphenylamine derivatives were closely related to the molecular redox states. Based on this, polytriphenylamine was chosen as the EFC material with blue fluorescence. Then, three kinds of EFC materials were rationally arranged in a single-pixel device. The as-prepared device can successfully achieve interference-free red, green, and blue fluorescence under the stimulation of positive voltage, negative voltage, and no voltage, respectively (Figure 24). Based on a similar idea, a multicolor EC display was demonstrated also in 2019.^390^

### 4.2 The Driving Mode

To realize the arbitrary switching and dynamic adjustment of displayed information, patterned electrodes with a matching driving mode are required. The preparation approaches for common patterned electrodes have already been briefly introduced above, so we will not repeat them here.

**Figure 25:**
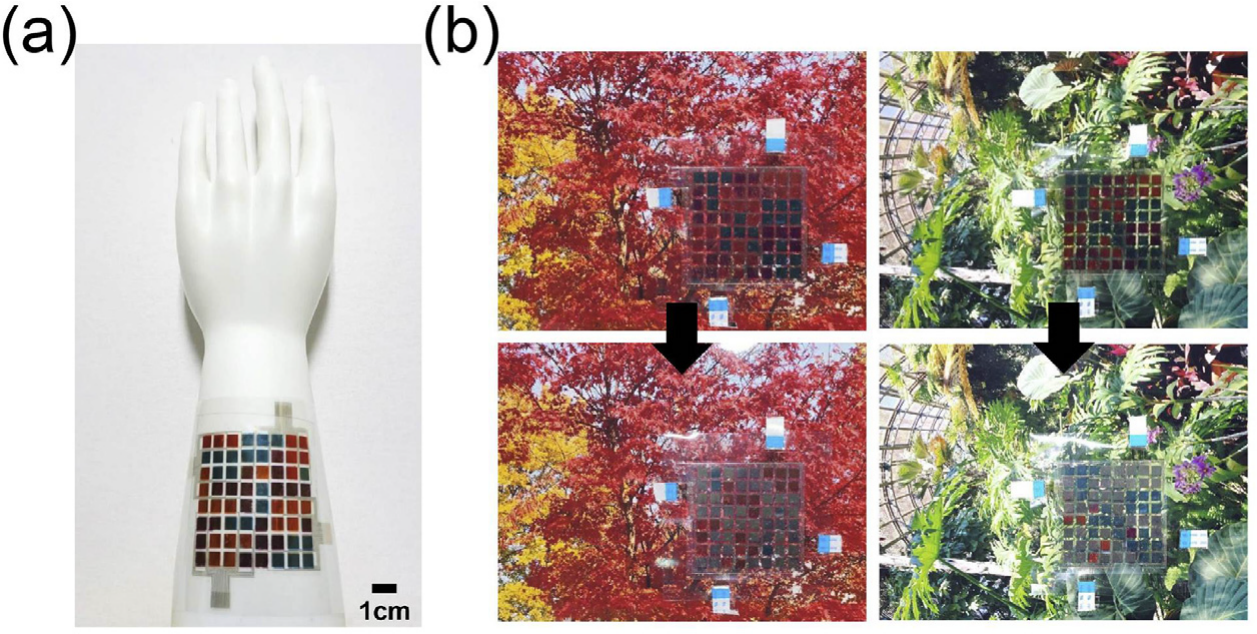
(a) Photo of a colorful EC pixel display with the point-to-point driving mode on a model hand. (b) Photos of the pixel display over various backgrounds for adaptive camouflage applications. Reproduced from ref (392). Copyright 2020 Elsevier Ltd.

When the number of pixels in an EC display is small, the point-to-point driving mode can be an excellent choice.^391^ With this driving mode, each EC pixel has its own electrode lead controlled by an external circuit. For example, a flexible EC multicolor pixel display (containing 8 × 8 pixels) was reported by Do H. Kim et al., as shown in Figure 25a,b.^392^ In alphanumeric pixel displays (a kind of EC segmented display), this kind of point-to-point driving mode is also very popular. Moreover, the crosstalk of electrical signals is very weak, so this driving mode is suitable for controlling microscale pixels, for example, micro displays on a chip.^393^ However, when there are a large number of display units (pixels), the number of electrode leads and contacting pads increases dramatically, and related chip area and manufacturing costs also significantly increase.^394^ Obviously, the point-to-point driving mode is not appropriate in this scenario.

**Figure 26:**
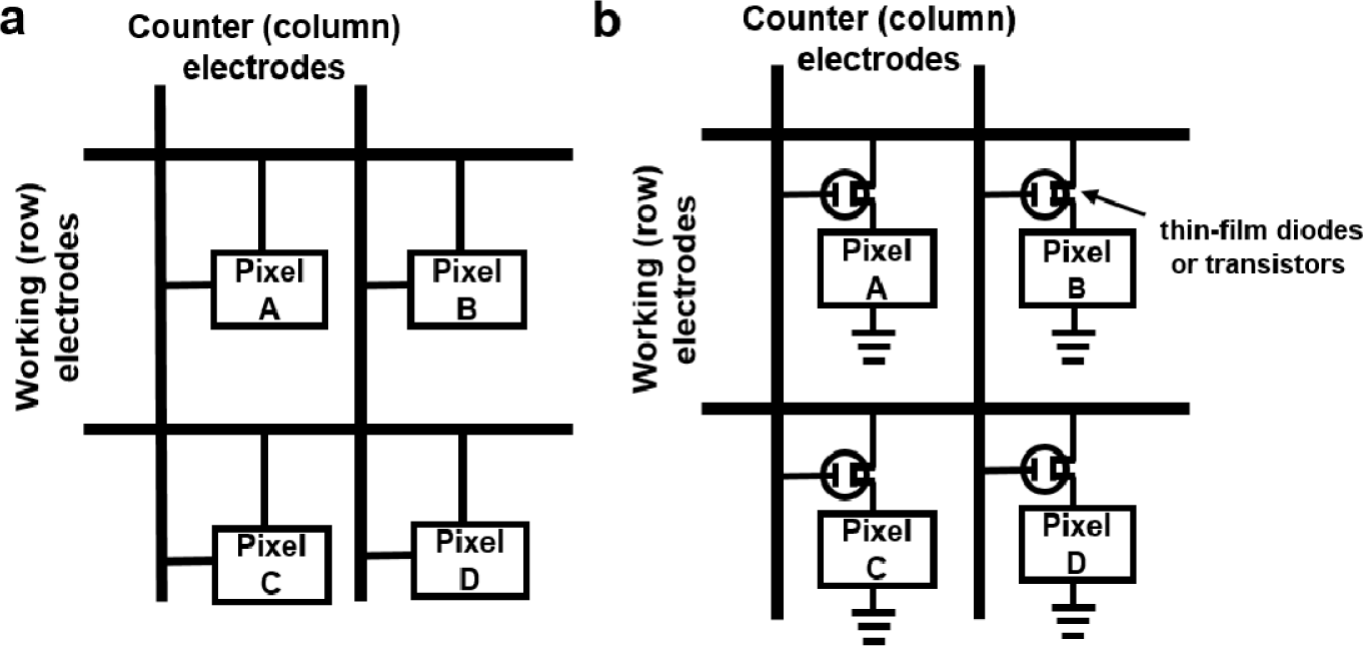
Two typical matrix driving modes: (a) passive matrix and (b) active matrix.

In addition to the above-mentioned point-to-point driving mode, matrix driving modes, including the passive matrix (PM) and the active matrix (AM), are widely used in modern electronic displays. And such concepts are often imitated and adopted in emerging EC displays (Figure 26). In these modes, interdigitated (cross-finger) electrodes are introduced, and EC pixels are driven by adjacent electrode wires. Such matrix driving modes are more suitable for EC displays with a large number of pixels.

#### 4.2.1 Passive Matrix and Signal Crosstalk

In PM-based ECDs, the pixelated EC materials are located at the intersection of the working (row) electrode and the counter (column) electrode, as shown in Figure 26a. When the row electrode and the column electrode have electrical signal input, the EC pixel at the intersection area generates the optical signal (color) response. PM-based EC displays have a high pixel fill factor due to their simple device structures. Moreover, thanks to the optical memory effects (even bistability) of many EC materials/devices, the as-prepared displays can achieve a remarkably stable static display. However, a serious signal crosstalk problem often occurs in PM-driven EC displays originating from unwanted charge transfer. Determining how to effectively avoid (or reduce) the signal crosstalk is a key issue.

**Figure 27:**
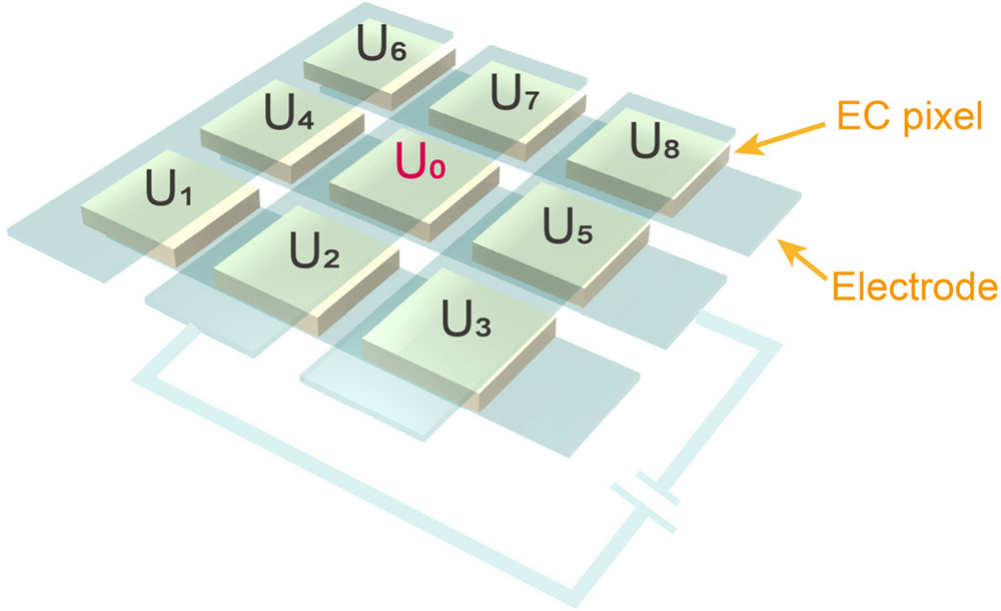
Schematic of the crosstalk voltage in a PM-based EC pixel display.

According to the research of Hiroyuki Nishide, Kenichi Oyaizu, and coauthors,^395^ charge transfer through the electrode substrate was the predominant route of electrical signal diffusion. Because each electrode was in direct contact with multiple EC pixels, the weaker surrounding charge transfer may also lead to the response (color change) of EC materials, although the charge transfer was fastest in the two facing electrodes due to the reduced electrical resistance (ohmic drop) and/or faster ion transport originating from the nearest distance. To solve this problem, related electrolytes should be patterned to limit the lateral ion transport of molecules and ions between pixels. In addition, the crosstalk voltage is another cause of severe picture distortion.^400^ That is, the applied voltage not only forms a potential at the target pixel (for example, U_0_, as shown in Figure 27), but can form additional potentials at surrounding pixels (U_1_ to U_8_). Even though this kind of additional potential is relatively weak, it is still possible to produce color changes and optical signal crosstalk. To solve this problem, it is desirable to optimize the response threshold voltage of EC materials/devices.

In 2002, H. W. Shin et al. proposed a typical PM-based EC pixel display after solving the related signal crosstalk problem.^400^ In this interesting report, TiO_2_–WO_3_ and TiO_2_–CeO_2_ were used as EC materials on the working electrode and active materials on the counter electrode, respectively. An insulating partition was manufactured with negative photoresist (SU-8) to avoid lateral ionic conduction between EC pixels. Besides, an additional protected layer (0.2Li_2_O–0.2CeO_2_–0.6SiO_2_) improved the threshold effect. Finally, the authors fabricated an addressable EC pixel display (50 × 50 pixels, pixel size: 4 × 4 mm^2^).

**Figure 28:**
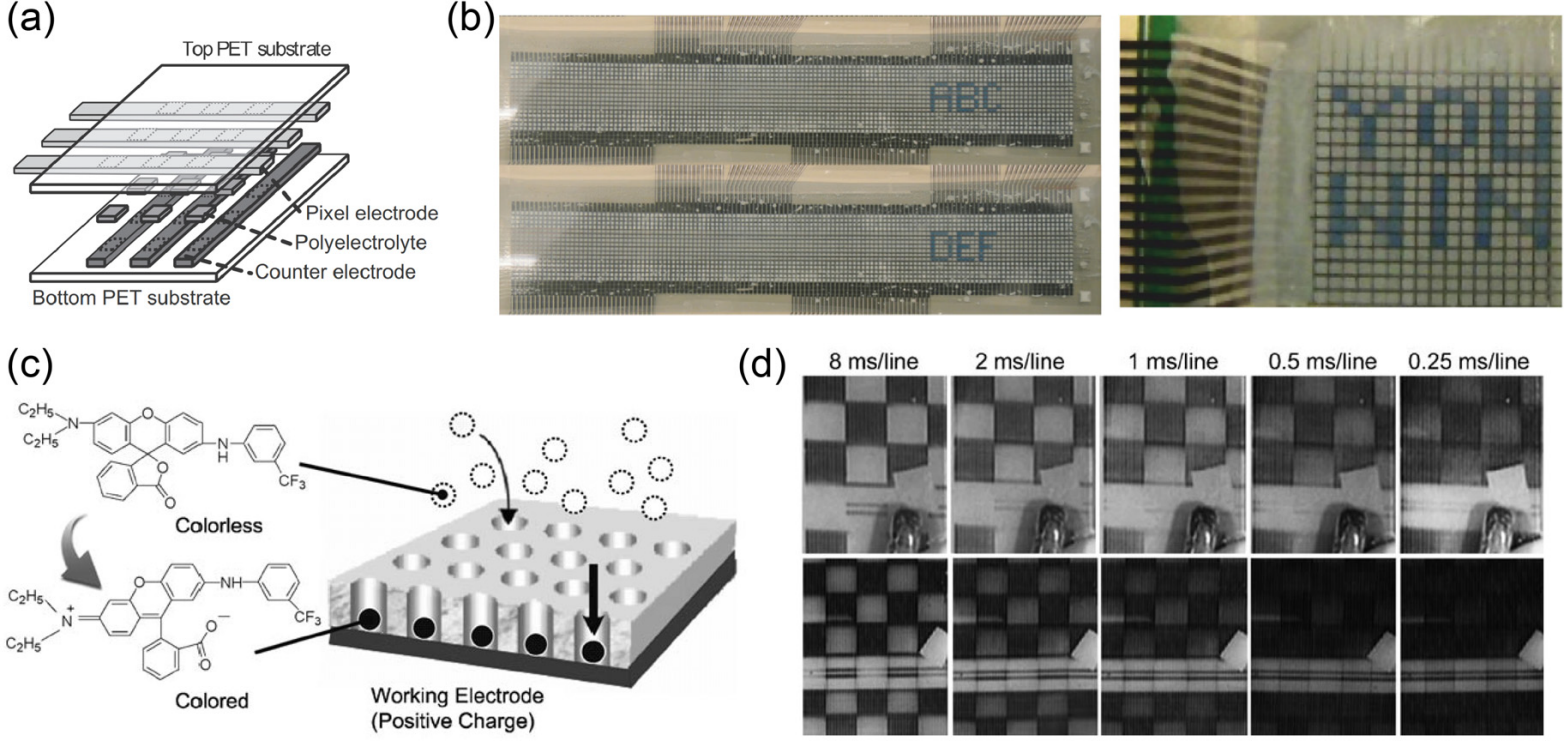
(a) Schematic of an all-printed PM-based EC pixel display with spontaneously separated electrolytes and (b) corresponding images. Reproduced from ref (401). Copyright 2013 Elsevier B.V. (c) An EC display constructed with an organic dye and a mesoporous TiO_2_ electrode. (d) Comparison of display images containing TiO_2_ electrodes with (top) and without (bottom) mesoporous structures. Reproduced from ref (402). Copyright 2010 Wiley-VCH GmbH.

In addition to the above-mentioned photoresist partition for limiting the ionic conduction between pixels, Peter A. Ersman et al. designed and reported the use of a novel spontaneously separated electrolyte to achieve this goal (Figure 28a).^401^ In this work, PEDOT:PSS was used as the EC pixel electrode, and carbon paste was used as the counter electrode. Polystyrene (PS) was used to fabricate the separation gaps between pixels. The water-based polyelectrolyte used could be spontaneously separated and confined to the desired pixel area due to the difference in surface tension between the electrode material and PS. On the other hand, the device had an obvious threshold voltage due to the required turn-on voltage of the electrochemical half-reaction on both electrodes. Finally, an impressive EC pixel display with the individually addressed pixels was developed successfully (Figure 28b).

Porous nanostructures can also be used to reduce the dimensions of ionic conduction. Based on this principle, Yusuke Yamauchi, Wu Weng, and coauthors designed and fabricated a high-speed PM-based EC display with a mesoporous TiO_2_ electrode and organic dyes (EC molecules) (Figure 28c).^402^ They prepared a TiO_2_ thin-film on the working electrode with a highly ordered vertical mesoporous structure. This EC system had two major advantages. One was that the mesoporous film with a large surface area could be used to store the required EC organic dyes. The other was that their vertical mesopores effectively hindered the lateral diffusion of embedded organic dyes and electrolytes and provided clear EC images at very high drive speeds (less than 1 ms per line) (Figure 28d). In 2013, Yusuke Yamauchi et al. designed and fabricated an Al-doped TiO_2_ film with ordered mesoporous structures.^403^ This approach prevented the rapid crystallization of TiO_2_, and drastically improved the thermal stability. As stated by the authors, this type of Al-doped TiO_2_-based EC display with a PM driving mode might has important applications in building future reflective display devices.

The above-mentioned classic reports undoubtedly have stronger reference value for the whole field. However, to further enhance the display performance for commercial applications, the following issues need to be considered. First, the resolution and aspect ratio (ratio of thickness to width) of the patterned electrolyte should be improved. The thickness of the electrolyte layer for common ECDs is usually tens of micrometers, or even hundreds of micrometers. Therefore, when the size of a pixel is at the micron level (for example, tens of microns) for high-resolution displays, the patterned electrolyte needs a very-large aspect ratio to achieve a high pixel fill factor, which is a major challenge for the micro/nanofabrication technologies of related materials. Second, relevant materials should maintain good compatibility and stability. For example, if the patterned electrolyte is prepared through introducing insulating materials (e.g., commercial photoresist), the insulating materials must have adequate photostability and thermal-stability for practical applications. Meanwhile, to avoid interfering with EC performance, the insulating materials should also have acceptable optical states (e.g., colorless), resistance to solvents and plasticizers, and good electrochemical stability.

In the future, we predict that the above issues are expected to be solved by the following potential approaches. (i) Optimize the materials and patterned preparation processes, for example, developing high-performance materials that can also achieve high-resolution pixelation. (ii) Reduce the thickness of the electrolyte layer. This is helpful to increase (decrease) the transfer speed of ions in the vertical direction (lateral direction), and avoid the signal crosstalk problem, even when electrolytes are not patterned. (iii) Introduce low-dimensional (1D or 2D) ionic conductors (such as mesoporous membranes and low-dimensional ionic liquid crystals^396−399^) to construct the ionic/molecular moving channel and limit the lateral ion transport between pixels.

#### 4.2.2 Active Matrix

**Figure 29:**
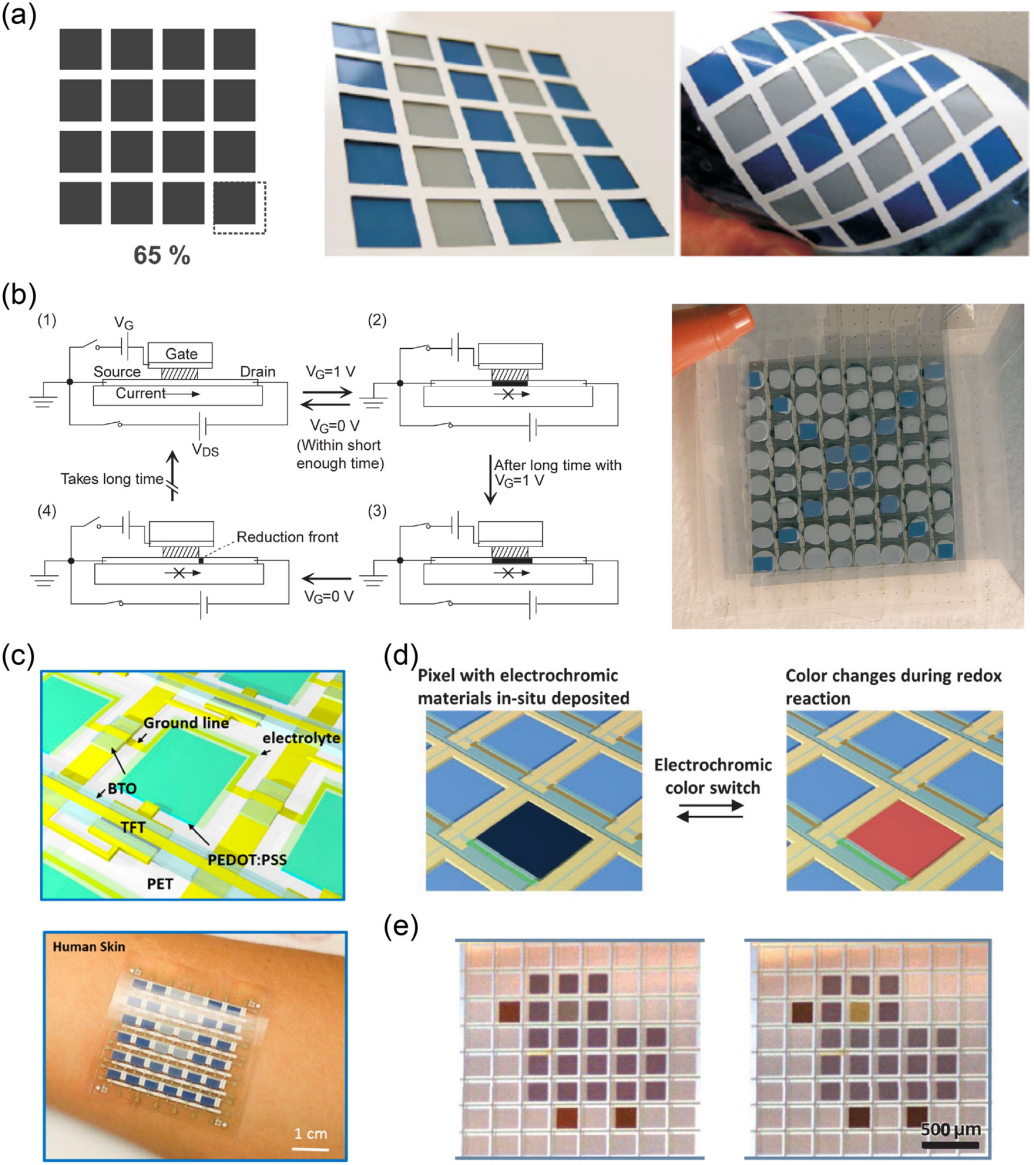
(a) A printable all-organic flexible AM-based EC pixel display based on PEDOT:PSS. Reproduced from ref (409). Copyright 2007 Wiley-VCH GmbH. (b) A flexible AM addressed EC pixel display with OECT and EC pixels on different sides of the PET substrate. Reproduced from ref (405). Copyright 2012 Wiley Periodicals, Inc. (c) A flexible AM-based EC pixel display with fully screen-printed techniques. Reproduced from ref (406). Copyright 2016 American Chemical Society. (d) EC pixels driven by an a-IGZO TFT and (**e**) its displayed image of a bird. Reproduced from ref (411). Copyright 2021 Wiley-VCH GmbH.

In mainstream light-emitting displays such as LEDs and OLEDs, the AM driving mode is widely used; it mainly uses a thin-film transistor (TFT) to drive/control active pixels. EC pixel displays can also rely on thin-film diodes or transistors to achieve the address of EC pixels (Figure 26b). The introduction of thin-film diodes or transistors sacrifices the pixel fill factor to some degree and leads to higher costs and more complex production processes. However, compared to PM-based EC pixel displays, a higher refresh rate and lower energy consumption can be achieved.^404^ Moreover, since the applied voltage is limited to the targeted pixels, the signal crosstalk problem is largely avoided. Therefore, this driving mode has received remarkable attention.^405−408^ For instance, Magnus Berggren et al. reported a printable all-organic AM-based flexible EC display.^409^ In this work, they used PEDOT:PSS as EC materials and conducting lines. By combining a three-terminal electrochemical transistor with the EC display cell architecture, the authors successfully obtained an ideal ECD with a fill factor of up to 65% (Figure 29a).

As one of the key factors of AM-based pixel displays, the structure and function of thin-film diodes or transistors have a significant impact.^410^ That is to say, better display performance can be achieved through an elaborate structural design (for instance, the layout of thin-film diodes/transistors and the optimization of circuits). Peter A. Ersman and Magnus Berggren et al. constructed a novel device structure by placing the organic EC pixel and its corresponding organic electrochemical transistor (OECT) on different sides of a flexible PET substrate (Figure 29b).^405^ Compared with traditional devices with all electronic components on one side, this approach decreased the area proportion of the nondisplay components. Therefore, the device clearly had a higher resolution and pixel fill factor. Based on this, the authors successfully demonstrated an AM-based EC display with 8 × 8 pixels.

The preparation process of AM-based EC pixel displays usually requires many steps, for example, step-by-step preparation of patterned electrodes, patterned EC materials, transistors, and suitable electrolytes. Therefore, designing a simpler process flow is undoubtedly an urgent and important topic for reducing the difficulty and cost of future industrialization. To overcome this technical challenge, Chongwu Zhou et al. designed and fabricated a flexible AM-based pixel display with fully screen-printed techniques (Figure 29c).^406^ They cleverly used silver nanoparticles, semiconductor single-walled carbon nanotubes, and barium titanate as conductors, conductive channels, and insulators, respectively. These materials were coated onto the PET substrate by step-by-step printing to prepare the desired TFTs. The authors combined the PEDOT:PSS-based ECD prepared by digital printing with the printed TFTs, and finally realized the desired full-screen-printed AM-based EC pixel display. The successful demonstration of this easy-to-replicate simplified preparation method was expected to show good application prospects for large-area, flexible, low-cost EC displays.

In recent years, amorphous indium-gallium-zinc oxide (a-IGZO) TFTs have been widely used in multiple microelectronic devices, for example, displays (especially existing AM-driven LCDs or OLEDs) and sensors. In EC pixel displays, Bin Bao, Daniil Karnaushenko, Oliver G. Schmidt, and coauthors reported using a-IGZO TFT as an AM driving module to address EC pixels (Figure 29d).^411^ The as-prepared prototype device was composed of multiple EC pixels, each of which was only 200 × 200 μm^2^ in size, as shown in Figure 29e. Upon adjusting the color changes in different pixels, the displays successfully achieved pixelated patterns (such as “birds”).

#### 4.2.3 Summary of the Driving Mode and Its Development Assessment

In existing studies, many scientists proposed/explored promising prototypes of EC pixel displays and demonstrated the expected feasibility in principle. To further meet the needs of the market, fabricating a more ideal driving mode is an important goal in future research.

**Figure 30:**
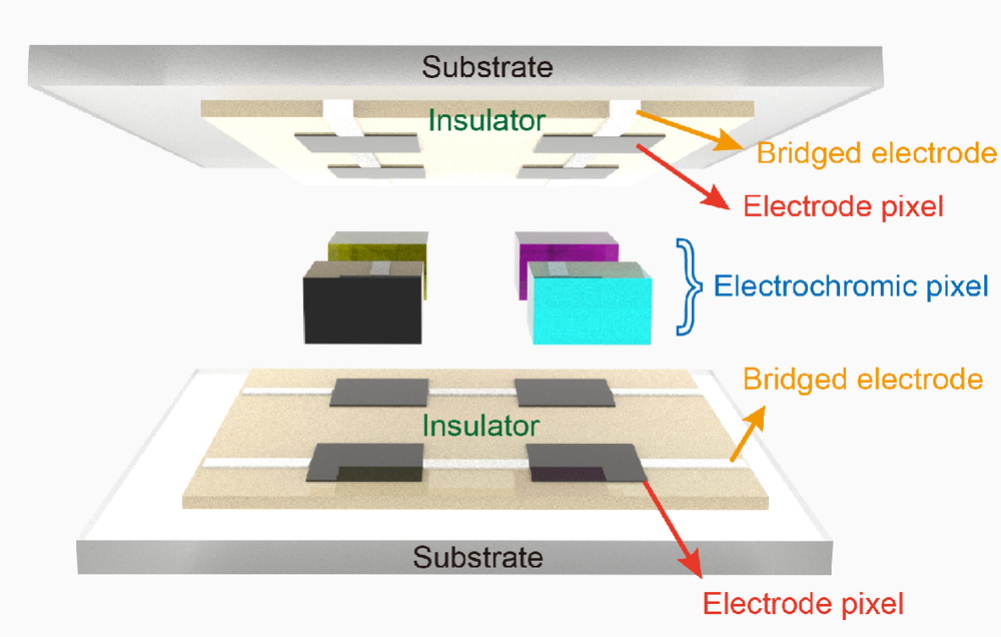
Schematic of EC pixel displays with electrode pixels and bridged electrodes as the driving electrodes.

The so-called driving mode here is essentially the control mode of EC pixels; corresponding research mainly focuses on the design and construction of driving electrodes. The ideal driving electrode needs to be able to achieve excellent performance including high resolution and pixel fill factor, and inexpensive electrode machining process. To achieve this goal, we believe that the following factors can be taken into consideration. The schematic in Figure 30 is used as an example.

1. The thickness of EC pixels. Reducing the thickness of EC pixels to decrease the resistance in the vertical direction is expected to alleviate the above-mentioned signal crosstalk problem. In addition, the thinner the EC layer, the faster the ion transport in the relevant EC process and the lower the required driving voltage. On the other hand, EC molecules with higher molar absorption coefficients are more popular, because gentler stimulation can be used to achieve the desired optical modulation.
2. The matching of electrodes. As a typical transmissive or reflective display, the electrodes should be transparent on at least one side. However, the conductivity of electrode materials with good transparency (i.e., ITO, FTO, etc.) is generally not ideal, and the transparency of highly conductive materials (such as Au, conductive carbon black, etc.) is also usually not satisfactory. To meet the requirements of high conductivity and high transparency, in addition to developing more ideal electrode materials, we can also integrate two (or more) electrode materials. Here, determining how to make these electrode materials with different properties fully compatible with each other during the integration process and how to minimize (or completely eliminate) interface resistance are urgent problems that need to be solved. Some potential methods for reducing interface resistance reported by Tao Deng’s group might provide important reference value. For example, the authors prepared Ag NW thin films that self-assembled at the air/water interface under solar illumination.^412^ This kind of welding method does not depend on the substrate. Therefore, it is expected to be used to fabricate flexible electronic devices when combined with flexible substrates (e.g., PDMS). Other welding methods (such as thermal annealing and pressure welding) have been widely studied.^413−415^ In addition, it is worth emphasizing that the introduction of liquid/low-melting metal caulking may also be a feasible solution.^416^
3. Selection of the bridged electrode between EC pixels. Although there are many restrictions on the selection of electrodes that are in direct contact with EC pixels (electrode pixels), there may be more options for electrodes in remaining areas (bridged electrodes). Because such bridged electrodes are not in the main observed region, their transparency can be sacrificed for better electrical conductivity. In other words, the electrode pixel material and bridging electrode material in Figure 30 may be the same or different. If the width of the bridged electrodes decreases dramatically, then the ratio of the pixel area to the bridged area becomes very large. Under such favorable conditions, the aforementioned crosstalk may also be relatively unobservable. Meanwhile, the thickness and length of the bridged electrodes should be considered. For example, if the electrode is too thin or too long, then its conductivity is also seriously affected.

### 4.3 Summary of Electrochromic Pixel Displays

Compared to EC segmented displays, the EC pixel display is more complex. For an ideal full-color pixel display, many factors and details must be carefully considered and optimized, including the required materials (e.g., colorful EC materials and electrode materials), device structures (e.g., the arrangement of EC pixels/subpixels), and driving modes. However, complex and ingenious manufacturing processing enables better application value and usage experiences. EC pixel display is one of the most promising next-generation nonemissive displays that can realize tunable and colorful images and/or information, and is expected to become an indispensable part of our daily life.

However, compared with commercialized displays, EC display prototypes have not yet achieved mature micrometer-level pixels that can truly meet market demand, whether in the PM or AM driving mode. Moreover, due to the performance limitations of commonly used EC materials, the colors of demonstrated EC pixel displays are relatively monotonous. The long-awaited high-performance full-color EC pixel displays based on CMYK (or RGB) color mode have not yet been realized.

## 5 Progress and Challenges of Electrochromic Displays in Future Commercial Development

### 5.1 Commercial Progress in Electrochromic Displays

**Figure 31:**
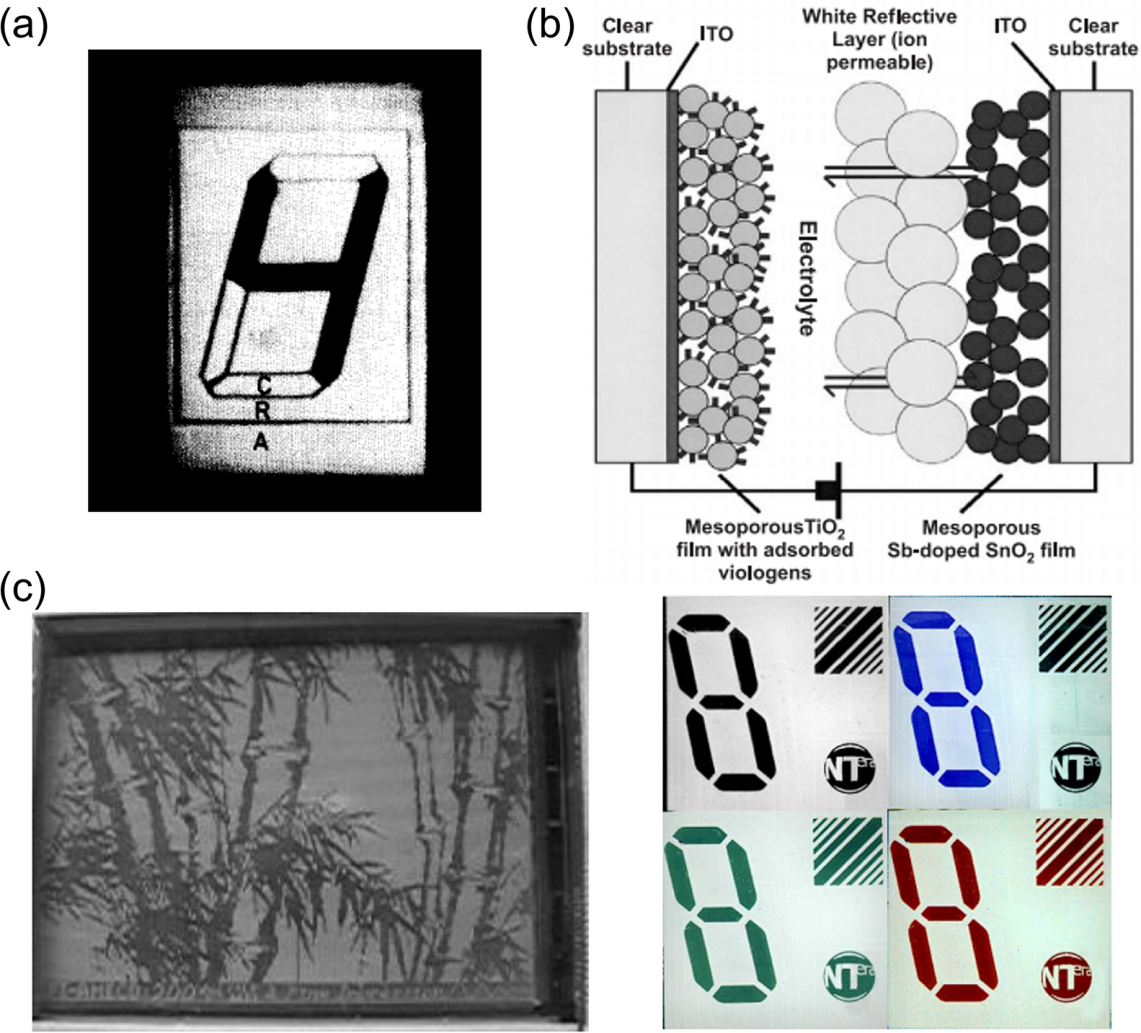
(a) The EC display reported by Philips Research Laboratories. Reproduced from ref (417). Copyright 1973 AIP Publishing. (b) The device structure of NanoChromics and its corresponding multicolor images. Reproduced from ref (418). Copyright 2003 Elsevier B.V. (c) The QVGA AM-based EC display with a high resolution of 200 dpi. Reproduced from ref (419). Copyright 2012 John Wiley and Sons.

As is well-known, the commercialization of EC displays has been explored for a long time. In the 1970s, Philips Research Laboratories reported the first simple monochrome protype EC digital display based on heptyl viologen (Figure 31a).^417^ The display had a respectable high CR (20:1), short erasure times (ranging from 10 to 50 ms), and good reversibility (more than 10^5^ cycles). The successful demonstration of this display product has greatly stimulated global interest in EC materials and boosted related R&D and commercialization. However, there are still problems that need to be solved. The first problem is that the self-aggregate and crystallize of viologen derivatives during their EC process, which seriously affects the cyclic life. The second problem is that related EC reaction is usually carried out in a solution or gel. Therefore, the active molecules easily diffuse away from the electrode. This blurs the displayed image and hinders it from being quickly erased by the current. At the same time, this problem also induces significant challenges and difficulties in manufacturing processes such as device sealing. The third problem is how to maintain the long term memory effect (bistability) of the displayed information.

In response to the above technical problems, in 2003, NTera Ltd. developed a representative viologen-based EC display called NanoChromics.^418^ They cleverly used mesoporous TiO_2_-anchored viologen as the EC material and Sb-doped SnO_2_ as the counter electrode material. Then, several viologen derivatives with different substituents were used for multicolors (Figure 31b). The displays demonstrated good cyclic life and durability. For example, the blue one could undergo 50,000 cycles at both room temperature and 50 °C; its contrast was maintained for more than 888 h under constant voltage, which indicated a good service life.

In 2006, Cambridge Research Laboratory of Epson, Seiko-Epson Corporation, and NTera Ltd. coreported a QVGA AM-based EC display with a high resolution of 200 dpi.^419^ This demonstration was also based on TiO_2_-anchored viologens as EC materials (Figure 31c). The authors found that the uniformity of TiO_2_ formed on the cathode was very important for achieving the desired high-quality EC performance. The display was successfully driven with integrated driver electronics fabricated by low-temperature polysilicon thin-film transistor (LTPS-TFT) technology. The display also showed high contrast, high image quality, wide viewing angle, etc. In addition, a remarkable image retention time (bistability) was obtained: the displayed image could be partially read after 3 days without voltage treatment.

In 2008, Samsung Advanced Institute of Technology produced a 4.5-in. EC display driven by PM.^420^ The signal crosstalk problem was impressively solved by using the isolated gel electrolytes. Next, RICOH Company developed a multilayer EC display (mECD) consisting of display units based on subtractive color mixing theory.^421^ In this work, researchers demonstrated the use of a 3.5-in. QVGA LTPS-TFT AM panel as the driving module. Each TFT-driven mECD unit was cleverly constructed from a shared counter electrode unit and three vertically stacked (cyan, magenta, and yellow) EC units (where each display unit is composed of a pair of EC substrates and a corresponding working electrode). The EC compounds used were adsorbed by the nano-TiO_2_ porous electrode. The monochrome EC display achieved an ideal resolution of 113 ppi and could display grayscale images. By using three specially prepared organic EC dyes with different colors, a multicolor display was successfully developed.

To date, the most widely known commercial ECDs are the antiglare rearview mirror and EC porthole on the Boeing 787 Dreamliner. These ECDs can automatically adjust the brightness, thereby eliminating dangerous glare from the rear vehicle headlights or improving the comfort level within the aircraft. Gentex has been extensively involved in the above two fields for many years and has been widely accepted in the marketplace.^111^ In addition, the rise of emerging companies (Heliotrope Technologies, Tynt Technologies, MiRuo China, ChromoGenics, SageGlass, etc.) has also continuously added vitality into this field. In recent years, EC smart windows have been applied to the windows of cars and buildings. The grayscale ability of EC materials/devices makes it possible to control the transmittance of these “glasses” as needed. For example, Ambilight Inc. offers large-area (1.9 m^2^) EC sunroofs for electrical vehicles created through roll-to-roll manufacturing processes. This successful product has laid an important foundation for further development of EC displays.

EC technologies have also begun to play an important role in portable consumer electronics such as mobile phones. In January 2020, the first smart concept phone equipped with EC technology was launched by OnePlus (as OnePlus Concept One). In this phone, the back camera module is hidden under an EC module and only appears when in use. This improves the overall look/feel of the device. Additionally, the color-switching speed is only 0.7 s, which does not affect the shooting process. At present, several mobile phone manufacturers are attempting to integrate EC technologies. For example, Ambilight Inc. teamed up with OPPO to successfully achieve mass production of an EC phone back cover with color-variable pictures (between silver and magenta).

In addition, existing commercialized EC display devices are mainly concentrated in electronic price tags, flexible electronic paper, etc. Ynvisible, Acero, and NTera Ltd., have made considerable effort and achieved great progress in developing these printing EC products. In summary, with the efforts of many related R&D teams, rapid progress has been made in the industrialization process of EC materials/devices. Although these products may not be displays or just low-end display components, they all represent the commercial development of electrochromics. This has paved the way for the realization of full-color EC displays in the future.

### 5.2 Existing Challenges and Possible Solutions

To date, EC displays have undoubtedly achieved unique commercial value with respect to future display fields due to their attractive advantages. Regrettably, although many well-known R&D institutions and companies have proposed various prototypes, there are still no mature EC displays that can truly be used in daily life. This means that related EC display prototypes that have been demonstrated thus far still cannot meet the needs of consumers and the market. The deficiencies mainly exist in the following four aspects.

#### 5.2.1 Materials

We discussed the material aspect in detail in Section 2. Rich material selectivity has been achieved now in the related field. At present, such research on materials mainly focuses on two approaches: (i) optimizing the performance of existing materials and (ii) developing new materials, which require more fundamental studies of the underlying mechanism and exploration of composite materials. In addition to what we have discussed above, two additional issues need to be noted, as presented below.

(i) The matching and compatibility of multiple materials should be considered. For a classic ECD (containing electrodes, ECL, ITL, and ISL), multiple chemical and physical reactions are involved in the EC process including electron transfer (on the electrode and at the interface between the electrode and redox-active materials), electrochemically driven redox processes, ion transfer inside the device, etc. Therefore, excellent overall performance and functionalization requires all components to maintain good compatibility and matching. Taking ISL as an example, its critical effect on device performance has been reported many times. In 2017, Eunkyoung Kim et al. introduced TiO_2_ nanoparticles (TNP) as a transparent ISL.^422^ This efficiently reduced the working voltage and power consumption of the as-prepared ECD. Moreover, an excellent optical modulation (Δ*T* > 72%) and a significantly improved cyclic life (>3000 cycles with Δ*T* decay <1%) were exhibited. Another classic example is that K. Zaghib et al. achieved a high cyclic life (>200,000 cycles) by using a metal counter electrode.^423^ In recent years, Jianguo Mei’s group reported a series of ion storage materials with no/minimal color change involving organic radical polymers,^138^ amorphous niobium oxide (a-Nb_2_O_5_),^139^ etc.^424^ The ITL is the main source of ions inside the device, and balances the charge by supplying ions to the ECL and ISL. In this layer, the selection of the size and type of electrolyte ions and various auxiliaries (e.g., polymer skeletons), as well as solvents, are essential. For example, it has been reported that the type and concentration of electrolyte ions significantly affects the overpotential of EC materials, and their stability and device performance.^425^
(ii) The microenvironmental and microstructural influences of the related materials and devices should be considered.^426^ During the redox reaction of an active material, the microstructure and size/volume of the material inevitably change due to the change in the molecular energy state and configuration, as well as the insertion/extraction of the relevant ions. This change additionally affects the macroscopic properties. One typical process is the mechanical respiration of EC polymers. Jianguo Mei, Kejie Zhao and coauthors found that this microstructure adjustment could cause a huge volume change (as high as 30%) in the EC polymers (PProDOT).^427^ Based on the obtained mechanical respiratory strain and parameters, the authors drew a phase diagram for reference in R&D. Ultimately, they successfully proposed and verified a new EC film preparation method to toughen the interface through roughening or silica nanoparticle coating surfaces. Based on this, the cyclic life was subsequently increased by more than 2 orders of magnitude.

#### 5.2.2 Processing

The in-depth details of the preparation process should also be considered when manufacturing EC devices and displays. Any small changes in the preparation process and environment, such as the (annealing) temperature,^428^ solvent and volatility,^429^ adhesion of EC materials and electrodes,^430^ and edge sealants of the devices,^431^ can significantly affect the molecular aggregation state and material performance. As an interesting example, Ryan M. Richards and Chaiwat Engtrakul et al. reported the postprocessing ozone technique for improving EC performance.^432^ In this paper, the authors used an aluminum-containing nickel oxide as the EC material and lithium ions as the electrolyte. The induced ozone exposure decreased the crystallinity and increased the nickel oxidation state by introducing hole states. These finally improved the optical modulation and response speed of the as-prepared ECD. Similarly, Chunye Xu’s group reported that Li–Ti codoping can be introduced into NiO-based thin films to enhance the EC properties (optical modulation and stability).^433^

In addition, since most of the reported feasibility studies have been conducted manually under laboratory conditions and environments, there are usually contradictory with large-scale industrialization. For example, inkjet printing technology involves a mature industrial production process, and related researches on EC materials and applications are abundant. However, the compatibility between the printing equipment and EC inks in terms of the material types, diameters, viscosities, and volatilities need to be further studied. In fact, in many cases, to meet the needs of large-scale continuous production and/or environmental protection, we have to replace the original volatile solvents used in laboratories with nonvolatile solvents. Similar problems frequently occur in other industrialization processes of laboratory-based scientific researches, which are also the fundamental reason that most scientific and technological achievements cannot truly become products that meet market demand. Based on this result, it is necessary to deeply understand the source of the above-mentioned inconsistencies and find a way to overcome them. In addition, minimizing the difficulty and complexity of device preparation might be an effective solution also.

#### 5.2.3 Performance

The biggest problem that has led to the unsuccessful commercialization of EC displays is the performance gap. EC materials and devices have attractive advantages in transparency, flexibility, eye friendliness, and other features. However, there are still some performance defects, for example, response speed, color tunability, and even durability. These performance defects are essentially related to materials, devices, and processing techniques. Here, we provide a discussion from the performance perspective.

First, there is still a large gap in response speed compared to the demand for commercial displays. Due to the inevitable electrochemically driven redox process of materials (Faraday process) and ion transfer in the switching of optical states of an EC display, the related response time is difficult to reach below 100 ms. Another reason for this is the optical memory effect. That is to say, additional electrical stimulations are needed to erase the remaining color. Based on this unsatisfactory situation, when scientists and engineers try to promote the commercial application of EC displays, their actual application scenarios should be carefully considered. For example, some low-frequency information refreshing scenes or static display applications (such as e-books, electronic price tags, and billboards), which do not require high response speeds but do require energy-saving and light-absorbing display modes, are likely to be more suitable for EC displays.

Second, there are still no mature EC displays that can realize arbitrary switching and dynamic adjustment of displayed information in the full-color range. Until now, ideal CMYK-based EC materials have not been realized simultaneously. Thus, designing and synthesizing high-performance EC materials with visible spectra comparable to those of commercial CMYK inks is an important research direction. Besides, the successful integration of these EC materials (as CMYK-based subpixels) in one device is another long-term issue. To overcome this problem, the compatibility of newly designed materials with pixelated processes/technologies should be considered and studied in detail.

Most important, according to the “Cannikin law”, the performance of integrated EC displays depends on the worst material used. Thus, in future studies, improving the overall performance is essential.

#### 5.2.4 Durability

In Section 2.2.4, we systematically introduced the topic of durability. To advance the practical application of EC displays, the study of durability is necessary. Taking temperature as an example, it is well-known that temperature has a dramatic effect on the speed of ion mobility. Similar to the fact that the charging and discharging processes of batteries are difficult at low temperatures, the display effect of EC displays at low temperatures needs to be discussed (or improved) also. However, for most reported studies, the corresponding data are generally lacking. This is also understandable because the research and development of EC displays is at a very early stage. However, in the future industrialization process, related testing cannot be ignored.

Taking the lifetime as another example, as far as we know, commercial LCD monitors can generally work more than 40,000 h and even 60,000 h; such capabilities are enough for five to ten years of daily use. With the continuous development of OLED materials and technologies, the related lifetime can reach approximately 30,000 h, representing an increase from the previous 5,000 h. However, the cyclic life of most EC devices (displays) is usually only tens of thousands of cycles or even thousands of cycles, and related data on the working hours are lacking. Obviously, there are still unignorable gaps. Rui-Tao Wen, Claes G. Granqvist, and Gunnar A. Niklasson demonstrated that the EC performance of degraded WO_3_ EC films can be restored by extracting trapped Li^+^ ions.^434,435^ This method is indeed helpful for improving the cyclic life of WO_3_ EC materials. The calendar life, as another important lifetime index, is rarely mentioned but very valuable, although it has been widely accepted in the field of batteries and supercapacitors. Without these data, it is difficult to assess the practical application potential of relevant EC materials/devices and displays.

Moreover, to realize the dynamic and programmatic display/refresh of information, the display needs specific programs (software) and circuits (hardware) to control. However, there are still very few studies focusing on these components, which limit their further commercialization.

### 5.3 Potential Applications of Electrochromic Displays

At present, EC displays are completely different from existing light-emitting displays (e.g., LCDs, LEDs, and OLEDs). For example, the light-absorption/reflection mode of EC displays enables many remarkable features, such as eye friendliness and a wide viewing angle. The low energy consumption of EC displays is also an important feature. Currently, in the preparation process of most EC displays, harmful water and oxygen are not strictly removed, and a solution-processing method is usually adopted, so the process cost is relatively low. However, response speed, lifetime and full-color tunability of existing EC displays are still inferior to those of commercial light-emitting displays. This was discussed many times in this review. The above analysis reveals that the key advantages of EC displays and existing light-emitting displays are not the same. Thus, we strongly believe that they will not be competitive but rather be complementary in the future. This complementarity can be reflected in potential future applications. For example, EC displays can be adapted to electronic labels/tags or large outdoor advertising boards. In this case, the displayed information does not need to be refreshed frequently. Thus, the shortcoming in refresh speed can be efficiently avoided. Moreover, the low energy consumption, low cost, and good outdoor readability are very beneficial. It is obvious that this application is not suitable for existing light-emitting displays.

**Figure 32:**
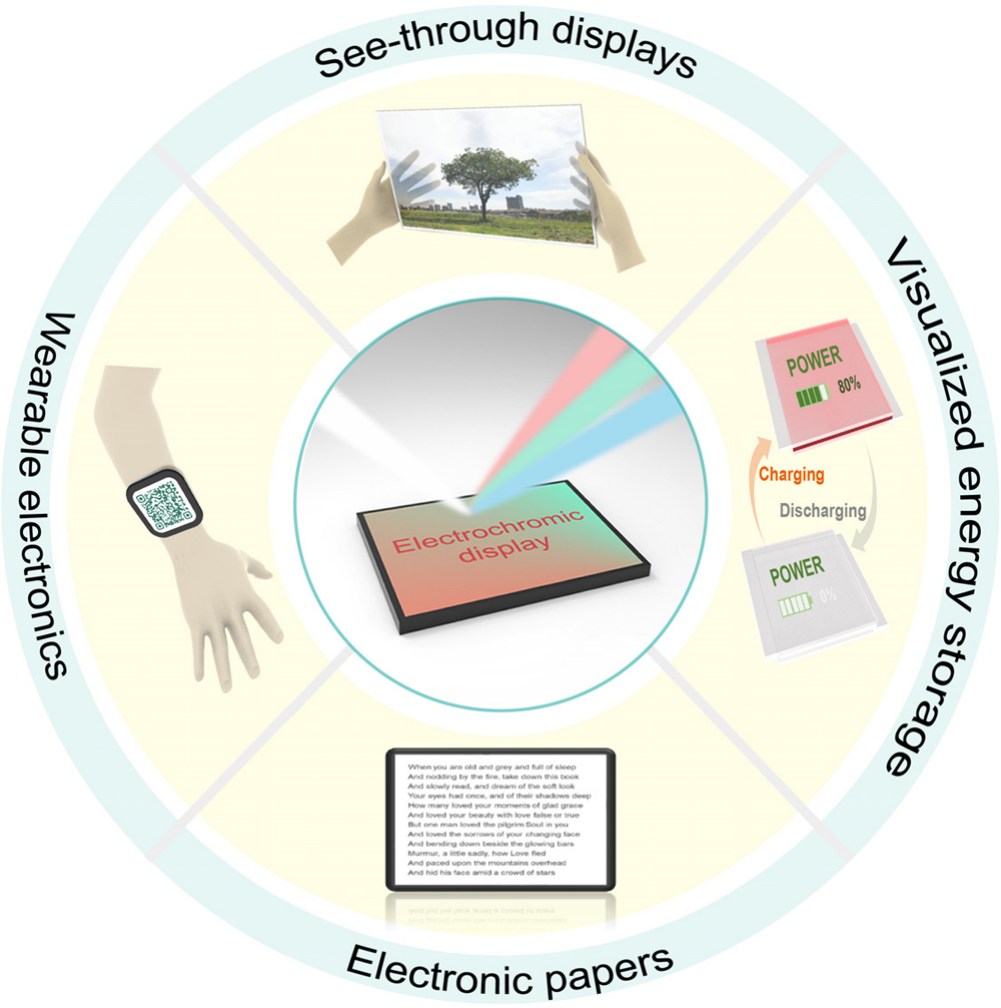
Schematic of potential applications of EC displays.

In the future, the development of EC displays is expected to focus on the following four fields: (i) see-through displays, (ii) wearable electronics, (iii) electronic papers, and (iv) visualized energy storage, as shown in Figure 32.

See-through displays (also known as transparent displays) represent one of the novel technologies that are promising next-generation ubiquitous intelligent displays for future augmented and virtual reality, head-up displays, etc. A see-through display can obtain both the environment information and the displayed information; it saves visual space and improves the efficiency of information exchange/communication. This novel display mode promises to change our daily reading habits. Currently, the commercialization of related products is constantly advancing. For example, many companies have tried to launch consumer electronics products based on transparent displays (Google Glass, OPPO Air Glass, Mi TV LUX see-through television, etc.). In this popular ever-expanding field, due to the susceptibility of light-emitting displays to external environmental disturbances, it is difficult to obtain a good reading experience under strong ambient light (e.g., outdoors). Therefore, the EC display, as a typical nonemissive display with high transparency that can achieve a reversible switch from a transparent colorless state to a transparent colored state, is expected to play a large role in this field.

The second field is wearable electronics. Currently, wearable electronics and electronic skins have been attracting remarkable attention and research interest due to the increasing demand for real-time information communication and human–computer interaction.^12^ Future wearable electronics will be lighter and thinner, more flexible and energy-efficient. Most existing EC materials (especially organic materials) have the features of flexibility, scalability, foldability, and transparency. Moreover, due to the optical memory effect and low driving voltage, their energy consumption is low. In this case, it is reasonable to believe that EC displays will advance the field of wearable electronics.

The third potential application of EC displays is electronic paper. Electronic paper is a very popular electronic reader at present, and it can obtain a reading experience close to that of paper or a book owing to its reflection mode. Additionally, this is a widely accepted eye-friendly display technology. To date, some electronic papers based on electrophoretic displays (e.g., Amazon’s Kindle) have shown very large market potential. However, the colorization of existing electronic papers still faces challenges. ECDs exhibit better color tunability and are fully compatible with the working logic and working mode of electronic papers. In addition, EC devices and displays are expected to be successfully applied in the field of inexpensive electronic tags due to their low power consumption and low process requirements/costs. For example, most non-aqueous ECDs are usually assembled in an air environment and do not require the strict removal of harmful water and oxygen.

The last potential application of EC displays is visualized energy storage. That is, the energy storage status can be visually monitored based on the color differences by combining electrochromism with energy storage. The high degree of conformity in the structures and working principles between ECDs and energy storage devices (especially redox batteries) ensures the feasibility of this concept. In our previous discussion, we presented a large number of academic papers on EC energy storage.^211−214^ The visualized EC energy storage will see considerable development as the energy crisis continues to intensify.

## 6 Conclusions

Considering the potential applications in wearable and portable electronics, paper-like displays, electronic billboards/labels, and so on, potential EC displays have been intensively studied and explored by outstanding researchers for optimizing related performance and preparation technologies. In this review, recent advances in emerging EC materials and devices for better comprehensive performance to meet practical application requirements were introduced systemically. In addition, we discussed two typical display prototypes (segmented displays and pixel displays) in depth. For EC segmented displays, there are two main fabrication methods: electrode patterning and active material patterning. Corresponding materials and processing technologies such as inkjet printing, photolithography, etc., were comprehensively introduced. The arrangement of EC pixels/subpixels and the driving mode were systemically discussed for the fabrication of EC pixel displays. Moreover, combined with commercial EC (displays) products, we analyzed the possible solutions to existing barriers and discussed the future of this field.

Although numerous outstanding scientists and engineers have performed considerable amounts of admirable research and made great progress, EC displays are still in a primary stage and cannot currently meet the market requirements. Unsatisfactory properties, not-yet-realized ideal CMYK-based EC material, and other problems that usually occur in commercial production are the main reasons. Moreover, some important commercialized parameters have not been given much attention, for instance, the temperature tolerance and calendar life. In the future, scientists and engineers need to work together to understand the underlying scientific problem in depth and overcome existing barriers to accelerate the marketization of EC displays and make their application possible in daily life.
